# The contribution of general medical conditions to the non-fatal burden of mental disorders: register-based cohort study in Denmark

**DOI:** 10.1192/bjo.2022.583

**Published:** 2022-10-07

**Authors:** Nanna Weye, Natalie C. Momen, Harvey A. Whiteford, Maria Klitgaard Christensen, Kim Moesgaard Iburg, Damian F. Santomauro, Esben Agerbo, Preben Bo Mortensen, Carsten Bøcker Pedersen, John J. McGrath, Oleguer Plana-Ripoll

**Affiliations:** National Centre for Register-based Research, Aarhus University, Denmark; School of Public Health, The University of Queensland, Australia; and Queensland Centre for Mental Health Research, The Park Centre for Mental Health, Australia; National Centre for Register-based Research, Aarhus University, Denmark; and Department of Public Health, Aarhus University, Denmark; Department of Public Health, Aarhus University, Denmark; School of Public Health, The University of Queensland, Australia; Queensland Centre for Mental Health Research, The Park Centre for Mental Health, Australia; and Institute for Health Metrics and Evaluation, University of Washington, USA; National Centre for Register-based Research, Aarhus University, Denmark; Centre for Integrated Register-based Research (CIRRAU), Aarhus University, Denmark; and Lundbeck Foundation Initiative for Integrative Psychiatric Research (iPSYCH), Aarhus, Denmark; National Centre for Register-based Research, Aarhus University, Denmark; Lundbeck Foundation Initiative for Integrative Psychiatric Research (iPSYCH), Aarhus, Denmark; and Centre for Integrated Register-based Research (CIRRAU), Aarhus University, Denmark; National Centre for Register-based Research, Aarhus University, Denmark; and Centre for Integrated Register-based Research (CIRRAU), Aarhus University, Denmark; National Centre for Register-based Research, Aarhus University, Denmark; Queensland Centre for Mental Health Research, The Park Centre for Mental Health, Australia; and Queensland Brain Institute, The University of Queensland, Australia; National Centre for Register-based Research, Aarhus University, Denmark; and Department of Clinical Epidemiology, Aarhus University and Aarhus University Hospital, Denmark

**Keywords:** Comorbidity, epidemiology, burden of disease, health metrics, disability

## Abstract

**Background:**

General medical conditions (GMCs) often co-occur with mental and substance use disorders (MSDs).

**Aims:**

To explore the contribution of GMCs to the burden of disease in people with MSDs, and investigate how this varied by age.

**Method:**

A population-based cohort of 6 988 507 persons living in Denmark during 2000–2015 followed for up to 16 years. Danish health registers were used to identify people with MSDs and GMCs. For each MSD, years lived with disability and health loss proportion (HeLP) were estimated for comorbid MSDs and GMCs, using a multiplicative model for disability weights.

**Results:**

Those with any MSD lost the equivalent of 43% of healthy life (HeLP = 0.43, 95% CI 0.40–0.44) after including information on GMCs, which was an increase from 25% before including GMCs (HeLP = 0.25, 95% CI 0.23–0.27). Schizophrenia was associated with the highest burden of disease (HeLP = 0.77, 95% CI 0.68–0.85). However, within each disorder, the relative contribution of MSDs and GMCs varied. For example, in those diagnosed with schizophrenia, MSDs and GMCs accounted for 86% and 14% of the total health loss; in contrast, in those with anxiety disorders, the same proportions were 59% and 41%. In general, HeLP increased with age, and was mainly associated with increasing rates of pulmonary, musculoskeletal and circulatory diseases.

**Conclusions:**

In those with mental disorders, the relative contribution of comorbid GMCs to the non-fatal burden of disease increases with age. GMCs contribute substantially to the non-fatal burden of disease in those with MSDs.

The Global Burden of Disease (GBD) studies have established the importance of mental and substance use disorders in health.^1^ Demographic changes related to the ageing population, and better management of infection and malnutrition, will result in non-communicable disorders such as mental disorders and many general medical conditions (GMCs) accounting for a greater proportion of the global burden of disease in the decades ahead. The proportion of global disability-adjusted life-years resulting from the non-fatal burden of disease (as measured by years lived with disability (YLDs)) increased from 20.7% in 1990 to 33.9% in 2019.^1^ Additionally, the rate of YLDs linked to mental and substance use disorders (MSDs) increased by 7% during the same period.^1^ This issue will strain the health services of all nations, including low- and middle-income countries. MSDs accounted for a total of 17.3% of global YLDs in the latest 2019 assessment.^2^ Recently, we used person-level data from Danish registers to estimate YLDs associated with different types of MSDs.^3^ In total, MSDs were associated with 758.6 (95% CI 695.2–830.5) YLDs per 100 000 person-years in the entire Danish population. Apart from confirming the substantial burden of mental disorders, this study was also able to dissect the contribution of each type of mental disorder (i.e. the index disorder) to YLDs versus non-index comorbid mental disorders. To do this, we developed a new health metric, the health loss proportion (HeLP), which focuses on individuals experiencing MSDs instead of the entire population, which is the standard approach to the YLD metric in the GBD studies. The HeLP can be interpreted as the average proportion of health loss that individuals diagnosed with a specific MSD experience because of the index disorder and additional comorbid MSDs. Therefore, HeLP ranges from 0 to 1, with higher proportions indicating greater average disability/health loss. For example, an average person with schizophrenia lost the equivalent of 73% of healthy life per year (HeLP = 0.73, 95% CI 0.63–0.83). Most of this health loss was directly related to schizophrenia (index disorder HeLP = 0.66, 95% CI 0.56–0.76; 90.4% of the total HeLP), and comorbid disorders played a minor role. In contrast, cannabis use disorder had a lower overall health loss (HeLP = 0.43, 95% CI 0.39–0.46) and the majority of this health loss was attributable to comorbidities (index disorder HeLP = 0.06, 95% CI 0.04–0.09; 14.0% of the total).

In addition to comorbidity between different types of MSDs,^4,5^ people with MSDs have an increased risk of subsequently developing a wide range of comorbid GMCs compared with those without mental disorders.^6–8^ Many of these medical conditions (e.g. cancer, ischemic heart disease, musculoskeletal disorders) are also major contributors to YLDs.^2^ However, much less is known about the role of GMC-associated health loss in people with MSDs. We had the opportunity to explore the HeLP for MSDs that take into account both comorbid MSDs and GMCs. We hypothesised that GMCs would make an appreciable contribution to the health loss experienced by those with MSDs, especially in older adults. Based on our findings related to MSDs,^3^ we also speculated that the contribution of GMCs to the HeLP may vary between types of MSDs. The aims of this study were to (a) estimate the total YLDs associated with MSDs, after accounting for comorbidity related to both other MSDs and GMCs; and (b) estimate the contribution of GMCs to the HeLP associated with each MSD, by age.

## Method

### Study population

The study included all persons aged 0–95 years living in Denmark at some point between 1 January 2000 and 31 December 2015. Each individual was followed from birth, immigration to Denmark or 1 January 2000 (whichever happened last) until death, 95th birthday, emigration from Denmark or 31 December 2015 (whichever happened first). All dates were obtained from the Danish Civil Registration System,^9^ which has maintained information on all residents since 1968, including date of birth, gender, continuously updated information on vital status and a unique personal identification number that can be used to link information from various registers. This population register allowed us to include all individuals living in Denmark regardless of whether they received a diagnosis during the study period.

### Assessment of mental and substance disorders

Diagnoses of MSDs were retrieved from the Danish Psychiatric Central Research Register,^10^ which contains data on all admission to psychiatric in-patient facilities since 1969, and visits to out-patient psychiatric departments and emergency departments since 1995. The diagnostic system used was the Danish modification of the ICD-10,^11^ from 1994 onward. MSDs are described in Supplementary Table 1 available at https://doi.org/10.1192/bjo.2022.583, and are based on specific diagnoses used in GBD study publications^12^ and a previous publication of YLDs of mental disorders in Denmark.^3^ MSDs were identified from 1995, and the 5-year period before start of follow-up on 1 January 2000 was used to identify prevalent cases at the beginning of follow-up. For each individual, date of diagnosis for each MSD was defined as admission date for the specific disorder (from in-patient, out-patient or emergency visit).

### Assessment of GMCs

We identified GMCs with an algorithm on data from the Danish National Patient Register^13^ and the Danish National Prescription Registry,^14^ developed to identify the list of chronic GMCs presented in Supplementary Table 2, which consists of 32 diseases and conditions classified into nine broad categories based on the registers.^6,15,16^ The GMC categories were circulatory disorders, endocrine disorders, pulmonary disorders and allergies, gastrointestinal disorders, urogenital disorders, musculoskeletal disorders, haematological disorders, cancers and neurological disorders. For each individual, date of diagnosis of each GMC was defined as first admission date and/or relevant prescription date for the specific disorder.

Because of requirements related to data privacy, comorbid pairs of MSDs and GMCs within a given index that had fewer than five cases were combined with other comorbid conditions within the same broad category (e.g. disorders ischemic stroke and heart failure combined into ‘other’ circulatory diseases) until the combined group (labelled ‘other categories’) consisted of at least five different individuals. In instances where a disease category (e.g. circulatory diseases) consisted of fewer than five cases, the category was grouped with another disease category until the combined group consisted of at least five different individuals (e.g. circulatory diseases and cancers combined into an ‘Other’ category). Combined comorbidities and categories are listed in Supplementary Tables 4–22.

### Remission and duration of each disorder

For this study, we assumed that most GMCs were chronic disorders, and thus we assumed no remission after onset of a GMC. However, when allocating a disability weight to migraine, the GBD studies make a correction for the intermittent nature of this condition (migraine was considered to be symptomatic 8.5% (95% CI 5.8–11.2%) of the year). Date of remission for MSDs was not available through hospital registers. Consistent with our previous publication,^3^ recovery rates were modelled using observed contacts with the healthcare system and gender-, age- and year-specific remission rates for each MSDs in Demark, following GBD study estimates.^17^ In brief, for each date of diagnosis of an MSD, an expected date of remission for that episode was calculated based on the observed date of diagnosis and disorder duration in years following disease-specific remission rates from the GBD studies. In persons with multiple diagnoses for the same disorder, two (or more) episodes were combined into a single one if remission date for the first episode occurred after the onset of the second episode. A detailed description of the application of remission has been published elsewhere.^3^

### Disability weights for each disorder

We used disability weights developed by GBD to quantify health loss associated with a given disease.^18^ Disability weights represent the severity of short- and long-term health loss associated with a given disorder, and were developed based on household and internet-based surveys from nine countries. In these surveys, descriptions of two hypothetical individuals with different health conditions were described briefly in terms of functional effects and symptoms, and respondents were asked to consider which of these individuals the respondent considered to be healthier.^18^ The weights are measured on a scale of 0 to 1, with 0 being a health state that is equivalent to full health and 1 being a health state equivalent to death. For disorders with multiple severity states, a weighted average based on the proportion of cases in each severity state was used. The proportion of cases in each severity was primarily retrieved from the GBD studies.^2^ However, for five GMCs (i.e. diabetes mellitus, chronic obstructive pulmonary disease, cancer, vision problems and hearing problems), proportions were identified in Scottish Burden of Disease publications,^19^ and for three GMCs (ulcer/gastritis, chronic kidney disease and epilepsy), proportions were determined based on other publications.^20–22^ The weighted average disability weight was calculated by summing the product of the multiplication between the proportion of cases for each health state by their corresponding health state disability weight, across all health states within a disorder, and then dividing the sum by the proportions of cases across all health states. As the cases were sampled from hospital registers or based on retrieving prescription drugs, no cases were considered asymptomatic, thus the sum of the proportions could be <1. The weighted disability weight for each MSD and GMC included in this study are described in Supplementary Tables 1 and 2.

### Statistical analysis

The main outcomes of this study were YLDs and HeLP associated with each MSD. This study is a descriptive study and there were no formal tests between specific variables. YLDs and HeLP associated with specific disorders is the share of disability arising from experiencing these disorders. YLDs were estimated as the duration of a disorder (in years) multiplied by the disorder-specific disability weight (summing all individuals at the population level). Rates of YLDs were calculated as the absolute YLDs divided by person-years in the entire population. YLDs were calculated from date of onset of each episode until date of remission, death or 31 December 2015, whichever came first. To account for the fact that some individuals experienced more than one disorder simultaneously, comorbidities were estimated with a multiplicative function, so that no combinations of diseases had a disability weight equal to or greater than 1, which is in line with methods in the GBD studies.^2,23^

HeLP was estimated as YLDs among those experiencing a specific index disorder divided by person-years in those exposed to the index disorder. This estimate can be interpreted as the average proportion of health loss that individuals diagnosed with a specific index MSD experience because of the index disorder and additional comorbid psychiatric and GMCs.

Confidence intervals were estimated by bootstrap with 1000 iterations, which additionally incorporated uncertainty in the disability weights and remission rates, by selecting a random number from a triangular distribution based on these two distributions for each iteration.

The study was approved by the Danish Health Data Authority, Danish Data Protection Agency and Statistics Denmark. All data were de-identified and not recognisable at an individual level. No informed consent is required for a register-based study based on anonymised data, according to Danish law.

## Results

In this study, 6 988 507 persons (3 507 894 females and 3 480 613 males) were followed for 85 898 728 person-years in the period 2000–2015 (the average individual follow-up time was 12.3 years). Of these, 411 443 persons (218 770 females and 192 673 males) were diagnosed with at least one MSD in the observation period. Among persons with any MSD, 304 928 (74%) had at least one diagnosis of a GMC. The most common GMCs were painful conditions (*n* = 161 423), hypertension (*n* = 116 050) and allergy (*n* = 100 492) (Supplementary Table 2).

A total of 581 625 (95% confidence intervals (CI), 528 928–633 434) YLDs were associated with MSDs, which corresponds to a rate of 677 (95% CI 616–737) per 100 000 person-years at the population level and 24 877 (95% CI 22 658–27 093) per 100 000 person-years, when looking only at those experiencing MSDs (Supplementary Table 3). However, when adding YLDs associated with GMCs to the ones associated with MSDs, those diagnosed with an MSD experienced 42 762 (95% CI 40 151–45 223) YLDs per 100 000 person-years, which indicates that they experienced an average health loss of 43% (HeLP = 0.43, 95% CI 0.40–0.44). When focusing on those experiencing specific MSDs, the average health loss ranged from 77% for those with schizophrenia (HeLP = 0.77, 95% CI 0.68–0.85) to 21% for those with attention-deficit hyperactivity disorder (ADHD) (HeLP = 0.21, 95% CI 0.19–0.23). Although ADHD was the only MSD with an average health loss <25%, there were five disorders (schizophrenia and most of the substance use disorders) with a health loss >50%.

The average absolute contribution of the index disorder, other MSDs and GMCs is presented in Figure 1. As exemplified by major depressive disorder (MDD) (Fig. 2), 46.3% of the HeLP for individuals diagnosed with MDD was attributable to the index disorder (MDD), 19.3% was attributable to other MSDs and 34.4% was attributable to comorbid GMCs (pulmonary disorders were the biggest contributor from the GMCs). In general, within the specific categories of GMCs, pulmonary and musculoskeletal disorders were the two largest contributors to the HeLP of the GMCs (Supplementary Figs 1–18).

**Fig. 1:**
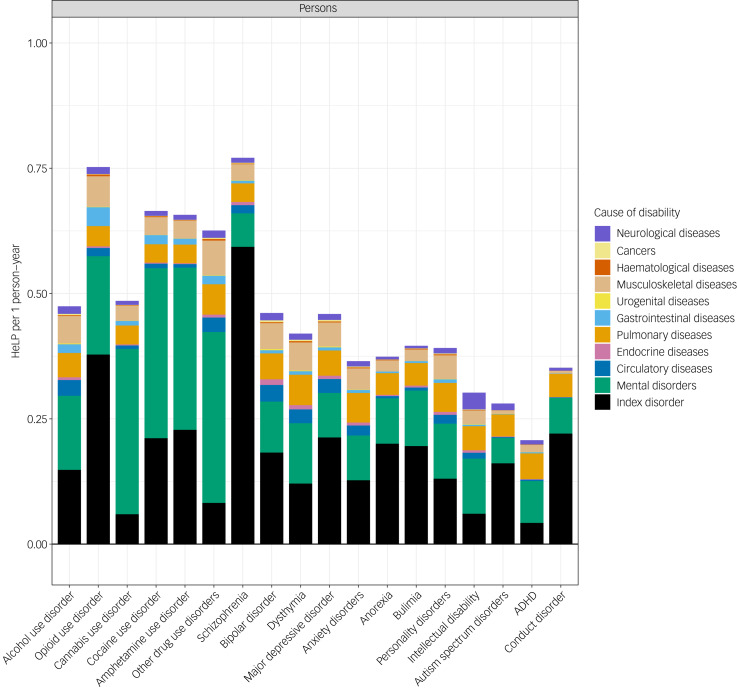
Health loss proportion (HeLP) explained by each index disorder and comorbid conditions per person in the diagnosed subgroup, per year. All estimates are corrected for observed comorbidity from mental and substance use disorders and general medical conditions. ADHD, attention-deficit hyperactivity disorder.

**Fig. 2:**
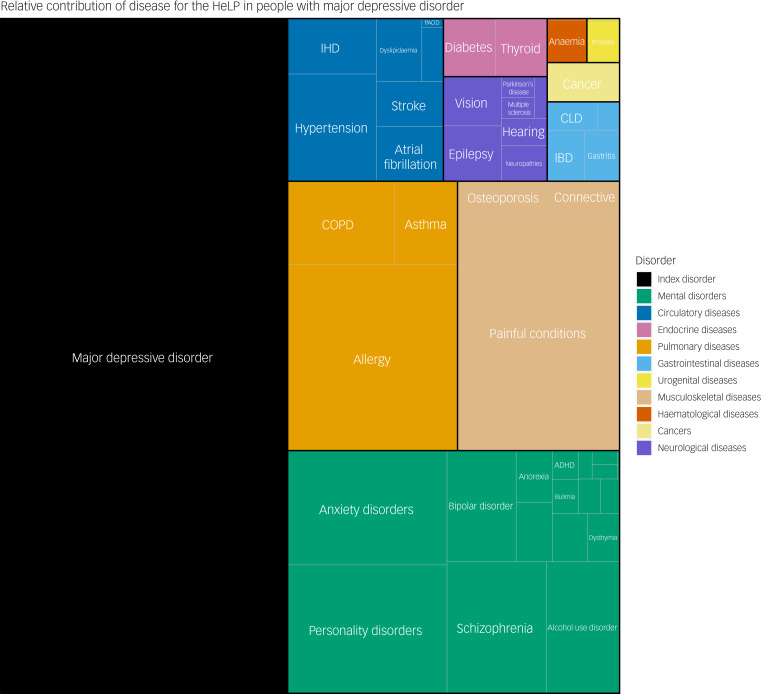
Relative contribution of each mental and substance use disorder and general medical condition to the total health loss proportion (HeLP) for persons diagnosed with major depressive disorder. ADHD, attention-deficit hyperactivity disorder; COPD, chronic obstructive pulmonary disease; CLD, chronic liver disease; IBD, inflammatory bowel disease; IHD, ischemic heart disease; PAOD, peripheral artery occlusive disease.Graphical constraints do not allow us to label all specific subtypes of general medical conditions, but the colour code shows the major general medical condition types.

**Fig. 3:**
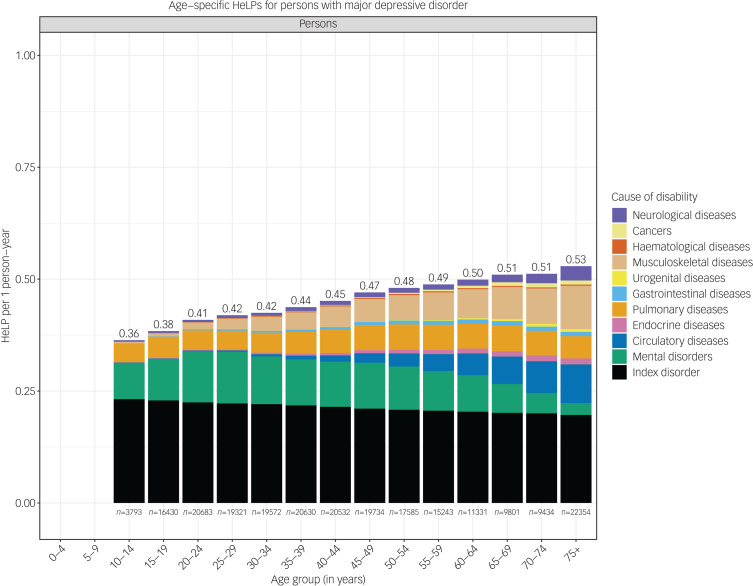
Age-specific health loss proportion (HeLP) for persons diagnosed with major depressive disorder. All estimates are corrected for observed comorbidity from mental and substance use disorders and general medical conditions.

The HeLP increased with age in most MSDs, as exemplified by MDD (Fig. 3). The total HeLP for MDD was 0.36 (95% CI 0.29–0.45) at age 10–14 years, and increased to 0.53 (95% CI 0.47–0.59) at age ≥75 years. The contribution to the total HeLP explained by the index disorder and comorbid MSDs and GMCs varied according to age. In general, individuals experienced a larger number of comorbid conditions with increasing age; thus, the total health loss increased with age. However, because of the use of multiplicative model to combine disability weights from several comorbid conditions, the relative contribution of the index MSD to the total HeLP falls with age. For example, in those with MDD, the disorder accounted for 64.4% of the total HeLP at age 10–14 years, which falls to 37.1% in the ≥75 year age group. Pulmonary disorders contributed to a constant level of HeLP by age, whereas the HeLP associated with circulatory and musculoskeletal disorders increased with age. In addition, the HeLP associated with other MSDs increased until age 25–29 years – at which point the incidence for GMCs starts to increase – and decreased substantially afterward.

## Discussion

Our study provides the first reported details on the contribution of MSDs and comorbid GMCs to the individual non-fatal burden of disease. Based on a ‘bottom-up’ approach, we have estimated person-level YLDs for mental disorders after taking into account comorbid GMCs. In this discussion, we will concentrate on three key findings. First, GMCs contribute substantially to the non-fatal burden of disease in those with MSDs. Second, the relative contribution of GMCs to health loss increased with age. Third, the HeLP provides insights into the relative contribution of comorbidity to overall health loss, which can serve as a template for future research based on a wider range of disorders.

In our recent YLD study, we confirmed the contribution of different types of MSD to YLDs.^3^ Here, we show how the inclusion of GMCs increased the total health loss in those who experience MSDs. For those with any MSD, we observed an increase of 58% in the HeLP after including GMCs (HeLP = 0.43), compared with the HeLP before including GMCs (HeLP = 0.28). When looking at specific disorders, the increase in HeLP associated with GMCs ranged from 8% for schizophrenia (MSD-corrected HeLP = 0.73, MSD- and GMC-corrected HeLP = 0.77) to 62% for ADHD (MSD-corrected HeLP = 0.13, MSD- and GMC-corrected HeLP = 0.21). Across different types of index MSDs, other comorbid MSDs accounted for the largest share of HeLP associated with comorbidity, whereas pulmonary and musculoskeletal disorders were the leading causes of HeLP among GMCs. Although musculoskeletal disorders have disability weights that are lower compared with other GMCs, they are prevalent disorders and the accumulated disability is therefore much higher. These findings corroborate recent research^6–8^ that found extensive comorbidity between MSDs and GMCs, and extend these findings into measures of health loss. Our results complement prior work by Lokkerbol et al, who explored a metric related with HeLP based on self-reported somatic diseases.^24^

Second, in keeping with the higher prevalence of GMCs in the elderly,^25^ our study confirms that the contribution of GMCs to the HeLP increases with age in a mostly linear trend. Across disorders, a distinct pattern emerged. In childhood and adolescence, the leading cause of HeLP among GMCs was pulmonary disorders, which remained a steady contributor to HeLP throughout the lifespan. From age 30 years onward, the HeLP associated with musculoskeletal disorders increased, as did the HeLP associated with circulatory disorders after age 50 years.

Finally, our new HeLP metric provides a useful way to dissect the contribution of the index disorder and other comorbid disorders. Our graphical displays provide a simple but informative way to explore the contribution of different disorders to the non-fatal burden of disease. Although the GBD studies do account for comorbidity in the modelling, the approach assumes independent comorbidity (i.e. the model assumes all persons with an index disorder have the same risk of a comorbid disorder, independent of age, gender and time since index diagnosis). In contrast, the person-level register data allowed us to observe precise combinations of disease, and estimate health loss associated with these combinations. A recent commentary by Das-Munshi and Prina noted that the HeLP may also be of interest to services with electronic health records, as a way to explore the impact of comorbidity on health loss.^26^

### Strengths and limitations of the study

The main strength of this study is the population-based design, including the use of population-based registers in Denmark. These allow for the inclusion of the entire population and almost complete follow-up retention, and contain prospectively collected data on MSDs and GMCs. However, several limitations should be noted. Although many common disorders were included in our set of GMCs, the YLD estimates may be overestimated because we did not include all possible combinations of disorders, whereas the HeLP will be underestimated. In addition, we did not have information about undiagnosed diseases, diagnoses before 1995 or persons treated for MSDs or GMCs by their general practitioner. However, in some GMCs, information on dispensed prescriptions allowed for the inclusion of diseases treated outside a hospital setting. We used weighted disability weights based on severity distributions, many of which were from the GBD studies. These distributions were not specific to Denmark, and the use of worldwide proportions can lead to both under- and overestimation of YLDs;^27^ this would depend on the disorder of interest, although the direction of the misclassification could not be determined in this study. We made the simplifying assumption that the GMCs included in the current study did not recover. Although we were able to use a GBD study correction for the intermittent nature of migraine, we were not able to model recovery for all GMCs. Thus, we may have overestimated the YLDs associated with some of the GMCs included in this study. Although information on MSDs and GMCs relied on clinical diagnoses instead of direct structured diagnostic interviews, many register-based diagnoses have been confirmed to have good validity.^28–31^ However, many of the disorders examined in this study require clinical validation studies. In the current study, we have focused on the YLD burden in those with mental disorders; it would be of interest to explore how these estimates compare to a matched population with similar GMCs, but without mental disorders. Finally, although the key findings of this study may be broadly generalisable to other high-income countries, this may not be the case for low- and middle-income countries.

As has been recently pointed out,^1^ disability related to non-communicable disorders, including MSDs, will figure more prominently in future disease burden. In the absence of more effective interventions, we can expect that MSDs and related comorbidities will continue to be major drivers of overall health expenditure. The HeLP is an informative and complementary health metric that can enrich our understanding of the impact of comorbidity on non-fatal disease burden.

## Data Availability

The data used for this study are not publicly available, but can be obtained by application to The Danish Health Data Authority (www.sundhedsdatastyrelsen.dk).

## Supplementary material

For supplementary material accompanying this paper visit http://doi.org/10.1192/bjo.2022.583. click here to view supplementary material
