# Supplementary material for: The contribution of general medical conditions to the non-fatal burden of mental disorders: register-based cohort study in Denmark
Source: BJPsych Open. 2022 Oct 7;8(6):e180. doi: 10.1192/bjo.2022.583 (PMC9634585; doi:10.1192/bjo.2022.583)
Supplement: Supplementary file 1 [file S205647242200583Xsup001.docx]

**Supplementary material**

**Supplementary table 1**. ICD-10 codes, age of onset and associated disability weight^1^ for each specific mental and substance use disorder.

| **Disorders** | **ICD-10 codes** | **Earliest age of onset**  **(Years)** | **Disability weight** |
| --- | --- | --- | --- |
| Alcohol use disorder | F10.2 | 10 | 0.18 (0.11-0.27) |
| Opioid use disorder | F11.2 | 10 | 0.54 (0.39-0.67) |
| Cannabis use disorder | F12.2 | 10 | 0.07 (0.04-0.11) |
| Cocaine use disorder | F14.2 | 10 | 0.30 (0.20-0.40) |
| Amphetamine use disorder | F15.2 | 10 | 0.31 (0.21-0.41) |
| Other drug use disorders | F13.2, F16.2, F18.2 | 10 | 0.12 (0.07-0.17) |
| Schizophrenia | F20 | 10 | 0.71 (0.56-0.84) |
| Bipolar disorder | F30, F31 (except F31.7), F34.0 | 10 | 0.22 (0.15-0.31) |
| Major depressive disorder | F32, F33 | 10 | 0.25 (0.15-0.38) |
| Dysthymia | F34.1 | 10 | 0.15 (0.10-0.21) |
| Anxiety disorders | F40, F41, F42, F43.1, F93.0-F93.2, F93.8 | 1 | 0.15 (0.09-0.21) |
| Anorexia nervosa | F50.0, F50.1 | 1 | 0.22 (0.15-0.31) |
| Bulimia nervosa | F50.2 | 1 | 0.22 (0.15-0.31) |
| Personality disorders | F60 | 10 | 0.15 (0.10-0.21) |
| Idiopathic developmental  intellectual disability | F70-F79, Z81.0 | 1 | 0.07 (0.03-0.15) |
| Autism spectrum disorder | F84 | 1 | 0.17 (0.11-0.24) |
| ADHD | F90 | 1 | 0.05 (0.03-0.07) |
| Conduct disorder | F91.0-F91.2, F91.8 | 1 | 0.24 (0.16-0.34) |

**Supplementary table 2**. ICD-10 codes and associated disability weight ^1^ for each specific general medical condition.

| **Category** | **Disorder** | **Coding definition** | **ICD-10 codes** | **Medications** | | **Cases among those with a mental disorder** | **Disability weight** |
| --- | --- | --- | --- | --- | --- | --- | --- |
|  |  |  |  | **ATC codes** | **Time frame for prescriptions** |  |  |
| Circulatory system (N= 146,140) | Hypertension | Diagnosis AND/OR prescriptions of antihypertensives (a) | I10-I13, I15 | C02, C03, C04, C07, C08, C09 | Twice in 1 year | 116,050 | 0.049 (0.031-0.072) |
|  | Dyslipidemia | Diagnosis AND/OR prescriptions of lipid-lowering drugs (b) | E78 | C10 | Twice in 1 year | 47,647 | 0.049 (0.031-0.072) |
|  | Ischemic heart disease | Diagnosis AND/OR prescription for antianginal drug | I20-I25 | C01DA | Twice in 1 year | 31,818 | 0.108 (0.074-0.152) |
|  | Atrial fibrillation | Diagnosis | I48 |  |  | 14,691 | 0.224 (0.151-0.315) |
|  | Heart failure | Diagnosis | I50 |  |  | 12,712 | 0.092 (0.062-0.131) |
|  | Peripheral artery occlusive disease | Diagnosis | I70-I74 |  |  | 11,569 | 0.014 (0.007-0.025) |
|  | Ischemic stroke | Diagnosis | I60-I64, I69 |  |  | 23,937 | 0.104 (0.045-0.181) |
| Endocrine (N= 57,773) | Diabetes | Diagnosis AND/OR prescription of antidiabetics | E10-E14 | A10A, A10B | Twice in 1 year | 32,733 | 0.077 (0.051-0.111) |
|  | Thyroid disorder | Diagnosis AND/OR prescription of thyroid therapy drugs | E00-E05, E06.1-E06.9, E07 | H03 | Twice in 1 year | 27,546 | 0.066 (0.040-0.101) |
|  | Gout | Diagnosis | E79, M10 |  |  | 2621 | 0.023 (0.014-0.034) |
| Pulmonary system and allergy (N= 170,102) | Asthma | Diagnosis AND/OR Prescription for obstructive airway disease drugs | J45-J46 | R03 | Twice in 1 year | 68,983 | 0.065 (0.043-0.095) |
|  | Chronic obstructive pulmonary disease | Diagnosis AND/OR Prescription for obstructive airway disease drugs | J40-J44, J47 | R03 | Twice in 1 year | 53,710 | 0.126 (0.077-0.177) |
|  | Allergy | Diagnosis AND/OR Prescription for non-sedative antihistamines AND/OR nasal antiallergics | J30.1-J30.4 , L23, L50.0, T78.0. T78.2, T78.4 | R06AX, R06AE07, E06AE09, R01AC, R01AD | Twice in 1 year | 100,492 | 0.218 (0.138-0.308) |
| Gastrointestinal system  (N= 41,295) | Ulcer/chronic gastritis | Diagnosis | K22.1, K25-K28, K29.3-K29.5 |  |  | 18,515 | 0.074 (0.040-0.086) |
|  | Chronic liver disease | Diagnosis | B16-B19, K70, K74, K76.6, I85 |  |  | 12,987 | 0.178 (0.123-0.243) |
|  | Inflammatory bowel disease | Diagnosis | K50-K51 |  |  | 5758 | 0.231 (0.156-0.320) |
|  | Diverticular disease of intestine | Diagnosis | K57 |  |  | 9352 | 0.049 (0.031-0.072) |
| Urogenital system  (N= 18,381) | Chronic kidney disease | Diagnosis | N03, N11, N18-N19 |  |  | 6344 | 0.016 (0.010-0.024) |
|  | Prostate disorders | Diagnosis AND/OR Prescription of prostate hyperplasia therapy drugs | N40 | C02CA, G04C | Twice in 1 year | 13,019 | 0.067 (0.043-0.097) |
| Musculoskeletal system  (N= 167,229) | Connective tissue disorders | Diagnosis | M05-M06, M08-M09, M30-M36, D86 |  |  | 11,316 | 0.131 (0.097-0.160) |
|  | Osteoporosis | Diagnosis AND/OR prescription for osteoporosis drugs | M80-M82 | M05B, G03XC01, H05AA | Twice in 1 year | 18,040 | 0.131 (0.097-0.160) |
|  | Painful conditions | Repeated prescriptions (four times within 12 months) of analgesics |  | N02A, N02BA51, N02BE, M01A, M02A | 4 times in 1 year | 161,423 | 0.159 (0.100-0.222) |
| Hematological system (N= 42,744) | HIV/AIDS | Diagnosis | B20-B24 |  |  | 919 | 0.049 (0.031-0.072) |
|  | Anemias | Diagnosis | D50-D53, D55-D59, D60-D61, D63-D64 |  |  | 41,985 | 0.028 (0.018-0.043) |
| Cancers  (N=25,511) | Cancer | Diagnosis | C00-C43, C45-C97 |  |  | 25,511 | 0.105 (0.102-0.108) |
| Neurological system (N= 85,752) | Vision problems | Diagnosis | H40, H25, H54 |  |  | 23,796 | 0.085 (0.055-0.121) |
|  | Hearing problems | Diagnosis | H90-H91, H931 |  |  | 25,924 | 0.029 (0.016-0.045) |
|  | Migraine | Diagnosis AND/OR prescription of specific anti-migraine drugs | G43 | N02C | Twice in 1 year | 9095 | 0.037 (0.017-0.066) |
|  | Epilepsy | Diagnosis AND prescription of anti-epileptics | G40-G41 | N03 | Twice in 1 year | 14,239 | 0.267 (0.179-0.355) |
|  | Parkinson's disease | Diagnosis | G20-G22 |  |  | 2951 | 0.166 (0.110-0.233) |
|  | Multiple sclerosis | Diagnosis | G35 |  |  | 1431 | 0.366 (0.251-0.485) |
|  | Neuropathies | Diagnosis | G50-G64 |  |  | 25,791 | 0.049 (0.031-0.072) |

**Supplementary table 3**. Number of cases, absolute disorder-specific years lived with disability (YLDs), rate of YLDs per 100,000 persons-years in the entire population and rate of YLDs per 100,000 person-years in the exposed population, in Denmark, years 2000-2015. Absolute YLDs and rates are adjusted for observed comorbidity from mental and substance use disorders and general medical conditions.

| **Disorder** | **N** | **YLDs** | **Rate of YLDs per 100,000 person-years** | **Rate of YLDs per 100,000 person-years in the exposed population** |
| --- | --- | --- | --- | --- |
| Alcohol use disorder | 51738 | 32,756 (23,834-45,379) | 38 (28-53) | 14,811 (10,617-20,262) |
| Opioid use disorder | 5621 | 13,868 (11,099-16,707) | 16 (13-19) | 37,819 (30,355-45,182) |
| Cannabis use disorder | 15522 | 4,235 (2,926-6,046) | 5 (3-7) | 5,961 (4,054-8,413) |
| Cocaine use disorder | 1804 | 1,940 (1,457-2,502) | 2 (2-3) | 21,141 (15,651-26,358) |
| Amphetamine use disorder | 2369 | 2,197 (1,717-2,768) | 3 (2-3) | 22,806 (17,488-27,777) |
| Other drug use disorders | 6189 | 413 (295-540) | 0 (0-1) | 8,213 (5,900-10,757) |
| Schizophrenia | 39093 | 210,921 (178,403-241,609) | 246 (208-281) | 59,315 (50,154-67,719) |
| Bipolar disorder | 28338 | 36,483 (27,075-47,074) | 42 (32-55) | 18,260 (13,536-23,494) |
| Dysthymia | 6762 | 4,363 (3,249-5,669) | 5 (4-7) | 12,087 (9,060-15,809) |
| Major depressive disorder | 169950 | 41,094 (28,412-55,987) | 48 (33-65) | 21,289 (14,521-28,894) |
| Anxiety disorders | 109900 | 70,353 (50,967-93,726) | 82 (59-109) | 12,744 (9,263-16,914) |
| Anorexia | 9541 | 8,749 (6,397-11,434) | 10 (7-13) | 20,033 (14,828-25,826) |
| Bulimia | 5132 | 3,628 (2,721-4,858) | 4 (3-6) | 19,557 (14,646-25,247) |
| Personality disorders | 83491 | 97,501 (70,476-126,176) | 114 (82-147) | 13,051 (9,446-16,890) |
| Intellectual disability | 22539 | 10,939 (4,425-20,831) | 13 (5-24) | 6,060 (2,452-11,536) |
| Autism spectrum disorders | 30573 | 31,201 (22,877-37,995) | 36 (27-44) | 16,122 (11,754-19,733) |
| ADHD | 46540 | 9,716 (6,803-13,134) | 11 (8-15) | 4,228 (2,953-5,730) |
| Conduct disorder | 1963 | 1,268 (960-1,730) | 1 (1-2) | 22,053 (16,725-29,146) |
| Any mental or substance use disorder | 414,854 | 581,625 (528,928-633,434) | 677 (616-737) | 24,877 (22,658-27,093) |

**Supplementary Figure 1**. Relative contribution of each mental and substance use disorder and general medical condition to the total Health Loss Proportion (HeLP) for persons diagnosed with alcohol use disorder.

AF: atrial fibrillation, COPD: chronic obstructive pulmonary disease, diverticular: diverticular disease of intestine, HF: heart failure, IBD: inflammatory bowel disease, liver: chronic liver disease IHD: ischemic heart disease, MS: multiple sclerosis, Painful: painful conditions, PAOD: peripheral artery occlusive disease.

**Supplementary Figure 2**. Relative contribution of each mental and substance use disorder and general medical condition to the total Health Loss Proportion (HeLP) for persons diagnosed with opioid use disorder.

AF: atrial fibrillation, COPD: chronic obstructive pulmonary disease, diverticular: diverticular disease of intestine, HF: heart failure, IBD: inflammatory bowel disease, liver: chronic liver disease IHD: ischemic heart disease, MS: multiple sclerosis, Painful: painful conditions, PAOD: peripheral artery occlusive disease.

**Supplementary Figure 3**. Relative contribution of each mental and substance use disorder and general medical condition to the total Health Loss Proportion (HeLP) for persons diagnosed with cannabis use disorder.

AF: atrial fibrillation, COPD: chronic obstructive pulmonary disease, diverticular: diverticular disease of intestine, HF: heart failure, IBD: inflammatory bowel disease, liver: chronic liver disease IHD: ischemic heart disease, MS: multiple sclerosis, Painful: painful conditions, PAOD: peripheral artery occlusive disease.

**Supplementary Figure 4**. Relative contribution of each mental and substance use disorder and general medical condition to the total Health Loss Proportion (HeLP) for persons diagnosed with cocaine use disorder.

AF: atrial fibrillation, COPD: chronic obstructive pulmonary disease, diverticular: diverticular disease of intestine, HF: heart failure, IBD: inflammatory bowel disease, liver: chronic liver disease IHD: ischemic heart disease, MS: multiple sclerosis, Painful: painful conditions, PAOD: peripheral artery occlusive disease.

**Supplementary Figure 5**. Relative contribution of each mental and substance use disorder and general medical condition to the total Health Loss Proportion (HeLP) for persons diagnosed with amphetamine use disorder.

AF: atrial fibrillation, COPD: chronic obstructive pulmonary disease, diverticular: diverticular disease of intestine, HF: heart failure, IBD: inflammatory bowel disease, liver: chronic liver disease IHD: ischemic heart disease, MS: multiple sclerosis, Painful: painful conditions, PAOD: peripheral artery occlusive disease.

**Supplementary Figure 6**. Relative contribution of each mental and substance use disorder and general medical condition to the total Health Loss Proportion (HeLP) for persons diagnosed with other drug use disorders.

AF: atrial fibrillation, COPD: chronic obstructive pulmonary disease, diverticular: diverticular disease of intestine, HF: heart failure, IBD: inflammatory bowel disease, liver: chronic liver disease IHD: ischemic heart disease, MS: multiple sclerosis, Painful: painful conditions, PAOD: peripheral artery occlusive disease.

**Supplementary Figure 7**. Relative contribution of each mental and substance use disorder and general medical condition to the total Health Loss Proportion (HeLP) for persons diagnosed with schizophrenia.

AF: atrial fibrillation, COPD: chronic obstructive pulmonary disease, diverticular: diverticular disease of intestine, HF: heart failure, IBD: inflammatory bowel disease, liver: chronic liver disease IHD: ischemic heart disease, MS: multiple sclerosis, Painful: painful conditions, PAOD: peripheral artery occlusive disease.

**Supplementary Figure 8**. Relative contribution of each mental and substance use disorder and general medical condition to the total Health Loss Proportion (HeLP) for persons diagnosed with bipolar disorder.

AF: atrial fibrillation, COPD: chronic obstructive pulmonary disease, diverticular: diverticular disease of intestine, HF: heart failure, IBD: inflammatory bowel disease, liver: chronic liver disease IHD: ischemic heart disease, MS: multiple sclerosis, Painful: painful conditions, PAOD: peripheral artery occlusive disease.

**Supplementary Figure 9**. Relative contribution of each mental and substance use disorder and general medical condition to the total Health Loss Proportion (HeLP) for persons diagnosed with dysthymia.

AF: atrial fibrillation, COPD: chronic obstructive pulmonary disease, diverticular: diverticular disease of intestine, HF: heart failure, IBD: inflammatory bowel disease, liver: chronic liver disease IHD: ischemic heart disease, MS: multiple sclerosis, Painful: painful conditions, PAOD: peripheral artery occlusive disease.

**Supplementary Figure 10**. Relative contribution of each mental and substance use disorder and general medical condition to the total Health Loss Proportion (HeLP) for persons diagnosed with major depressive disorder.

AF: atrial fibrillation, COPD: chronic obstructive pulmonary disease, diverticular: diverticular disease of intestine, HF: heart failure, IBD: inflammatory bowel disease, liver: chronic liver disease IHD: ischemic heart disease, MS: multiple sclerosis, Painful: painful conditions, PAOD: peripheral artery occlusive disease.

**Supplementary Figure 11**. Relative contribution of each mental and substance use disorder and general medical condition to the total Health Loss Proportion (HeLP) for persons diagnosed with anxiety disorders.

AF: atrial fibrillation, COPD: chronic obstructive pulmonary disease, diverticular: diverticular disease of intestine, HF: heart failure, IBD: inflammatory bowel disease, liver: chronic liver disease IHD: ischemic heart disease, MS: multiple sclerosis, Painful: painful conditions, PAOD: peripheral artery occlusive disease.

**Supplementary Figure 12**. Relative contribution of each mental and substance use disorder and general medical condition to the total Health Loss Proportion (HeLP) for persons diagnosed with anorexia.

AF: atrial fibrillation, COPD: chronic obstructive pulmonary disease, diverticular: diverticular disease of intestine, HF: heart failure, IBD: inflammatory bowel disease, liver: chronic liver disease IHD: ischemic heart disease, MS: multiple sclerosis, Painful: painful conditions, PAOD: peripheral artery occlusive disease.

**Supplementary Figure 13**. Relative contribution of each mental and substance use disorder and general medical condition to the total Health Loss Proportion (HeLP) for persons diagnosed with bulimia.

AF: atrial fibrillation, COPD: chronic obstructive pulmonary disease, diverticular: diverticular disease of intestine, HF: heart failure, IBD: inflammatory bowel disease, liver: chronic liver disease IHD: ischemic heart disease, MS: multiple sclerosis, Painful: painful conditions, PAOD: peripheral artery occlusive disease.

**Supplementary Figure 14**. Relative contribution of each mental and substance use disorder and general medical condition to the total Health Loss Proportion (HeLP) for persons diagnosed with personality disorders.

AF: atrial fibrillation, COPD: chronic obstructive pulmonary disease, diverticular: diverticular disease of intestine, HF: heart failure, IBD: inflammatory bowel disease, liver: chronic liver disease IHD: ischemic heart disease, MS: multiple sclerosis, Painful: painful conditions, PAOD: peripheral artery occlusive disease.

**Supplementary Figure 15**. Relative contribution of each mental and substance use disorder and general medical condition to the total Health Loss Proportion (HeLP) for persons diagnosed with intellectual disability.

AF: atrial fibrillation, COPD: chronic obstructive pulmonary disease, diverticular: diverticular disease of intestine, HF: heart failure, IBD: inflammatory bowel disease, liver: chronic liver disease IHD: ischemic heart disease, MS: multiple sclerosis, Painful: painful conditions, PAOD: peripheral artery occlusive disease.

**Supplementary Figure 16**. Relative contribution of each mental and substance use disorder and general medical condition to the total Health Loss Proportion (HeLP) for persons diagnosed with autism spectrum disorder.

AF: atrial fibrillation, COPD: chronic obstructive pulmonary disease, diverticular: diverticular disease of intestine, HF: heart failure, IBD: inflammatory bowel disease, liver: chronic liver disease IHD: ischemic heart disease, MS: multiple sclerosis, Painful: painful conditions, PAOD: peripheral artery occlusive disease.

**Supplementary Figure 17**. Relative contribution of each mental and substance use disorder and general medical condition to the total Health Loss Proportion (HeLP) for persons diagnosed with ADHD.

AF: atrial fibrillation, COPD: chronic obstructive pulmonary disease, diverticular: diverticular disease of intestine, HF: heart failure, IBD: inflammatory bowel disease, liver: chronic liver disease IHD: ischemic heart disease, MS: multiple sclerosis, Painful: painful conditions, PAOD: peripheral artery occlusive disease.

**Supplementary Figure 18**. Relative contribution of each mental and substance use disorder and general medical condition to the total Health Loss Proportion (HeLP) for persons diagnosed with conduct disorder.

AF: atrial fibrillation, COPD: chronic obstructive pulmonary disease, diverticular: diverticular disease of intestine, HF: heart failure, IBD: inflammatory bowel disease, liver: chronic liver disease IHD: ischemic heart disease, MS: multiple sclerosis, Painful: painful conditions, PAOD: peripheral artery occlusive disease.

**Supplementary Figure 19**. Age-specific Health Loss Proportion (HeLP) for persons diagnosed with alcohol use disorder. All estimates are adjusted for observed comorbidity from mental and substance use disorders and general medical conditions.

**Supplementary Figure 20**. Age-specific Health Loss Proportion (HeLP) for persons diagnosed with opioid use disorder. All estimates are adjusted for observed comorbidity from mental and substance use disorders and general medical conditions.

**Supplementary Figure 21**. Age-specific Health Loss Proportion (HeLP) for persons diagnosed with cannabis use disorder. All estimates are adjusted for observed comorbidity from mental and substance use disorders and general medical conditions.

**Supplementary Figure 22**. Age-specific Health Loss Proportion (HeLP) for persons diagnosed with cocaine use disorder. All estimates are adjusted for observed comorbidity from mental and substance use disorders and general medical conditions.

**Supplementary Figure 23**. Age-specific Health Loss Proportion (HeLP) for persons diagnosed with amphetamine use disorder. All estimates are adjusted for observed comorbidity from mental and substance use disorders and general medical conditions.

**Supplementary Figure 24**. Age-specific Health Loss Proportion (HeLP) for persons diagnosed with other drug use disorders. All estimates are adjusted for observed comorbidity from mental and substance use disorders and general medical conditions.

**Supplementary Figure 25**. Age-specific Health Loss Proportion (HeLP) for persons diagnosed with schizophrenia. All estimates are adjusted for observed comorbidity from mental and substance use disorders and general medical conditions.

**Supplementary Figure 26**. Age-specific Health Loss Proportion (HeLP) for persons diagnosed with bipolar disorder. All estimates are adjusted for observed comorbidity from mental and substance use disorders and general medical conditions.

**Supplementary Figure 27**. Age-specific Health Loss Proportion (HeLP) for persons diagnosed with dysthymia. All estimates are adjusted for observed comorbidity from mental and substance use disorders and general medical conditions.

**Supplementary Figure 28.** Age-specific Health Loss Proportion (HeLP) for persons diagnosed with major depressive disorder. All estimates are adjusted for observed comorbidity from mental and substance use disorders and general medical conditions.

**Supplementary Figure 29**. Age-specific Health Loss Proportion (HeLP) for persons diagnosed with anxiety disorders. All estimates are adjusted for observed comorbidity from mental and substance use disorders and general medical conditions.

**Supplementary Figure 30**. Age-specific Health Loss Proportion (HeLP) for persons diagnosed with anorexia. All estimates are adjusted for observed comorbidity from mental and substance use disorders and general medical conditions.

**Supplementary Figure 31**. Age-specific Health Loss Proportion (HeLP) for persons diagnosed with bulimia. All estimates are adjusted for observed comorbidity from mental and substance use disorders and general medical conditions.

**Supplementary Figure 32**. Age-specific Health Loss Proportion (HeLP) for persons diagnosed with personality disorders. All estimates are adjusted for observed comorbidity from mental and substance use disorders and general medical conditions.

**Supplementary Figure 33**. Age-specific Health Loss Proportion (HeLP) for persons diagnosed with intellectual disability. All estimates are adjusted for observed comorbidity from mental and substance use disorders and general medical conditions.

**Supplementary Figure 34**. Age-specific Health Loss Proportion (HeLP) for persons diagnosed with autism spectrum disorders. All estimates are adjusted for observed comorbidity from mental and substance use disorders and general medical conditions.

**Supplementary Figure 35**. Age-specific Health Loss Proportion (HeLP) for persons diagnosed with ADHD. All estimates are adjusted for observed comorbidity from mental and substance use disorders and general medical conditions.

**Supplementary Figure 36**. Age-specific Health Loss Proportion (HeLP) for persons diagnosed with conduct disorder. All estimates are adjusted for observed comorbidity from mental and substance use disorders and general medical conditions.

**Supplementary table 4.** Health Loss Proportion (HeLP) and 95% confidence interval for persons, by disorder and comorbid disorder. All estimates are adjusted for observed comorbidity from mental and substance use disorders and general medical conditions.

| **Disorder** | **Category** | **Comorbidity** | **HeLP** |
| --- | --- | --- | --- |
| Alcohol use disorder | Index disorder | Alcohol use disorder | 0.15 (0.11-0.20) |
| Alcohol use disorder | Mental disorders | Opioid use disorder | 0.01 (0.01-0.01) |
| Alcohol use disorder | Mental disorders | Cannabis use disorder | 0.00 (0.00-0.00) |
| Alcohol use disorder | Mental disorders | Cocaine use disorder | 0.00 (0.00-0.00) |
| Alcohol use disorder | Mental disorders | Amphetamine use disorder | 0.00 (0.00-0.00) |
| Alcohol use disorder | Mental disorders | Other drug use disorders | 0.00 (0.00-0.00) |
| Alcohol use disorder | Mental disorders | Schizophrenia | 0.06 (0.05-0.07) |
| Alcohol use disorder | Mental disorders | Bipolar disorder | 0.01 (0.01-0.02) |
| Alcohol use disorder | Mental disorders | Dysthymia | 0.00 (0.00-0.00) |
| Alcohol use disorder | Mental disorders | Major depressive disorder | 0.01 (0.01-0.02) |
| Alcohol use disorder | Mental disorders | Anxiety disorders | 0.01 (0.01-0.02) |
| Alcohol use disorder | Mental disorders | Anorexia | 0.00 (0.00-0.00) |
| Alcohol use disorder | Mental disorders | Bulimia | 0.00 (0.00-0.00) |
| Alcohol use disorder | Mental disorders | Personality disorders | 0.02 (0.02-0.03) |
| Alcohol use disorder | Mental disorders | Intellectual disability | 0.00 (0.00-0.00) |
| Alcohol use disorder | Mental disorders | Autism spectrum disorders | 0.00 (0.00-0.00) |
| Alcohol use disorder | Mental disorders | ADHD | 0.00 (0.00-0.00) |
| Alcohol use disorder | Mental disorders | Conduct disorder | 0.00 (0.00-0.00) |
| Alcohol use disorder | Circulatory diseases | Hypertension | 0.01 (0.01-0.02) |
| Alcohol use disorder | Circulatory diseases | Dyslipidemia | 0.00 (0.00-0.00) |
| Alcohol use disorder | Circulatory diseases | Ischemic heart disease | 0.01 (0.00-0.01) |
| Alcohol use disorder | Circulatory diseases | Atrial fibrillation | 0.00 (0.00-0.01) |
| Alcohol use disorder | Circulatory diseases | Heart failure | 0.00 (0.00-0.00) |
| Alcohol use disorder | Circulatory diseases | Peripheral artery occlusive disease | 0.00 (0.00-0.00) |
| Alcohol use disorder | Circulatory diseases | Stroke | 0.00 (0.00-0.01) |
| Alcohol use disorder | Endocrine diseases | Diabetes | 0.00 (0.00-0.01) |
| Alcohol use disorder | Endocrine diseases | Thyroid disorder | 0.00 (0.00-0.00) |
| Alcohol use disorder | Endocrine diseases | Gout | 0.00 (0.00-0.00) |
| Alcohol use disorder | Pulmonary diseases | Asthma | 0.00 (0.00-0.00) |
| Alcohol use disorder | Pulmonary diseases | Chronic obstructive pulmonary disease | 0.02 (0.01-0.02) |
| Alcohol use disorder | Pulmonary diseases | Allergy | 0.03 (0.02-0.04) |
| Alcohol use disorder | Gastrointestinal diseases | Ulcer/chronic gastritis | 0.00 (0.00-0.01) |
| Alcohol use disorder | Gastrointestinal diseases | Chronic liver disease | 0.01 (0.01-0.01) |
| Alcohol use disorder | Gastrointestinal diseases | Inflammatory bowel disease | 0.00 (0.00-0.00) |
| Alcohol use disorder | Gastrointestinal diseases | Diverticular disease of intestine | 0.00 (0.00-0.00) |
| Alcohol use disorder | Urogenital diseases | Chronic kidney disease | 0.00 (0.00-0.00) |
| Alcohol use disorder | Urogenital diseases | Prostate disorders | 0.00 (0.00-0.00) |
| Alcohol use disorder | Musculoskeletal diseases | Connective | 0.00 (0.00-0.00) |
| Alcohol use disorder | Musculoskeletal diseases | Osteoporosis | 0.00 (0.00-0.00) |
| Alcohol use disorder | Musculoskeletal diseases | Painful conditions | 0.05 (0.04-0.06) |
| Alcohol use disorder | Hematological diseases | HIV/AIDS | 0.00 (0.00-0.00) |
| Alcohol use disorder | Hematological diseases | Anemia | 0.00 (0.00-0.00) |
| Alcohol use disorder | Cancers | Cancer | 0.00 (0.00-0.00) |
| Alcohol use disorder | Neurological diseases | Vision problems | 0.00 (0.00-0.00) |
| Alcohol use disorder | Neurological diseases | Hearing problems | 0.00 (0.00-0.00) |
| Alcohol use disorder | Neurological diseases | Migraine | 0.00 (0.00-0.00) |
| Alcohol use disorder | Neurological diseases | Epilepsy | 0.01 (0.01-0.01) |
| Alcohol use disorder | Neurological diseases | Parkinson’s disease | 0.00 (0.00-0.00) |
| Alcohol use disorder | Neurological diseases | Multiple sclerosis | 0.00 (0.00-0.00) |
| Alcohol use disorder | Neurological diseases | Neuropathies | 0.00 (0.00-0.00) |
| Opioid use disorder | Index disorder | Opioid use disorder | 0.38 (0.30-0.45) |
| Opioid use disorder | Mental disorders | Alcohol use disorder | 0.02 (0.01-0.02) |
| Opioid use disorder | Mental disorders | Cannabis use disorder | 0.01 (0.00-0.01) |
| Opioid use disorder | Mental disorders | Cocaine use disorder | 0.01 (0.01-0.01) |
| Opioid use disorder | Mental disorders | Amphetamine use disorder | 0.01 (0.01-0.01) |
| Opioid use disorder | Mental disorders | Other drug use disorders | 0.00 (0.00-0.00) |
| Opioid use disorder | Mental disorders | Schizophrenia | 0.09 (0.08-0.11) |
| Opioid use disorder | Mental disorders | Bipolar disorder | 0.01 (0.00-0.01) |
| Opioid use disorder | Mental disorders | Dysthymia | 0.00 (0.00-0.00) |
| Opioid use disorder | Mental disorders | Major depressive disorder | 0.01 (0.00-0.01) |
| Opioid use disorder | Mental disorders | Anxiety disorders | 0.01 (0.01-0.01) |
| Opioid use disorder | Mental disorders | Anorexia | 0.00 (0.00-0.00) |
| Opioid use disorder | Mental disorders | Bulimia | 0.00 (0.00-0.00) |
| Opioid use disorder | Mental disorders | Personality disorders | 0.03 (0.02-0.04) |
| Opioid use disorder | Mental disorders | Intellectual disability | 0.00 (0.00-0.00) |
| Opioid use disorder | Mental disorders | Autism spectrum disorders | 0.00 (0.00-0.00) |
| Opioid use disorder | Mental disorders | ADHD | 0.00 (0.00-0.00) |
| Opioid use disorder | Mental disorders | Conduct disorder | 0.00 (0.00-0.00) |
| Opioid use disorder | Circulatory diseases | Hypertension | 0.01 (0.00-0.01) |
| Opioid use disorder | Circulatory diseases | Dyslipidemia | 0.00 (0.00-0.00) |
| Opioid use disorder | Circulatory diseases | Ischemic heart disease | 0.00 (0.00-0.00) |
| Opioid use disorder | Circulatory diseases | Atrial fibrillation | 0.00 (0.00-0.00) |
| Opioid use disorder | Circulatory diseases | Heart failure | 0.00 (0.00-0.00) |
| Opioid use disorder | Circulatory diseases | Peripheral artery occlusive disease | 0.00 (0.00-0.00) |
| Opioid use disorder | Circulatory diseases | Stroke | 0.00 (0.00-0.00) |
| Opioid use disorder | Endocrine diseases | Diabetes | 0.00 (0.00-0.00) |
| Opioid use disorder | Endocrine diseases | Thyroid disorder | 0.00 (0.00-0.00) |
| Opioid use disorder | Endocrine diseases | Gout | 0.00 (0.00-0.00) |
| Opioid use disorder | Pulmonary diseases | Asthma | 0.01 (0.00-0.01) |
| Opioid use disorder | Pulmonary diseases | Chronic obstructive pulmonary disease | 0.01 (0.01-0.02) |
| Opioid use disorder | Pulmonary diseases | Allergy | 0.02 (0.02-0.03) |
| Opioid use disorder | Gastrointestinal diseases | Ulcer/chronic gastritis | 0.00 (0.00-0.00) |
| Opioid use disorder | Gastrointestinal diseases | Chronic liver disease | 0.03 (0.02-0.04) |
| Opioid use disorder | Gastrointestinal diseases | Inflammatory bowel disease | 0.00 (0.00-0.00) |
| Opioid use disorder | Gastrointestinal diseases | Diverticular disease of intestine | 0.00 (0.00-0.00) |
| Opioid use disorder | Urogenital diseases | Chronic kidney disease | 0.00 (0.00-0.00) |
| Opioid use disorder | Urogenital diseases | Prostate disorders | 0.00 (0.00-0.00) |
| Opioid use disorder | Musculoskeletal diseases | Connective | 0.00 (0.00-0.00) |
| Opioid use disorder | Musculoskeletal diseases | Osteoporosis | 0.00 (0.00-0.00) |
| Opioid use disorder | Musculoskeletal diseases | Painful conditions | 0.06 (0.04-0.07) |
| Opioid use disorder | Hematological diseases | HIV/AIDS | 0.00 (0.00-0.00) |
| Opioid use disorder | Hematological diseases | Anemia | 0.00 (0.00-0.00) |
| Opioid use disorder | Cancers | Cancer | 0.00 (0.00-0.00) |
| Opioid use disorder | Neurological diseases | Vision problems | 0.00 (0.00-0.00) |
| Opioid use disorder | Neurological diseases | Hearing problems | 0.00 (0.00-0.00) |
| Opioid use disorder | Neurological diseases | Migraine | 0.00 (0.00-0.00) |
| Opioid use disorder | Neurological diseases | Epilepsy | 0.01 (0.01-0.01) |
| Opioid use disorder | Neurological diseases | Parkinson’s disease | 0.00 (0.00-0.00) |
| Opioid use disorder | Neurological diseases | Multiple sclerosis | 0.00 (0.00-0.00) |
| Opioid use disorder | Neurological diseases | Neuropathies | 0.00 (0.00-0.00) |
| Cannabis use disorder | Index disorder | Cannabis use disorder | 0.06 (0.04-0.08) |
| Cannabis use disorder | Mental disorders | Alcohol use disorder | 0.03 (0.02-0.04) |
| Cannabis use disorder | Mental disorders | Opioid use disorder | 0.03 (0.02-0.03) |
| Cannabis use disorder | Mental disorders | Cocaine use disorder | 0.01 (0.01-0.01) |
| Cannabis use disorder | Mental disorders | Amphetamine use disorder | 0.01 (0.01-0.01) |
| Cannabis use disorder | Mental disorders | Other drug use disorders | 0.00 (0.00-0.00) |
| Cannabis use disorder | Mental disorders | Schizophrenia | 0.18 (0.15-0.20) |
| Cannabis use disorder | Mental disorders | Bipolar disorder | 0.01 (0.01-0.01) |
| Cannabis use disorder | Mental disorders | Dysthymia | 0.00 (0.00-0.00) |
| Cannabis use disorder | Mental disorders | Major depressive disorder | 0.01 (0.01-0.01) |
| Cannabis use disorder | Mental disorders | Anxiety disorders | 0.01 (0.01-0.02) |
| Cannabis use disorder | Mental disorders | Anorexia | 0.00 (0.00-0.00) |
| Cannabis use disorder | Mental disorders | Bulimia | 0.00 (0.00-0.00) |
| Cannabis use disorder | Mental disorders | Personality disorders | 0.04 (0.03-0.05) |
| Cannabis use disorder | Mental disorders | Intellectual disability | 0.00 (0.00-0.00) |
| Cannabis use disorder | Mental disorders | Autism spectrum disorders | 0.00 (0.00-0.00) |
| Cannabis use disorder | Mental disorders | ADHD | 0.00 (0.00-0.01) |
| Cannabis use disorder | Mental disorders | Conduct disorder | 0.00 (0.00-0.00) |
| Cannabis use disorder | Circulatory diseases | Hypertension | 0.00 (0.00-0.00) |
| Cannabis use disorder | Circulatory diseases | Dyslipidemia | 0.00 (0.00-0.00) |
| Cannabis use disorder | Circulatory diseases | Ischemic heart disease | 0.00 (0.00-0.00) |
| Cannabis use disorder | Circulatory diseases | Atrial fibrillation | 0.00 (0.00-0.00) |
| Cannabis use disorder | Circulatory diseases | Heart failure | 0.00 (0.00-0.00) |
| Cannabis use disorder | Circulatory diseases | Peripheral artery occlusive disease | 0.00 (0.00-0.00) |
| Cannabis use disorder | Circulatory diseases | Stroke | 0.00 (0.00-0.00) |
| Cannabis use disorder | Endocrine diseases | Diabetes | 0.00 (0.00-0.00) |
| Cannabis use disorder | Endocrine diseases | Thyroid disorder | 0.00 (0.00-0.00) |
| Cannabis use disorder | Endocrine diseases | Gout | 0.00 (0.00-0.00) |
| Cannabis use disorder | Pulmonary diseases | Asthma | 0.01 (0.01-0.01) |
| Cannabis use disorder | Pulmonary diseases | Chronic obstructive pulmonary disease | 0.01 (0.00-0.01) |
| Cannabis use disorder | Pulmonary diseases | Allergy | 0.02 (0.02-0.03) |
| Cannabis use disorder | Gastrointestinal diseases | Ulcer/chronic gastritis | 0.00 (0.00-0.00) |
| Cannabis use disorder | Gastrointestinal diseases | Chronic liver disease | 0.01 (0.00-0.01) |
| Cannabis use disorder | Gastrointestinal diseases | Inflammatory bowel disease | 0.00 (0.00-0.00) |
| Cannabis use disorder | Gastrointestinal diseases | Diverticular disease of intestine | 0.00 (0.00-0.00) |
| Cannabis use disorder | Urogenital diseases | Chronic kidney disease | 0.00 (0.00-0.00) |
| Cannabis use disorder | Urogenital diseases | Prostate disorders | 0.00 (0.00-0.00) |
| Cannabis use disorder | Musculoskeletal diseases | Connective | 0.00 (0.00-0.00) |
| Cannabis use disorder | Musculoskeletal diseases | Osteoporosis | 0.00 (0.00-0.00) |
| Cannabis use disorder | Musculoskeletal diseases | Painful conditions | 0.03 (0.02-0.04) |
| Cannabis use disorder | Hematological diseases | HIV/AIDS | 0.00 (0.00-0.00) |
| Cannabis use disorder | Hematological diseases | Anemia | 0.00 (0.00-0.00) |
| Cannabis use disorder | Cancers | Cancer | 0.00 (0.00-0.00) |
| Cannabis use disorder | Neurological diseases | Vision problems | 0.00 (0.00-0.00) |
| Cannabis use disorder | Neurological diseases | Hearing problems | 0.00 (0.00-0.00) |
| Cannabis use disorder | Neurological diseases | Migraine | 0.00 (0.00-0.00) |
| Cannabis use disorder | Neurological diseases | Epilepsy | 0.01 (0.00-0.01) |
| Cannabis use disorder | Neurological diseases | Parkinson’s disease | 0.00 (0.00-0.00) |
| Cannabis use disorder | Neurological diseases | Multiple sclerosis | 0.00 (0.00-0.00) |
| Cannabis use disorder | Neurological diseases | Neuropathies | 0.00 (0.00-0.00) |
| Cocaine use disorder | Index disorder | Cocaine use disorder | 0.21 (0.16-0.26) |
| Cocaine use disorder | Mental disorders | Alcohol use disorder | 0.03 (0.02-0.04) |
| Cocaine use disorder | Mental disorders | Opioid use disorder | 0.08 (0.06-0.09) |
| Cocaine use disorder | Mental disorders | Cannabis use disorder | 0.01 (0.01-0.02) |
| Cocaine use disorder | Mental disorders | Amphetamine use disorder | 0.03 (0.02-0.04) |
| Cocaine use disorder | Mental disorders | Other drug use disorders | 0.00 (0.00-0.00) |
| Cocaine use disorder | Mental disorders | Schizophrenia | 0.11 (0.09-0.13) |
| Cocaine use disorder | Mental disorders | Bipolar disorder | 0.01 (0.01-0.01) |
| Cocaine use disorder | Mental disorders | Dysthymia | 0.00 (0.00-0.00) |
| Cocaine use disorder | Mental disorders | Major depressive disorder | 0.01 (0.00-0.01) |
| Cocaine use disorder | Mental disorders | Anxiety disorders | 0.01 (0.01-0.02) |
| Cocaine use disorder | Mental disorders | Anorexia | 0.00 (0.00-0.00) |
| Cocaine use disorder | Mental disorders | Bulimia | 0.00 (0.00-0.00) |
| Cocaine use disorder | Mental disorders | Personality disorders | 0.04 (0.03-0.05) |
| Cocaine use disorder | Mental disorders | Intellectual disability | 0.00 (0.00-0.00) |
| Cocaine use disorder | Mental disorders | Autism spectrum disorders | 0.00 (0.00-0.00) |
| Cocaine use disorder | Mental disorders | ADHD | 0.00 (0.00-0.00) |
| Cocaine use disorder | Mental disorders | Conduct disorder | 0.00 (0.00-0.00) |
| Cocaine use disorder | Circulatory diseases | Hypertension | 0.00 (0.00-0.01) |
| Cocaine use disorder | Circulatory diseases | Dyslipidemia | 0.00 (0.00-0.00) |
| Cocaine use disorder | Circulatory diseases | Ischemic heart disease | 0.00 (0.00-0.00) |
| Cocaine use disorder | Circulatory diseases | Atrial fibrillation | 0.00 (0.00-0.00) |
| Cocaine use disorder | Circulatory diseases | Heart failure | 0.00 (0.00-0.00) |
| Cocaine use disorder | Circulatory diseases | Peripheral artery occlusive disease | 0.00 (0.00-0.00) |
| Cocaine use disorder | Circulatory diseases | Stroke | 0.00 (0.00-0.00) |
| Cocaine use disorder | Endocrine diseases | Diabetes | 0.00 (0.00-0.00) |
| Cocaine use disorder | Endocrine diseases | Thyroid disorder | 0.00 (0.00-0.00) |
| Cocaine use disorder | Endocrine diseases | Gout | 0.00 (0.00-0.00) |
| Cocaine use disorder | Pulmonary diseases | Asthma | 0.01 (0.01-0.01) |
| Cocaine use disorder | Pulmonary diseases | Chronic obstructive pulmonary disease | 0.01 (0.00-0.01) |
| Cocaine use disorder | Pulmonary diseases | Allergy | 0.02 (0.02-0.03) |
| Cocaine use disorder | Gastrointestinal diseases | Ulcer/chronic gastritis | 0.00 (0.00-0.00) |
| Cocaine use disorder | Gastrointestinal diseases | Chronic liver disease | 0.02 (0.01-0.02) |
| Cocaine use disorder | Gastrointestinal diseases | Inflammatory bowel disease | 0.00 (0.00-0.00) |
| Cocaine use disorder | Gastrointestinal diseases | Diverticular disease of intestine | 0.00 (0.00-0.00) |
| Cocaine use disorder | Urogenital diseases | Chronic kidney disease | 0.00 (0.00-0.00) |
| Cocaine use disorder | Urogenital diseases | Prostate disorders | 0.00 (0.00-0.00) |
| Cocaine use disorder | Musculoskeletal diseases | Connective | 0.00 (0.00-0.00) |
| Cocaine use disorder | Musculoskeletal diseases | Osteoporosis | 0.00 (0.00-0.00) |
| Cocaine use disorder | Musculoskeletal diseases | Painful conditions | 0.03 (0.02-0.04) |
| Cocaine use disorder | Hematological diseases | HIV/AIDS | 0.00 (0.00-0.00) |
| Cocaine use disorder | Hematological diseases | Anemia | 0.00 (0.00-0.00) |
| Cocaine use disorder | Cancers | Cancer | 0.00 (0.00-0.00) |
| Cocaine use disorder | Neurological diseases | Hearing problems | 0.00 (0.00-0.00) |
| Cocaine use disorder | Neurological diseases | Migraine | 0.00 (0.00-0.00) |
| Cocaine use disorder | Neurological diseases | Epilepsy | 0.01 (0.00-0.01) |
| Cocaine use disorder | Neurological diseases | Neuropathies | 0.00 (0.00-0.00) |
| Cocaine use disorder | Neurological diseases | Other | 0.00 (0.00-0.00) |
| Amphetamine use disorder | Index disorder | Amphetamine use disorder | 0.23 (0.17-0.28) |
| Amphetamine use disorder | Mental disorders | Alcohol use disorder | 0.03 (0.02-0.04) |
| Amphetamine use disorder | Mental disorders | Opioid use disorder | 0.05 (0.03-0.05) |
| Amphetamine use disorder | Mental disorders | Cannabis use disorder | 0.02 (0.01-0.02) |
| Amphetamine use disorder | Mental disorders | Cocaine use disorder | 0.03 (0.02-0.04) |
| Amphetamine use disorder | Mental disorders | Other drug use disorders | 0.00 (0.00-0.00) |
| Amphetamine use disorder | Mental disorders | Schizophrenia | 0.14 (0.11-0.16) |
| Amphetamine use disorder | Mental disorders | Bipolar disorder | 0.01 (0.01-0.01) |
| Amphetamine use disorder | Mental disorders | Dysthymia | 0.00 (0.00-0.00) |
| Amphetamine use disorder | Mental disorders | Major depressive disorder | 0.01 (0.00-0.01) |
| Amphetamine use disorder | Mental disorders | Anxiety disorders | 0.01 (0.01-0.02) |
| Amphetamine use disorder | Mental disorders | Anorexia | 0.00 (0.00-0.00) |
| Amphetamine use disorder | Mental disorders | Bulimia | 0.00 (0.00-0.00) |
| Amphetamine use disorder | Mental disorders | Personality disorders | 0.03 (0.02-0.04) |
| Amphetamine use disorder | Mental disorders | Intellectual disability | 0.00 (0.00-0.00) |
| Amphetamine use disorder | Mental disorders | Autism spectrum disorders | 0.00 (0.00-0.00) |
| Amphetamine use disorder | Mental disorders | ADHD | 0.01 (0.00-0.01) |
| Amphetamine use disorder | Mental disorders | Conduct disorder | 0.00 (0.00-0.00) |
| Amphetamine use disorder | Circulatory diseases | Hypertension | 0.00 (0.00-0.00) |
| Amphetamine use disorder | Circulatory diseases | Dyslipidemia | 0.00 (0.00-0.00) |
| Amphetamine use disorder | Circulatory diseases | Ischemic heart disease | 0.00 (0.00-0.00) |
| Amphetamine use disorder | Circulatory diseases | Atrial fibrillation | 0.00 (0.00-0.00) |
| Amphetamine use disorder | Circulatory diseases | Heart failure | 0.00 (0.00-0.00) |
| Amphetamine use disorder | Circulatory diseases | Peripheral artery occlusive disease | 0.00 (0.00-0.00) |
| Amphetamine use disorder | Circulatory diseases | Stroke | 0.00 (0.00-0.00) |
| Amphetamine use disorder | Endocrine diseases | Diabetes | 0.00 (0.00-0.00) |
| Amphetamine use disorder | Endocrine diseases | Thyroid disorder | 0.00 (0.00-0.00) |
| Amphetamine use disorder | Endocrine diseases | Gout | 0.00 (0.00-0.00) |
| Amphetamine use disorder | Pulmonary diseases | Asthma | 0.01 (0.01-0.01) |
| Amphetamine use disorder | Pulmonary diseases | Chronic obstructive pulmonary disease | 0.00 (0.00-0.01) |
| Amphetamine use disorder | Pulmonary diseases | Allergy | 0.02 (0.02-0.03) |
| Amphetamine use disorder | Gastrointestinal diseases | Ulcer/chronic gastritis | 0.00 (0.00-0.00) |
| Amphetamine use disorder | Gastrointestinal diseases | Chronic liver disease | 0.01 (0.01-0.01) |
| Amphetamine use disorder | Gastrointestinal diseases | Inflammatory bowel disease | 0.00 (0.00-0.00) |
| Amphetamine use disorder | Gastrointestinal diseases | Diverticular disease of intestine | 0.00 (0.00-0.00) |
| Amphetamine use disorder | Urogenital diseases | Chronic kidney disease | 0.00 (0.00-0.00) |
| Amphetamine use disorder | Urogenital diseases | Prostate disorders | 0.00 (0.00-0.00) |
| Amphetamine use disorder | Musculoskeletal diseases | Connective | 0.00 (0.00-0.00) |
| Amphetamine use disorder | Musculoskeletal diseases | Osteoporosis | 0.00 (0.00-0.00) |
| Amphetamine use disorder | Musculoskeletal diseases | Painful conditions | 0.03 (0.02-0.04) |
| Amphetamine use disorder | Hematological diseases | HIV/AIDS | 0.00 (0.00-0.00) |
| Amphetamine use disorder | Hematological diseases | Anemia | 0.00 (0.00-0.00) |
| Amphetamine use disorder | Cancers | Cancer | 0.00 (0.00-0.00) |
| Amphetamine use disorder | Neurological diseases | Vision problems | 0.00 (0.00-0.00) |
| Amphetamine use disorder | Neurological diseases | Hearing problems | 0.00 (0.00-0.00) |
| Amphetamine use disorder | Neurological diseases | Migraine | 0.00 (0.00-0.00) |
| Amphetamine use disorder | Neurological diseases | Epilepsy | 0.01 (0.00-0.01) |
| Amphetamine use disorder | Neurological diseases | Parkinson’s disease | 0.00 (0.00-0.00) |
| Amphetamine use disorder | Neurological diseases | Multiple sclerosis | 0.00 (0.00-0.00) |
| Amphetamine use disorder | Neurological diseases | Neuropathies | 0.00 (0.00-0.00) |
| Other drug use disorders | Index disorder | Other drug use disorders | 0.08 (0.06-0.11) |
| Other drug use disorders | Mental disorders | Alcohol use disorder | 0.05 (0.04-0.07) |
| Other drug use disorders | Mental disorders | Opioid use disorder | 0.06 (0.05-0.08) |
| Other drug use disorders | Mental disorders | Cannabis use disorder | 0.01 (0.00-0.01) |
| Other drug use disorders | Mental disorders | Cocaine use disorder | 0.01 (0.01-0.01) |
| Other drug use disorders | Mental disorders | Amphetamine use disorder | 0.01 (0.01-0.01) |
| Other drug use disorders | Mental disorders | Schizophrenia | 0.07 (0.06-0.08) |
| Other drug use disorders | Mental disorders | Bipolar disorder | 0.02 (0.01-0.02) |
| Other drug use disorders | Mental disorders | Dysthymia | 0.00 (0.00-0.00) |
| Other drug use disorders | Mental disorders | Major depressive disorder | 0.04 (0.02-0.05) |
| Other drug use disorders | Mental disorders | Anxiety disorders | 0.03 (0.02-0.04) |
| Other drug use disorders | Mental disorders | Anorexia | 0.00 (0.00-0.00) |
| Other drug use disorders | Mental disorders | Bulimia | 0.00 (0.00-0.00) |
| Other drug use disorders | Mental disorders | Personality disorders | 0.04 (0.03-0.05) |
| Other drug use disorders | Mental disorders | Intellectual disability | 0.00 (0.00-0.00) |
| Other drug use disorders | Mental disorders | Autism spectrum disorders | 0.00 (0.00-0.00) |
| Other drug use disorders | Mental disorders | ADHD | 0.00 (0.00-0.00) |
| Other drug use disorders | Mental disorders | Conduct disorder | 0.00 (0.00-0.00) |
| Other drug use disorders | Circulatory diseases | Hypertension | 0.01 (0.01-0.02) |
| Other drug use disorders | Circulatory diseases | Dyslipidemia | 0.00 (0.00-0.00) |
| Other drug use disorders | Circulatory diseases | Ischemic heart disease | 0.01 (0.00-0.01) |
| Other drug use disorders | Circulatory diseases | Atrial fibrillation | 0.00 (0.00-0.00) |
| Other drug use disorders | Circulatory diseases | Heart failure | 0.00 (0.00-0.00) |
| Other drug use disorders | Circulatory diseases | Peripheral artery occlusive disease | 0.00 (0.00-0.00) |
| Other drug use disorders | Circulatory diseases | Stroke | 0.00 (0.00-0.01) |
| Other drug use disorders | Endocrine diseases | Diabetes | 0.00 (0.00-0.00) |
| Other drug use disorders | Endocrine diseases | Thyroid disorder | 0.00 (0.00-0.00) |
| Other drug use disorders | Endocrine diseases | Gout | 0.00 (0.00-0.00) |
| Other drug use disorders | Pulmonary diseases | Asthma | 0.00 (0.00-0.01) |
| Other drug use disorders | Pulmonary diseases | Chronic obstructive pulmonary disease | 0.02 (0.01-0.02) |
| Other drug use disorders | Pulmonary diseases | Allergy | 0.04 (0.03-0.05) |
| Other drug use disorders | Gastrointestinal diseases | Ulcer/chronic gastritis | 0.00 (0.00-0.00) |
| Other drug use disorders | Gastrointestinal diseases | Chronic liver disease | 0.01 (0.01-0.01) |
| Other drug use disorders | Gastrointestinal diseases | Inflammatory bowel disease | 0.00 (0.00-0.00) |
| Other drug use disorders | Gastrointestinal diseases | Diverticular disease of intestine | 0.00 (0.00-0.00) |
| Other drug use disorders | Urogenital diseases | Chronic kidney disease | 0.00 (0.00-0.00) |
| Other drug use disorders | Urogenital diseases | Prostate disorders | 0.00 (0.00-0.00) |
| Other drug use disorders | Musculoskeletal diseases | Connective | 0.00 (0.00-0.00) |
| Other drug use disorders | Musculoskeletal diseases | Osteoporosis | 0.00 (0.00-0.00) |
| Other drug use disorders | Musculoskeletal diseases | Painful conditions | 0.07 (0.05-0.08) |
| Other drug use disorders | Hematological diseases | HIV/AIDS | 0.00 (0.00-0.00) |
| Other drug use disorders | Hematological diseases | Anemia | 0.00 (0.00-0.00) |
| Other drug use disorders | Cancers | Cancer | 0.00 (0.00-0.00) |
| Other drug use disorders | Neurological diseases | Vision problems | 0.00 (0.00-0.00) |
| Other drug use disorders | Neurological diseases | Hearing problems | 0.00 (0.00-0.00) |
| Other drug use disorders | Neurological diseases | Migraine | 0.00 (0.00-0.01) |
| Other drug use disorders | Neurological diseases | Epilepsy | 0.01 (0.01-0.01) |
| Other drug use disorders | Neurological diseases | Parkinson’s disease | 0.00 (0.00-0.00) |
| Other drug use disorders | Neurological diseases | Multiple sclerosis | 0.00 (0.00-0.00) |
| Other drug use disorders | Neurological diseases | Neuropathies | 0.00 (0.00-0.00) |
| Schizophrenia | Index disorder | Schizophrenia | 0.59 (0.50-0.68) |
| Schizophrenia | Mental disorders | Alcohol use disorder | 0.01 (0.01-0.01) |
| Schizophrenia | Mental disorders | Opioid use disorder | 0.01 (0.01-0.01) |
| Schizophrenia | Mental disorders | Cannabis use disorder | 0.00 (0.00-0.00) |
| Schizophrenia | Mental disorders | Cocaine use disorder | 0.00 (0.00-0.00) |
| Schizophrenia | Mental disorders | Amphetamine use disorder | 0.00 (0.00-0.00) |
| Schizophrenia | Mental disorders | Other drug use disorders | 0.00 (0.00-0.00) |
| Schizophrenia | Mental disorders | Bipolar disorder | 0.01 (0.00-0.01) |
| Schizophrenia | Mental disorders | Dysthymia | 0.00 (0.00-0.00) |
| Schizophrenia | Mental disorders | Major depressive disorder | 0.00 (0.00-0.00) |
| Schizophrenia | Mental disorders | Anxiety disorders | 0.01 (0.01-0.01) |
| Schizophrenia | Mental disorders | Anorexia | 0.00 (0.00-0.00) |
| Schizophrenia | Mental disorders | Bulimia | 0.00 (0.00-0.00) |
| Schizophrenia | Mental disorders | Personality disorders | 0.02 (0.01-0.03) |
| Schizophrenia | Mental disorders | Intellectual disability | 0.00 (0.00-0.00) |
| Schizophrenia | Mental disorders | Autism spectrum disorders | 0.00 (0.00-0.00) |
| Schizophrenia | Mental disorders | ADHD | 0.00 (0.00-0.00) |
| Schizophrenia | Mental disorders | Conduct disorder | 0.00 (0.00-0.00) |
| Schizophrenia | Circulatory diseases | Hypertension | 0.01 (0.01-0.01) |
| Schizophrenia | Circulatory diseases | Dyslipidemia | 0.00 (0.00-0.00) |
| Schizophrenia | Circulatory diseases | Ischemic heart disease | 0.00 (0.00-0.00) |
| Schizophrenia | Circulatory diseases | Atrial fibrillation | 0.00 (0.00-0.00) |
| Schizophrenia | Circulatory diseases | Heart failure | 0.00 (0.00-0.00) |
| Schizophrenia | Circulatory diseases | Peripheral artery occlusive disease | 0.00 (0.00-0.00) |
| Schizophrenia | Circulatory diseases | Stroke | 0.00 (0.00-0.00) |
| Schizophrenia | Endocrine diseases | Diabetes | 0.00 (0.00-0.01) |
| Schizophrenia | Endocrine diseases | Thyroid disorder | 0.00 (0.00-0.00) |
| Schizophrenia | Endocrine diseases | Gout | 0.00 (0.00-0.00) |
| Schizophrenia | Pulmonary diseases | Asthma | 0.00 (0.00-0.01) |
| Schizophrenia | Pulmonary diseases | Chronic obstructive pulmonary disease | 0.01 (0.01-0.01) |
| Schizophrenia | Pulmonary diseases | Allergy | 0.02 (0.02-0.03) |
| Schizophrenia | Gastrointestinal diseases | Ulcer/chronic gastritis | 0.00 (0.00-0.00) |
| Schizophrenia | Gastrointestinal diseases | Chronic liver disease | 0.00 (0.00-0.00) |
| Schizophrenia | Gastrointestinal diseases | Inflammatory bowel disease | 0.00 (0.00-0.00) |
| Schizophrenia | Gastrointestinal diseases | Diverticular disease of intestine | 0.00 (0.00-0.00) |
| Schizophrenia | Urogenital diseases | Chronic kidney disease | 0.00 (0.00-0.00) |
| Schizophrenia | Urogenital diseases | Prostate disorders | 0.00 (0.00-0.00) |
| Schizophrenia | Musculoskeletal diseases | Connective | 0.00 (0.00-0.00) |
| Schizophrenia | Musculoskeletal diseases | Osteoporosis | 0.00 (0.00-0.00) |
| Schizophrenia | Musculoskeletal diseases | Painful conditions | 0.03 (0.02-0.04) |
| Schizophrenia | Hematological diseases | HIV/AIDS | 0.00 (0.00-0.00) |
| Schizophrenia | Hematological diseases | Anemia | 0.00 (0.00-0.00) |
| Schizophrenia | Cancers | Cancer | 0.00 (0.00-0.00) |
| Schizophrenia | Neurological diseases | Vision problems | 0.00 (0.00-0.00) |
| Schizophrenia | Neurological diseases | Hearing problems | 0.00 (0.00-0.00) |
| Schizophrenia | Neurological diseases | Migraine | 0.00 (0.00-0.00) |
| Schizophrenia | Neurological diseases | Epilepsy | 0.01 (0.00-0.01) |
| Schizophrenia | Neurological diseases | Parkinson’s disease | 0.00 (0.00-0.00) |
| Schizophrenia | Neurological diseases | Multiple sclerosis | 0.00 (0.00-0.00) |
| Schizophrenia | Neurological diseases | Neuropathies | 0.00 (0.00-0.00) |
| Bipolar disorder | Index disorder | Bipolar disorder | 0.18 (0.14-0.23) |
| Bipolar disorder | Mental disorders | Alcohol use disorder | 0.01 (0.01-0.02) |
| Bipolar disorder | Mental disorders | Opioid use disorder | 0.00 (0.00-0.00) |
| Bipolar disorder | Mental disorders | Cannabis use disorder | 0.00 (0.00-0.00) |
| Bipolar disorder | Mental disorders | Cocaine use disorder | 0.00 (0.00-0.00) |
| Bipolar disorder | Mental disorders | Amphetamine use disorder | 0.00 (0.00-0.00) |
| Bipolar disorder | Mental disorders | Other drug use disorders | 0.00 (0.00-0.00) |
| Bipolar disorder | Mental disorders | Schizophrenia | 0.04 (0.03-0.04) |
| Bipolar disorder | Mental disorders | Dysthymia | 0.00 (0.00-0.00) |
| Bipolar disorder | Mental disorders | Major depressive disorder | 0.01 (0.01-0.01) |
| Bipolar disorder | Mental disorders | Anxiety disorders | 0.01 (0.01-0.01) |
| Bipolar disorder | Mental disorders | Anorexia | 0.00 (0.00-0.00) |
| Bipolar disorder | Mental disorders | Bulimia | 0.00 (0.00-0.00) |
| Bipolar disorder | Mental disorders | Personality disorders | 0.02 (0.02-0.03) |
| Bipolar disorder | Mental disorders | Intellectual disability | 0.00 (0.00-0.00) |
| Bipolar disorder | Mental disorders | Autism spectrum disorders | 0.00 (0.00-0.00) |
| Bipolar disorder | Mental disorders | ADHD | 0.00 (0.00-0.00) |
| Bipolar disorder | Mental disorders | Conduct disorder | 0.00 (0.00-0.00) |
| Bipolar disorder | Circulatory diseases | Hypertension | 0.01 (0.01-0.02) |
| Bipolar disorder | Circulatory diseases | Dyslipidemia | 0.00 (0.00-0.01) |
| Bipolar disorder | Circulatory diseases | Ischemic heart disease | 0.01 (0.00-0.01) |
| Bipolar disorder | Circulatory diseases | Atrial fibrillation | 0.00 (0.00-0.01) |
| Bipolar disorder | Circulatory diseases | Heart failure | 0.00 (0.00-0.00) |
| Bipolar disorder | Circulatory diseases | Peripheral artery occlusive disease | 0.00 (0.00-0.00) |
| Bipolar disorder | Circulatory diseases | Stroke | 0.00 (0.00-0.01) |
| Bipolar disorder | Endocrine diseases | Diabetes | 0.00 (0.00-0.01) |
| Bipolar disorder | Endocrine diseases | Thyroid disorder | 0.01 (0.00-0.01) |
| Bipolar disorder | Endocrine diseases | Gout | 0.00 (0.00-0.00) |
| Bipolar disorder | Pulmonary diseases | Asthma | 0.00 (0.00-0.01) |
| Bipolar disorder | Pulmonary diseases | Chronic obstructive pulmonary disease | 0.01 (0.01-0.02) |
| Bipolar disorder | Pulmonary diseases | Allergy | 0.03 (0.03-0.04) |
| Bipolar disorder | Gastrointestinal diseases | Ulcer/chronic gastritis | 0.00 (0.00-0.00) |
| Bipolar disorder | Gastrointestinal diseases | Chronic liver disease | 0.00 (0.00-0.00) |
| Bipolar disorder | Gastrointestinal diseases | Inflammatory bowel disease | 0.00 (0.00-0.00) |
| Bipolar disorder | Gastrointestinal diseases | Diverticular disease of intestine | 0.00 (0.00-0.00) |
| Bipolar disorder | Urogenital diseases | Chronic kidney disease | 0.00 (0.00-0.00) |
| Bipolar disorder | Urogenital diseases | Prostate disorders | 0.00 (0.00-0.00) |
| Bipolar disorder | Musculoskeletal diseases | Connective | 0.00 (0.00-0.00) |
| Bipolar disorder | Musculoskeletal diseases | Osteoporosis | 0.00 (0.00-0.00) |
| Bipolar disorder | Musculoskeletal diseases | Painful conditions | 0.05 (0.03-0.06) |
| Bipolar disorder | Hematological diseases | HIV/AIDS | 0.00 (0.00-0.00) |
| Bipolar disorder | Hematological diseases | Anemia | 0.00 (0.00-0.00) |
| Bipolar disorder | Cancers | Cancer | 0.00 (0.00-0.00) |
| Bipolar disorder | Neurological diseases | Vision problems | 0.00 (0.00-0.00) |
| Bipolar disorder | Neurological diseases | Hearing problems | 0.00 (0.00-0.00) |
| Bipolar disorder | Neurological diseases | Migraine | 0.00 (0.00-0.00) |
| Bipolar disorder | Neurological diseases | Epilepsy | 0.01 (0.00-0.01) |
| Bipolar disorder | Neurological diseases | Parkinson’s disease | 0.00 (0.00-0.00) |
| Bipolar disorder | Neurological diseases | Multiple sclerosis | 0.00 (0.00-0.00) |
| Bipolar disorder | Neurological diseases | Neuropathies | 0.00 (0.00-0.00) |
| Dysthymia | Index disorder | Dysthymia | 0.12 (0.09-0.16) |
| Dysthymia | Mental disorders | Alcohol use disorder | 0.01 (0.01-0.01) |
| Dysthymia | Mental disorders | Opioid use disorder | 0.00 (0.00-0.00) |
| Dysthymia | Mental disorders | Cannabis use disorder | 0.00 (0.00-0.00) |
| Dysthymia | Mental disorders | Amphetamine use disorder | 0.00 (0.00-0.00) |
| Dysthymia | Mental disorders | Other drug use disorders | 0.00 (0.00-0.00) |
| Dysthymia | Mental disorders | Schizophrenia | 0.01 (0.01-0.02) |
| Dysthymia | Mental disorders | Bipolar disorder | 0.01 (0.01-0.01) |
| Dysthymia | Mental disorders | Major depressive disorder | 0.02 (0.01-0.02) |
| Dysthymia | Mental disorders | Anxiety disorders | 0.02 (0.02-0.03) |
| Dysthymia | Mental disorders | Anorexia | 0.00 (0.00-0.00) |
| Dysthymia | Mental disorders | Bulimia | 0.00 (0.00-0.00) |
| Dysthymia | Mental disorders | Personality disorders | 0.04 (0.03-0.05) |
| Dysthymia | Mental disorders | Intellectual disability | 0.00 (0.00-0.00) |
| Dysthymia | Mental disorders | Autism spectrum disorders | 0.00 (0.00-0.00) |
| Dysthymia | Mental disorders | ADHD | 0.00 (0.00-0.00) |
| Dysthymia | Mental disorders | Other | 0.00 (0.00-0.00) |
| Dysthymia | Circulatory diseases | Hypertension | 0.01 (0.01-0.02) |
| Dysthymia | Circulatory diseases | Dyslipidemia | 0.00 (0.00-0.00) |
| Dysthymia | Circulatory diseases | Ischemic heart disease | 0.01 (0.00-0.01) |
| Dysthymia | Circulatory diseases | Atrial fibrillation | 0.00 (0.00-0.00) |
| Dysthymia | Circulatory diseases | Heart failure | 0.00 (0.00-0.00) |
| Dysthymia | Circulatory diseases | Peripheral artery occlusive disease | 0.00 (0.00-0.00) |
| Dysthymia | Circulatory diseases | Stroke | 0.00 (0.00-0.00) |
| Dysthymia | Endocrine diseases | Diabetes | 0.00 (0.00-0.01) |
| Dysthymia | Endocrine diseases | Thyroid disorder | 0.00 (0.00-0.01) |
| Dysthymia | Endocrine diseases | Gout | 0.00 (0.00-0.00) |
| Dysthymia | Pulmonary diseases | Asthma | 0.01 (0.00-0.01) |
| Dysthymia | Pulmonary diseases | Chronic obstructive pulmonary disease | 0.01 (0.01-0.02) |
| Dysthymia | Pulmonary diseases | Allergy | 0.04 (0.03-0.05) |
| Dysthymia | Gastrointestinal diseases | Ulcer/chronic gastritis | 0.00 (0.00-0.00) |
| Dysthymia | Gastrointestinal diseases | Chronic liver disease | 0.00 (0.00-0.00) |
| Dysthymia | Gastrointestinal diseases | Inflammatory bowel disease | 0.00 (0.00-0.00) |
| Dysthymia | Gastrointestinal diseases | Diverticular disease of intestine | 0.00 (0.00-0.00) |
| Dysthymia | Urogenital diseases | Chronic kidney disease | 0.00 (0.00-0.00) |
| Dysthymia | Urogenital diseases | Prostate disorders | 0.00 (0.00-0.00) |
| Dysthymia | Musculoskeletal diseases | Connective | 0.00 (0.00-0.00) |
| Dysthymia | Musculoskeletal diseases | Osteoporosis | 0.00 (0.00-0.00) |
| Dysthymia | Musculoskeletal diseases | Painful conditions | 0.05 (0.04-0.06) |
| Dysthymia | Hematological diseases | HIV/AIDS | 0.00 (0.00-0.00) |
| Dysthymia | Hematological diseases | Anemia | 0.00 (0.00-0.00) |
| Dysthymia | Cancers | Cancer | 0.00 (0.00-0.00) |
| Dysthymia | Neurological diseases | Vision problems | 0.00 (0.00-0.00) |
| Dysthymia | Neurological diseases | Hearing problems | 0.00 (0.00-0.00) |
| Dysthymia | Neurological diseases | Migraine | 0.00 (0.00-0.00) |
| Dysthymia | Neurological diseases | Epilepsy | 0.00 (0.00-0.01) |
| Dysthymia | Neurological diseases | Parkinson’s disease | 0.00 (0.00-0.00) |
| Dysthymia | Neurological diseases | Multiple sclerosis | 0.00 (0.00-0.00) |
| Dysthymia | Neurological diseases | Neuropathies | 0.00 (0.00-0.00) |
| Major depressive disorder | Index disorder | Major depressive disorder | 0.21 (0.15-0.29) |
| Major depressive disorder | Mental disorders | Alcohol use disorder | 0.01 (0.01-0.01) |
| Major depressive disorder | Mental disorders | Opioid use disorder | 0.00 (0.00-0.00) |
| Major depressive disorder | Mental disorders | Cannabis use disorder | 0.00 (0.00-0.00) |
| Major depressive disorder | Mental disorders | Cocaine use disorder | 0.00 (0.00-0.00) |
| Major depressive disorder | Mental disorders | Amphetamine use disorder | 0.00 (0.00-0.00) |
| Major depressive disorder | Mental disorders | Other drug use disorders | 0.00 (0.00-0.00) |
| Major depressive disorder | Mental disorders | Schizophrenia | 0.01 (0.01-0.02) |
| Major depressive disorder | Mental disorders | Bipolar disorder | 0.01 (0.01-0.01) |
| Major depressive disorder | Mental disorders | Dysthymia | 0.00 (0.00-0.00) |
| Major depressive disorder | Mental disorders | Anxiety disorders | 0.02 (0.01-0.03) |
| Major depressive disorder | Mental disorders | Anorexia | 0.00 (0.00-0.00) |
| Major depressive disorder | Mental disorders | Bulimia | 0.00 (0.00-0.00) |
| Major depressive disorder | Mental disorders | Personality disorders | 0.02 (0.02-0.03) |
| Major depressive disorder | Mental disorders | Intellectual disability | 0.00 (0.00-0.00) |
| Major depressive disorder | Mental disorders | Autism spectrum disorders | 0.00 (0.00-0.00) |
| Major depressive disorder | Mental disorders | ADHD | 0.00 (0.00-0.00) |
| Major depressive disorder | Mental disorders | Conduct disorder | 0.00 (0.00-0.00) |
| Major depressive disorder | Circulatory diseases | Hypertension | 0.01 (0.01-0.01) |
| Major depressive disorder | Circulatory diseases | Dyslipidemia | 0.00 (0.00-0.00) |
| Major depressive disorder | Circulatory diseases | Ischemic heart disease | 0.01 (0.00-0.01) |
| Major depressive disorder | Circulatory diseases | Atrial fibrillation | 0.00 (0.00-0.01) |
| Major depressive disorder | Circulatory diseases | Heart failure | 0.00 (0.00-0.00) |
| Major depressive disorder | Circulatory diseases | Peripheral artery occlusive disease | 0.00 (0.00-0.00) |
| Major depressive disorder | Circulatory diseases | Stroke | 0.00 (0.00-0.01) |
| Major depressive disorder | Endocrine diseases | Diabetes | 0.00 (0.00-0.00) |
| Major depressive disorder | Endocrine diseases | Thyroid disorder | 0.00 (0.00-0.00) |
| Major depressive disorder | Endocrine diseases | Gout | 0.00 (0.00-0.00) |
| Major depressive disorder | Pulmonary diseases | Asthma | 0.01 (0.00-0.01) |
| Major depressive disorder | Pulmonary diseases | Chronic obstructive pulmonary disease | 0.01 (0.01-0.01) |
| Major depressive disorder | Pulmonary diseases | Allergy | 0.03 (0.03-0.05) |
| Major depressive disorder | Gastrointestinal diseases | Ulcer/chronic gastritis | 0.00 (0.00-0.00) |
| Major depressive disorder | Gastrointestinal diseases | Chronic liver disease | 0.00 (0.00-0.00) |
| Major depressive disorder | Gastrointestinal diseases | Inflammatory bowel disease | 0.00 (0.00-0.00) |
| Major depressive disorder | Gastrointestinal diseases | Diverticular disease of intestine | 0.00 (0.00-0.00) |
| Major depressive disorder | Urogenital diseases | Chronic kidney disease | 0.00 (0.00-0.00) |
| Major depressive disorder | Urogenital diseases | Prostate disorders | 0.00 (0.00-0.00) |
| Major depressive disorder | Musculoskeletal diseases | Connective | 0.00 (0.00-0.00) |
| Major depressive disorder | Musculoskeletal diseases | Osteoporosis | 0.00 (0.00-0.00) |
| Major depressive disorder | Musculoskeletal diseases | Painful conditions | 0.04 (0.03-0.05) |
| Major depressive disorder | Hematological diseases | HIV/AIDS | 0.00 (0.00-0.00) |
| Major depressive disorder | Hematological diseases | Anemia | 0.00 (0.00-0.00) |
| Major depressive disorder | Cancers | Cancer | 0.00 (0.00-0.00) |
| Major depressive disorder | Neurological diseases | Vision problems | 0.00 (0.00-0.00) |
| Major depressive disorder | Neurological diseases | Hearing problems | 0.00 (0.00-0.00) |
| Major depressive disorder | Neurological diseases | Migraine | 0.00 (0.00-0.00) |
| Major depressive disorder | Neurological diseases | Epilepsy | 0.00 (0.00-0.00) |
| Major depressive disorder | Neurological diseases | Parkinson’s disease | 0.00 (0.00-0.00) |
| Major depressive disorder | Neurological diseases | Multiple sclerosis | 0.00 (0.00-0.00) |
| Major depressive disorder | Neurological diseases | Neuropathies | 0.00 (0.00-0.00) |
| Anxiety disorders | Index disorder | Anxiety disorders | 0.13 (0.09-0.17) |
| Anxiety disorders | Mental disorders | Alcohol use disorder | 0.01 (0.00-0.01) |
| Anxiety disorders | Mental disorders | Opioid use disorder | 0.00 (0.00-0.00) |
| Anxiety disorders | Mental disorders | Cannabis use disorder | 0.00 (0.00-0.00) |
| Anxiety disorders | Mental disorders | Cocaine use disorder | 0.00 (0.00-0.00) |
| Anxiety disorders | Mental disorders | Amphetamine use disorder | 0.00 (0.00-0.00) |
| Anxiety disorders | Mental disorders | Other drug use disorders | 0.00 (0.00-0.00) |
| Anxiety disorders | Mental disorders | Schizophrenia | 0.02 (0.02-0.03) |
| Anxiety disorders | Mental disorders | Bipolar disorder | 0.01 (0.00-0.01) |
| Anxiety disorders | Mental disorders | Dysthymia | 0.00 (0.00-0.00) |
| Anxiety disorders | Mental disorders | Major depressive disorder | 0.01 (0.01-0.02) |
| Anxiety disorders | Mental disorders | Anorexia | 0.00 (0.00-0.00) |
| Anxiety disorders | Mental disorders | Bulimia | 0.00 (0.00-0.00) |
| Anxiety disorders | Mental disorders | Personality disorders | 0.03 (0.02-0.03) |
| Anxiety disorders | Mental disorders | Intellectual disability | 0.00 (0.00-0.00) |
| Anxiety disorders | Mental disorders | Autism spectrum disorders | 0.00 (0.00-0.00) |
| Anxiety disorders | Mental disorders | ADHD | 0.00 (0.00-0.00) |
| Anxiety disorders | Mental disorders | Conduct disorder | 0.00 (0.00-0.00) |
| Anxiety disorders | Circulatory diseases | Hypertension | 0.01 (0.01-0.01) |
| Anxiety disorders | Circulatory diseases | Dyslipidemia | 0.00 (0.00-0.00) |
| Anxiety disorders | Circulatory diseases | Ischemic heart disease | 0.00 (0.00-0.01) |
| Anxiety disorders | Circulatory diseases | Atrial fibrillation | 0.00 (0.00-0.00) |
| Anxiety disorders | Circulatory diseases | Heart failure | 0.00 (0.00-0.00) |
| Anxiety disorders | Circulatory diseases | Peripheral artery occlusive disease | 0.00 (0.00-0.00) |
| Anxiety disorders | Circulatory diseases | Stroke | 0.00 (0.00-0.00) |
| Anxiety disorders | Endocrine diseases | Diabetes | 0.00 (0.00-0.00) |
| Anxiety disorders | Endocrine diseases | Thyroid disorder | 0.00 (0.00-0.00) |
| Anxiety disorders | Endocrine diseases | Gout | 0.00 (0.00-0.00) |
| Anxiety disorders | Pulmonary diseases | Asthma | 0.01 (0.01-0.01) |
| Anxiety disorders | Pulmonary diseases | Chronic obstructive pulmonary disease | 0.01 (0.01-0.01) |
| Anxiety disorders | Pulmonary diseases | Allergy | 0.04 (0.03-0.05) |
| Anxiety disorders | Gastrointestinal diseases | Ulcer/chronic gastritis | 0.00 (0.00-0.00) |
| Anxiety disorders | Gastrointestinal diseases | Chronic liver disease | 0.00 (0.00-0.00) |
| Anxiety disorders | Gastrointestinal diseases | Inflammatory bowel disease | 0.00 (0.00-0.00) |
| Anxiety disorders | Gastrointestinal diseases | Diverticular disease of intestine | 0.00 (0.00-0.00) |
| Anxiety disorders | Urogenital diseases | Chronic kidney disease | 0.00 (0.00-0.00) |
| Anxiety disorders | Urogenital diseases | Prostate disorders | 0.00 (0.00-0.00) |
| Anxiety disorders | Musculoskeletal diseases | Connective | 0.00 (0.00-0.00) |
| Anxiety disorders | Musculoskeletal diseases | Osteoporosis | 0.00 (0.00-0.00) |
| Anxiety disorders | Musculoskeletal diseases | Painful conditions | 0.04 (0.03-0.05) |
| Anxiety disorders | Hematological diseases | HIV/AIDS | 0.00 (0.00-0.00) |
| Anxiety disorders | Hematological diseases | Anemia | 0.00 (0.00-0.00) |
| Anxiety disorders | Cancers | Cancer | 0.00 (0.00-0.00) |
| Anxiety disorders | Neurological diseases | Vision problems | 0.00 (0.00-0.00) |
| Anxiety disorders | Neurological diseases | Hearing problems | 0.00 (0.00-0.00) |
| Anxiety disorders | Neurological diseases | Migraine | 0.00 (0.00-0.00) |
| Anxiety disorders | Neurological diseases | Epilepsy | 0.00 (0.00-0.01) |
| Anxiety disorders | Neurological diseases | Parkinson’s disease | 0.00 (0.00-0.00) |
| Anxiety disorders | Neurological diseases | Multiple sclerosis | 0.00 (0.00-0.00) |
| Anxiety disorders | Neurological diseases | Neuropathies | 0.00 (0.00-0.00) |
| Anorexia | Index disorder | Anorexia | 0.20 (0.15-0.26) |
| Anorexia | Mental disorders | Alcohol use disorder | 0.00 (0.00-0.00) |
| Anorexia | Mental disorders | Opioid use disorder | 0.00 (0.00-0.00) |
| Anorexia | Mental disorders | Cannabis use disorder | 0.00 (0.00-0.00) |
| Anorexia | Mental disorders | Cocaine use disorder | 0.00 (0.00-0.00) |
| Anorexia | Mental disorders | Amphetamine use disorder | 0.00 (0.00-0.00) |
| Anorexia | Mental disorders | Other drug use disorders | 0.00 (0.00-0.00) |
| Anorexia | Mental disorders | Schizophrenia | 0.02 (0.02-0.03) |
| Anorexia | Mental disorders | Bipolar disorder | 0.00 (0.00-0.00) |
| Anorexia | Mental disorders | Dysthymia | 0.00 (0.00-0.00) |
| Anorexia | Mental disorders | Major depressive disorder | 0.01 (0.01-0.01) |
| Anorexia | Mental disorders | Anxiety disorders | 0.02 (0.01-0.02) |
| Anorexia | Mental disorders | Bulimia | 0.01 (0.01-0.01) |
| Anorexia | Mental disorders | Personality disorders | 0.03 (0.02-0.03) |
| Anorexia | Mental disorders | Intellectual disability | 0.00 (0.00-0.00) |
| Anorexia | Mental disorders | Autism spectrum disorders | 0.00 (0.00-0.00) |
| Anorexia | Mental disorders | ADHD | 0.00 (0.00-0.00) |
| Anorexia | Mental disorders | Conduct disorder | 0.00 (0.00-0.00) |
| Anorexia | Circulatory diseases | Hypertension | 0.00 (0.00-0.00) |
| Anorexia | Circulatory diseases | Dyslipidemia | 0.00 (0.00-0.00) |
| Anorexia | Circulatory diseases | Ischemic heart disease | 0.00 (0.00-0.00) |
| Anorexia | Circulatory diseases | Atrial fibrillation | 0.00 (0.00-0.00) |
| Anorexia | Circulatory diseases | Heart failure | 0.00 (0.00-0.00) |
| Anorexia | Circulatory diseases | Peripheral artery occlusive disease | 0.00 (0.00-0.00) |
| Anorexia | Circulatory diseases | Stroke | 0.00 (0.00-0.00) |
| Anorexia | Endocrine diseases | Diabetes | 0.00 (0.00-0.00) |
| Anorexia | Endocrine diseases | Thyroid disorder | 0.00 (0.00-0.00) |
| Anorexia | Endocrine diseases | Gout | 0.00 (0.00-0.00) |
| Anorexia | Pulmonary diseases | Asthma | 0.01 (0.01-0.01) |
| Anorexia | Pulmonary diseases | Chronic obstructive pulmonary disease | 0.00 (0.00-0.00) |
| Anorexia | Pulmonary diseases | Allergy | 0.03 (0.02-0.04) |
| Anorexia | Gastrointestinal diseases | Ulcer/chronic gastritis | 0.00 (0.00-0.00) |
| Anorexia | Gastrointestinal diseases | Chronic liver disease | 0.00 (0.00-0.00) |
| Anorexia | Gastrointestinal diseases | Inflammatory bowel disease | 0.00 (0.00-0.00) |
| Anorexia | Gastrointestinal diseases | Diverticular disease of intestine | 0.00 (0.00-0.00) |
| Anorexia | Urogenital diseases | Other | 0.00 (0.00-0.00) |
| Anorexia | Musculoskeletal diseases | Connective | 0.00 (0.00-0.00) |
| Anorexia | Musculoskeletal diseases | Osteoporosis | 0.00 (0.00-0.01) |
| Anorexia | Musculoskeletal diseases | Painful conditions | 0.02 (0.01-0.02) |
| Anorexia | Hematological diseases | HIV/AIDS | 0.00 (0.00-0.00) |
| Anorexia | Hematological diseases | Anemia | 0.00 (0.00-0.00) |
| Anorexia | Cancers | Cancer | 0.00 (0.00-0.00) |
| Anorexia | Neurological diseases | Vision problems | 0.00 (0.00-0.00) |
| Anorexia | Neurological diseases | Hearing problems | 0.00 (0.00-0.00) |
| Anorexia | Neurological diseases | Migraine | 0.00 (0.00-0.00) |
| Anorexia | Neurological diseases | Epilepsy | 0.00 (0.00-0.00) |
| Anorexia | Neurological diseases | Parkinson’s disease | 0.00 (0.00-0.00) |
| Anorexia | Neurological diseases | Multiple sclerosis | 0.00 (0.00-0.00) |
| Anorexia | Neurological diseases | Neuropathies | 0.00 (0.00-0.00) |
| Bulimia | Index disorder | Bulimia | 0.20 (0.15-0.25) |
| Bulimia | Mental disorders | Alcohol use disorder | 0.00 (0.00-0.01) |
| Bulimia | Mental disorders | Opioid use disorder | 0.00 (0.00-0.00) |
| Bulimia | Mental disorders | Cannabis use disorder | 0.00 (0.00-0.00) |
| Bulimia | Mental disorders | Amphetamine use disorder | 0.00 (0.00-0.00) |
| Bulimia | Mental disorders | Other drug use disorders | 0.00 (0.00-0.00) |
| Bulimia | Mental disorders | Schizophrenia | 0.02 (0.01-0.02) |
| Bulimia | Mental disorders | Bipolar disorder | 0.00 (0.00-0.00) |
| Bulimia | Mental disorders | Dysthymia | 0.00 (0.00-0.00) |
| Bulimia | Mental disorders | Major depressive disorder | 0.01 (0.01-0.02) |
| Bulimia | Mental disorders | Anxiety disorders | 0.01 (0.01-0.02) |
| Bulimia | Mental disorders | Anorexia | 0.02 (0.01-0.02) |
| Bulimia | Mental disorders | Personality disorders | 0.04 (0.03-0.05) |
| Bulimia | Mental disorders | Intellectual disability | 0.00 (0.00-0.00) |
| Bulimia | Mental disorders | Autism spectrum disorders | 0.00 (0.00-0.00) |
| Bulimia | Mental disorders | ADHD | 0.00 (0.00-0.00) |
| Bulimia | Mental disorders | Other | 0.00 (0.00-0.00) |
| Bulimia | Circulatory diseases | Hypertension | 0.00 (0.00-0.01) |
| Bulimia | Circulatory diseases | Dyslipidemia | 0.00 (0.00-0.00) |
| Bulimia | Circulatory diseases | Ischemic heart disease | 0.00 (0.00-0.00) |
| Bulimia | Circulatory diseases | Atrial fibrillation | 0.00 (0.00-0.00) |
| Bulimia | Circulatory diseases | Heart failure | 0.00 (0.00-0.00) |
| Bulimia | Circulatory diseases | Peripheral artery occlusive disease | 0.00 (0.00-0.00) |
| Bulimia | Circulatory diseases | Stroke | 0.00 (0.00-0.00) |
| Bulimia | Endocrine diseases | Diabetes | 0.00 (0.00-0.00) |
| Bulimia | Endocrine diseases | Thyroid disorder | 0.00 (0.00-0.00) |
| Bulimia | Endocrine diseases | Gout | 0.00 (0.00-0.00) |
| Bulimia | Pulmonary diseases | Asthma | 0.01 (0.01-0.01) |
| Bulimia | Pulmonary diseases | Chronic obstructive pulmonary disease | 0.00 (0.00-0.00) |
| Bulimia | Pulmonary diseases | Allergy | 0.03 (0.03-0.05) |
| Bulimia | Gastrointestinal diseases | Ulcer/chronic gastritis | 0.00 (0.00-0.00) |
| Bulimia | Gastrointestinal diseases | Chronic liver disease | 0.00 (0.00-0.00) |
| Bulimia | Gastrointestinal diseases | Inflammatory bowel disease | 0.00 (0.00-0.00) |
| Bulimia | Gastrointestinal diseases | Diverticular disease of intestine | 0.00 (0.00-0.00) |
| Bulimia | Urogenital diseases | Other | 0.00 (0.00-0.00) |
| Bulimia | Musculoskeletal diseases | Connective | 0.00 (0.00-0.00) |
| Bulimia | Musculoskeletal diseases | Osteoporosis | 0.00 (0.00-0.00) |
| Bulimia | Musculoskeletal diseases | Painful conditions | 0.02 (0.01-0.03) |
| Bulimia | Hematological diseases | Other | 0.00 (0.00-0.00) |
| Bulimia | Cancers | Cancer | 0.00 (0.00-0.00) |
| Bulimia | Neurological diseases | Vision problems | 0.00 (0.00-0.00) |
| Bulimia | Neurological diseases | Hearing problems | 0.00 (0.00-0.00) |
| Bulimia | Neurological diseases | Migraine | 0.00 (0.00-0.00) |
| Bulimia | Neurological diseases | Epilepsy | 0.00 (0.00-0.00) |
| Bulimia | Neurological diseases | Multiple sclerosis | 0.00 (0.00-0.00) |
| Bulimia | Neurological diseases | Neuropathies | 0.00 (0.00-0.00) |
| Bulimia | Neurological diseases | Other | 0.00 (0.00-0.00) |
| Personality disorders | Index disorder | Personality disorders | 0.13 (0.09-0.17) |
| Personality disorders | Mental disorders | Alcohol use disorder | 0.01 (0.01-0.01) |
| Personality disorders | Mental disorders | Opioid use disorder | 0.01 (0.00-0.01) |
| Personality disorders | Mental disorders | Cannabis use disorder | 0.00 (0.00-0.00) |
| Personality disorders | Mental disorders | Cocaine use disorder | 0.00 (0.00-0.00) |
| Personality disorders | Mental disorders | Amphetamine use disorder | 0.00 (0.00-0.00) |
| Personality disorders | Mental disorders | Other drug use disorders | 0.00 (0.00-0.00) |
| Personality disorders | Mental disorders | Schizophrenia | 0.05 (0.04-0.05) |
| Personality disorders | Mental disorders | Bipolar disorder | 0.01 (0.01-0.01) |
| Personality disorders | Mental disorders | Dysthymia | 0.00 (0.00-0.00) |
| Personality disorders | Mental disorders | Major depressive disorder | 0.01 (0.01-0.01) |
| Personality disorders | Mental disorders | Anxiety disorders | 0.02 (0.01-0.03) |
| Personality disorders | Mental disorders | Anorexia | 0.00 (0.00-0.00) |
| Personality disorders | Mental disorders | Bulimia | 0.00 (0.00-0.00) |
| Personality disorders | Mental disorders | Intellectual disability | 0.00 (0.00-0.00) |
| Personality disorders | Mental disorders | Autism spectrum disorders | 0.00 (0.00-0.00) |
| Personality disorders | Mental disorders | ADHD | 0.00 (0.00-0.00) |
| Personality disorders | Mental disorders | Conduct disorder | 0.00 (0.00-0.00) |
| Personality disorders | Circulatory diseases | Hypertension | 0.01 (0.01-0.01) |
| Personality disorders | Circulatory diseases | Dyslipidemia | 0.00 (0.00-0.00) |
| Personality disorders | Circulatory diseases | Ischemic heart disease | 0.00 (0.00-0.00) |
| Personality disorders | Circulatory diseases | Atrial fibrillation | 0.00 (0.00-0.00) |
| Personality disorders | Circulatory diseases | Heart failure | 0.00 (0.00-0.00) |
| Personality disorders | Circulatory diseases | Peripheral artery occlusive disease | 0.00 (0.00-0.00) |
| Personality disorders | Circulatory diseases | Stroke | 0.00 (0.00-0.00) |
| Personality disorders | Endocrine diseases | Diabetes | 0.00 (0.00-0.00) |
| Personality disorders | Endocrine diseases | Thyroid disorder | 0.00 (0.00-0.00) |
| Personality disorders | Endocrine diseases | Gout | 0.00 (0.00-0.00) |
| Personality disorders | Pulmonary diseases | Asthma | 0.01 (0.01-0.01) |
| Personality disorders | Pulmonary diseases | Chronic obstructive pulmonary disease | 0.01 (0.01-0.01) |
| Personality disorders | Pulmonary diseases | Allergy | 0.04 (0.03-0.05) |
| Personality disorders | Gastrointestinal diseases | Ulcer/chronic gastritis | 0.00 (0.00-0.00) |
| Personality disorders | Gastrointestinal diseases | Chronic liver disease | 0.00 (0.00-0.00) |
| Personality disorders | Gastrointestinal diseases | Inflammatory bowel disease | 0.00 (0.00-0.00) |
| Personality disorders | Gastrointestinal diseases | Diverticular disease of intestine | 0.00 (0.00-0.00) |
| Personality disorders | Urogenital diseases | Chronic kidney disease | 0.00 (0.00-0.00) |
| Personality disorders | Urogenital diseases | Prostate disorders | 0.00 (0.00-0.00) |
| Personality disorders | Musculoskeletal diseases | Connective | 0.00 (0.00-0.00) |
| Personality disorders | Musculoskeletal diseases | Osteoporosis | 0.00 (0.00-0.00) |
| Personality disorders | Musculoskeletal diseases | Painful conditions | 0.04 (0.03-0.06) |
| Personality disorders | Hematological diseases | HIV/AIDS | 0.00 (0.00-0.00) |
| Personality disorders | Hematological diseases | Anemia | 0.00 (0.00-0.00) |
| Personality disorders | Cancers | Cancer | 0.00 (0.00-0.00) |
| Personality disorders | Neurological diseases | Vision problems | 0.00 (0.00-0.00) |
| Personality disorders | Neurological diseases | Hearing problems | 0.00 (0.00-0.00) |
| Personality disorders | Neurological diseases | Migraine | 0.00 (0.00-0.00) |
| Personality disorders | Neurological diseases | Epilepsy | 0.01 (0.00-0.01) |
| Personality disorders | Neurological diseases | Parkinson’s disease | 0.00 (0.00-0.00) |
| Personality disorders | Neurological diseases | Multiple sclerosis | 0.00 (0.00-0.00) |
| Personality disorders | Neurological diseases | Neuropathies | 0.00 (0.00-0.00) |
| Intellectual disability | Index disorder | Intellectual disability | 0.06 (0.02-0.12) |
| Intellectual disability | Mental disorders | Alcohol use disorder | 0.00 (0.00-0.00) |
| Intellectual disability | Mental disorders | Opioid use disorder | 0.00 (0.00-0.00) |
| Intellectual disability | Mental disorders | Cannabis use disorder | 0.00 (0.00-0.00) |
| Intellectual disability | Mental disorders | Cocaine use disorder | 0.00 (0.00-0.00) |
| Intellectual disability | Mental disorders | Amphetamine use disorder | 0.00 (0.00-0.00) |
| Intellectual disability | Mental disorders | Other drug use disorders | 0.00 (0.00-0.00) |
| Intellectual disability | Mental disorders | Schizophrenia | 0.04 (0.04-0.05) |
| Intellectual disability | Mental disorders | Bipolar disorder | 0.01 (0.00-0.01) |
| Intellectual disability | Mental disorders | Dysthymia | 0.00 (0.00-0.00) |
| Intellectual disability | Mental disorders | Major depressive disorder | 0.00 (0.00-0.00) |
| Intellectual disability | Mental disorders | Anxiety disorders | 0.01 (0.01-0.01) |
| Intellectual disability | Mental disorders | Anorexia | 0.00 (0.00-0.00) |
| Intellectual disability | Mental disorders | Bulimia | 0.00 (0.00-0.00) |
| Intellectual disability | Mental disorders | Personality disorders | 0.01 (0.01-0.01) |
| Intellectual disability | Mental disorders | Autism spectrum disorders | 0.03 (0.02-0.04) |
| Intellectual disability | Mental disorders | ADHD | 0.00 (0.00-0.01) |
| Intellectual disability | Mental disorders | Conduct disorder | 0.00 (0.00-0.00) |
| Intellectual disability | Circulatory diseases | Hypertension | 0.01 (0.00-0.01) |
| Intellectual disability | Circulatory diseases | Dyslipidemia | 0.00 (0.00-0.00) |
| Intellectual disability | Circulatory diseases | Ischemic heart disease | 0.00 (0.00-0.00) |
| Intellectual disability | Circulatory diseases | Atrial fibrillation | 0.00 (0.00-0.00) |
| Intellectual disability | Circulatory diseases | Heart failure | 0.00 (0.00-0.00) |
| Intellectual disability | Circulatory diseases | Peripheral artery occlusive disease | 0.00 (0.00-0.00) |
| Intellectual disability | Circulatory diseases | Stroke | 0.00 (0.00-0.00) |
| Intellectual disability | Endocrine diseases | Diabetes | 0.00 (0.00-0.00) |
| Intellectual disability | Endocrine diseases | Thyroid disorder | 0.00 (0.00-0.00) |
| Intellectual disability | Endocrine diseases | Gout | 0.00 (0.00-0.00) |
| Intellectual disability | Pulmonary diseases | Asthma | 0.01 (0.01-0.01) |
| Intellectual disability | Pulmonary diseases | Chronic obstructive pulmonary disease | 0.00 (0.00-0.01) |
| Intellectual disability | Pulmonary diseases | Allergy | 0.03 (0.02-0.04) |
| Intellectual disability | Gastrointestinal diseases | Ulcer/chronic gastritis | 0.00 (0.00-0.00) |
| Intellectual disability | Gastrointestinal diseases | Chronic liver disease | 0.00 (0.00-0.00) |
| Intellectual disability | Gastrointestinal diseases | Inflammatory bowel disease | 0.00 (0.00-0.00) |
| Intellectual disability | Gastrointestinal diseases | Diverticular disease of intestine | 0.00 (0.00-0.00) |
| Intellectual disability | Urogenital diseases | Chronic kidney disease | 0.00 (0.00-0.00) |
| Intellectual disability | Urogenital diseases | Prostate disorders | 0.00 (0.00-0.00) |
| Intellectual disability | Musculoskeletal diseases | Connective | 0.00 (0.00-0.00) |
| Intellectual disability | Musculoskeletal diseases | Osteoporosis | 0.00 (0.00-0.00) |
| Intellectual disability | Musculoskeletal diseases | Painful conditions | 0.02 (0.02-0.03) |
| Intellectual disability | Hematological diseases | HIV/AIDS | 0.00 (0.00-0.00) |
| Intellectual disability | Hematological diseases | Anemia | 0.00 (0.00-0.00) |
| Intellectual disability | Cancers | Cancer | 0.00 (0.00-0.00) |
| Intellectual disability | Neurological diseases | Vision problems | 0.00 (0.00-0.00) |
| Intellectual disability | Neurological diseases | Hearing problems | 0.00 (0.00-0.00) |
| Intellectual disability | Neurological diseases | Migraine | 0.00 (0.00-0.00) |
| Intellectual disability | Neurological diseases | Epilepsy | 0.03 (0.02-0.04) |
| Intellectual disability | Neurological diseases | Parkinson’s disease | 0.00 (0.00-0.00) |
| Intellectual disability | Neurological diseases | Multiple sclerosis | 0.00 (0.00-0.00) |
| Intellectual disability | Neurological diseases | Neuropathies | 0.00 (0.00-0.00) |
| Autism spectrum disorders | Index disorder | Autism spectrum disorders | 0.16 (0.12-0.20) |
| Autism spectrum disorders | Mental disorders | Alcohol use disorder | 0.00 (0.00-0.00) |
| Autism spectrum disorders | Mental disorders | Opioid use disorder | 0.00 (0.00-0.00) |
| Autism spectrum disorders | Mental disorders | Cannabis use disorder | 0.00 (0.00-0.00) |
| Autism spectrum disorders | Mental disorders | Cocaine use disorder | 0.00 (0.00-0.00) |
| Autism spectrum disorders | Mental disorders | Amphetamine use disorder | 0.00 (0.00-0.00) |
| Autism spectrum disorders | Mental disorders | Other drug use disorders | 0.00 (0.00-0.00) |
| Autism spectrum disorders | Mental disorders | Schizophrenia | 0.01 (0.01-0.02) |
| Autism spectrum disorders | Mental disorders | Bipolar disorder | 0.00 (0.00-0.00) |
| Autism spectrum disorders | Mental disorders | Dysthymia | 0.00 (0.00-0.00) |
| Autism spectrum disorders | Mental disorders | Major depressive disorder | 0.00 (0.00-0.00) |
| Autism spectrum disorders | Mental disorders | Anxiety disorders | 0.01 (0.01-0.01) |
| Autism spectrum disorders | Mental disorders | Anorexia | 0.00 (0.00-0.00) |
| Autism spectrum disorders | Mental disorders | Bulimia | 0.00 (0.00-0.00) |
| Autism spectrum disorders | Mental disorders | Personality disorders | 0.00 (0.00-0.01) |
| Autism spectrum disorders | Mental disorders | Intellectual disability | 0.01 (0.00-0.02) |
| Autism spectrum disorders | Mental disorders | ADHD | 0.01 (0.01-0.01) |
| Autism spectrum disorders | Mental disorders | Conduct disorder | 0.00 (0.00-0.00) |
| Autism spectrum disorders | Circulatory diseases | Hypertension | 0.00 (0.00-0.00) |
| Autism spectrum disorders | Circulatory diseases | Dyslipidemia | 0.00 (0.00-0.00) |
| Autism spectrum disorders | Circulatory diseases | Ischemic heart disease | 0.00 (0.00-0.00) |
| Autism spectrum disorders | Circulatory diseases | Atrial fibrillation | 0.00 (0.00-0.00) |
| Autism spectrum disorders | Circulatory diseases | Heart failure | 0.00 (0.00-0.00) |
| Autism spectrum disorders | Circulatory diseases | Peripheral artery occlusive disease | 0.00 (0.00-0.00) |
| Autism spectrum disorders | Circulatory diseases | Stroke | 0.00 (0.00-0.00) |
| Autism spectrum disorders | Endocrine diseases | Diabetes | 0.00 (0.00-0.00) |
| Autism spectrum disorders | Endocrine diseases | Thyroid disorder | 0.00 (0.00-0.00) |
| Autism spectrum disorders | Endocrine diseases | Gout | 0.00 (0.00-0.00) |
| Autism spectrum disorders | Pulmonary diseases | Asthma | 0.02 (0.01-0.02) |
| Autism spectrum disorders | Pulmonary diseases | Chronic obstructive pulmonary disease | 0.00 (0.00-0.00) |
| Autism spectrum disorders | Pulmonary diseases | Allergy | 0.03 (0.02-0.04) |
| Autism spectrum disorders | Gastrointestinal diseases | Ulcer/chronic gastritis | 0.00 (0.00-0.00) |
| Autism spectrum disorders | Gastrointestinal diseases | Chronic liver disease | 0.00 (0.00-0.00) |
| Autism spectrum disorders | Gastrointestinal diseases | Inflammatory bowel disease | 0.00 (0.00-0.00) |
| Autism spectrum disorders | Gastrointestinal diseases | Diverticular disease of intestine | 0.00 (0.00-0.00) |
| Autism spectrum disorders | Urogenital diseases | Chronic kidney disease | 0.00 (0.00-0.00) |
| Autism spectrum disorders | Urogenital diseases | Prostate disorders | 0.00 (0.00-0.00) |
| Autism spectrum disorders | Musculoskeletal diseases | Connective | 0.00 (0.00-0.00) |
| Autism spectrum disorders | Musculoskeletal diseases | Osteoporosis | 0.00 (0.00-0.00) |
| Autism spectrum disorders | Musculoskeletal diseases | Painful conditions | 0.01 (0.00-0.01) |
| Autism spectrum disorders | Hematological diseases | HIV/AIDS | 0.00 (0.00-0.00) |
| Autism spectrum disorders | Hematological diseases | Anemia | 0.00 (0.00-0.00) |
| Autism spectrum disorders | Cancers | Cancer | 0.00 (0.00-0.00) |
| Autism spectrum disorders | Neurological diseases | Vision problems | 0.00 (0.00-0.00) |
| Autism spectrum disorders | Neurological diseases | Hearing problems | 0.00 (0.00-0.00) |
| Autism spectrum disorders | Neurological diseases | Migraine | 0.00 (0.00-0.00) |
| Autism spectrum disorders | Neurological diseases | Epilepsy | 0.01 (0.01-0.01) |
| Autism spectrum disorders | Neurological diseases | Parkinson’s disease | 0.00 (0.00-0.00) |
| Autism spectrum disorders | Neurological diseases | Multiple sclerosis | 0.00 (0.00-0.00) |
| Autism spectrum disorders | Neurological diseases | Neuropathies | 0.00 (0.00-0.00) |
| ADHD | Index disorder | ADHD | 0.04 (0.03-0.06) |
| ADHD | Mental disorders | Alcohol use disorder | 0.00 (0.00-0.00) |
| ADHD | Mental disorders | Opioid use disorder | 0.00 (0.00-0.00) |
| ADHD | Mental disorders | Cannabis use disorder | 0.00 (0.00-0.00) |
| ADHD | Mental disorders | Cocaine use disorder | 0.00 (0.00-0.00) |
| ADHD | Mental disorders | Amphetamine use disorder | 0.00 (0.00-0.00) |
| ADHD | Mental disorders | Other drug use disorders | 0.00 (0.00-0.00) |
| ADHD | Mental disorders | Schizophrenia | 0.01 (0.01-0.01) |
| ADHD | Mental disorders | Bipolar disorder | 0.00 (0.00-0.00) |
| ADHD | Mental disorders | Dysthymia | 0.00 (0.00-0.00) |
| ADHD | Mental disorders | Major depressive disorder | 0.00 (0.00-0.01) |
| ADHD | Mental disorders | Anxiety disorders | 0.01 (0.01-0.01) |
| ADHD | Mental disorders | Anorexia | 0.00 (0.00-0.00) |
| ADHD | Mental disorders | Bulimia | 0.00 (0.00-0.00) |
| ADHD | Mental disorders | Personality disorders | 0.01 (0.01-0.02) |
| ADHD | Mental disorders | Intellectual disability | 0.01 (0.00-0.01) |
| ADHD | Mental disorders | Autism spectrum disorders | 0.02 (0.02-0.03) |
| ADHD | Mental disorders | Conduct disorder | 0.00 (0.00-0.00) |
| ADHD | Circulatory diseases | Hypertension | 0.00 (0.00-0.00) |
| ADHD | Circulatory diseases | Dyslipidemia | 0.00 (0.00-0.00) |
| ADHD | Circulatory diseases | Ischemic heart disease | 0.00 (0.00-0.00) |
| ADHD | Circulatory diseases | Atrial fibrillation | 0.00 (0.00-0.00) |
| ADHD | Circulatory diseases | Heart failure | 0.00 (0.00-0.00) |
| ADHD | Circulatory diseases | Peripheral artery occlusive disease | 0.00 (0.00-0.00) |
| ADHD | Circulatory diseases | Stroke | 0.00 (0.00-0.00) |
| ADHD | Endocrine diseases | Diabetes | 0.00 (0.00-0.00) |
| ADHD | Endocrine diseases | Thyroid disorder | 0.00 (0.00-0.00) |
| ADHD | Endocrine diseases | Gout | 0.00 (0.00-0.00) |
| ADHD | Pulmonary diseases | Asthma | 0.02 (0.01-0.03) |
| ADHD | Pulmonary diseases | Chronic obstructive pulmonary disease | 0.00 (0.00-0.00) |
| ADHD | Pulmonary diseases | Allergy | 0.03 (0.02-0.04) |
| ADHD | Gastrointestinal diseases | Ulcer/chronic gastritis | 0.00 (0.00-0.00) |
| ADHD | Gastrointestinal diseases | Chronic liver disease | 0.00 (0.00-0.00) |
| ADHD | Gastrointestinal diseases | Inflammatory bowel disease | 0.00 (0.00-0.00) |
| ADHD | Gastrointestinal diseases | Diverticular disease of intestine | 0.00 (0.00-0.00) |
| ADHD | Urogenital diseases | Chronic kidney disease | 0.00 (0.00-0.00) |
| ADHD | Urogenital diseases | Prostate disorders | 0.00 (0.00-0.00) |
| ADHD | Musculoskeletal diseases | Connective | 0.00 (0.00-0.00) |
| ADHD | Musculoskeletal diseases | Osteoporosis | 0.00 (0.00-0.00) |
| ADHD | Musculoskeletal diseases | Painful conditions | 0.01 (0.01-0.02) |
| ADHD | Hematological diseases | HIV/AIDS | 0.00 (0.00-0.00) |
| ADHD | Hematological diseases | Anemia | 0.00 (0.00-0.00) |
| ADHD | Cancers | Cancer | 0.00 (0.00-0.00) |
| ADHD | Neurological diseases | Vision problems | 0.00 (0.00-0.00) |
| ADHD | Neurological diseases | Hearing problems | 0.00 (0.00-0.00) |
| ADHD | Neurological diseases | Migraine | 0.00 (0.00-0.00) |
| ADHD | Neurological diseases | Epilepsy | 0.01 (0.00-0.01) |
| ADHD | Neurological diseases | Parkinson’s disease | 0.00 (0.00-0.00) |
| ADHD | Neurological diseases | Multiple sclerosis | 0.00 (0.00-0.00) |
| ADHD | Neurological diseases | Neuropathies | 0.00 (0.00-0.00) |
| Conduct disorder | Index disorder | Conduct disorder | 0.22 (0.17-0.29) |
| Conduct disorder | Mental disorders | Alcohol use disorder | 0.00 (0.00-0.00) |
| Conduct disorder | Mental disorders | Opioid use disorder | 0.00 (0.00-0.00) |
| Conduct disorder | Mental disorders | Cannabis use disorder | 0.00 (0.00-0.00) |
| Conduct disorder | Mental disorders | Cocaine use disorder | 0.00 (0.00-0.00) |
| Conduct disorder | Mental disorders | Amphetamine use disorder | 0.00 (0.00-0.00) |
| Conduct disorder | Mental disorders | Other drug use disorders | 0.00 (0.00-0.00) |
| Conduct disorder | Mental disorders | Schizophrenia | 0.01 (0.01-0.02) |
| Conduct disorder | Mental disorders | Bipolar disorder | 0.00 (0.00-0.00) |
| Conduct disorder | Mental disorders | Major depressive disorder | 0.00 (0.00-0.00) |
| Conduct disorder | Mental disorders | Anxiety disorders | 0.01 (0.00-0.01) |
| Conduct disorder | Mental disorders | Anorexia | 0.00 (0.00-0.00) |
| Conduct disorder | Mental disorders | Personality disorders | 0.01 (0.01-0.01) |
| Conduct disorder | Mental disorders | Intellectual disability | 0.01 (0.00-0.01) |
| Conduct disorder | Mental disorders | Autism spectrum disorders | 0.01 (0.01-0.02) |
| Conduct disorder | Mental disorders | ADHD | 0.01 (0.01-0.01) |
| Conduct disorder | Mental disorders | Other | 0.00 (0.00-0.00) |
| Conduct disorder | Circulatory diseases | Hypertension | 0.00 (0.00-0.00) |
| Conduct disorder | Circulatory diseases | Dyslipidemia | 0.00 (0.00-0.00) |
| Conduct disorder | Circulatory diseases | Ischemic heart disease | 0.00 (0.00-0.00) |
| Conduct disorder | Circulatory diseases | Atrial fibrillation | 0.00 (0.00-0.00) |
| Conduct disorder | Circulatory diseases | Other | 0.00 (0.00-0.00) |
| Conduct disorder | Endocrine diseases | Thyroid disorder | 0.00 (0.00-0.00) |
| Conduct disorder | Endocrine diseases | Other | 0.00 (0.00-0.00) |
| Conduct disorder | Pulmonary diseases | Asthma | 0.02 (0.01-0.02) |
| Conduct disorder | Pulmonary diseases | Chronic obstructive pulmonary disease | 0.00 (0.00-0.00) |
| Conduct disorder | Pulmonary diseases | Allergy | 0.03 (0.02-0.03) |
| Conduct disorder | Gastrointestinal diseases | Ulcer/chronic gastritis | 0.00 (0.00-0.00) |
| Conduct disorder | Gastrointestinal diseases | Chronic liver disease | 0.00 (0.00-0.00) |
| Conduct disorder | Gastrointestinal diseases | Other | 0.00 (0.00-0.00) |
| Conduct disorder | Musculoskeletal diseases | Connective | 0.00 (0.00-0.00) |
| Conduct disorder | Musculoskeletal diseases | Osteoporosis | 0.00 (0.00-0.00) |
| Conduct disorder | Musculoskeletal diseases | Painful conditions | 0.00 (0.00-0.01) |
| Conduct disorder | Hematological diseases | Anemia | 0.00 (0.00-0.00) |
| Conduct disorder | Hematological diseases | Other | 0.00 (0.00-0.00) |
| Conduct disorder | Neurological diseases | Vision problems | 0.00 (0.00-0.00) |
| Conduct disorder | Neurological diseases | Hearing problems | 0.00 (0.00-0.00) |
| Conduct disorder | Neurological diseases | Migraine | 0.00 (0.00-0.00) |
| Conduct disorder | Neurological diseases | Epilepsy | 0.00 (0.00-0.01) |
| Conduct disorder | Neurological diseases | Neuropathies | 0.00 (0.00-0.00) |
| Conduct disorder | Neurological diseases | Other | 0.00 (0.00-0.00) |
| Conduct disorder | Other | Other | 0.00 (0.00-0.00) |

**Supplementary table 5.** Age-specific health loss proportion (HeLP) and 95% confidence interval for persons diagnosed with alcohol use disorder during 2000-2015. All estimates are adjusted for observed comorbidity from mental and substance use disorders and general medical conditions.

|  | Index disorder | Mental disorders | Circulatory diseases | Endocrine diseases | Pulmonary diseases | Gastrointestinal diseases | Urogenital diseases | Musculoskeletal diseases | Hematological diseases | Cancers | Neurological diseases | Other |
| --- | --- | --- | --- | --- | --- | --- | --- | --- | --- | --- | --- | --- |
| 15-19 | 0.16 (0.11-0.22) | 0.19 (0.16-0.21) | 0.00 (0.00-0.00) | | 0.05 (0.04-0.06) | 0.00 (0.00-0.00) | | 0.01 (0.00-0.01) | 0.00 (0.00-0.00) | | 0.00 (0.00-0.01) | 0.00 (0.00-0.00) |
| 20-24 | 0.15 (0.11-0.21) | 0.25 (0.23-0.28) | 0.00 (0.00-0.00) | 0.00 (0.00-0.00) | 0.03 (0.03-0.04) | 0.00 (0.00-0.00) | 0.00 (0.00-0.00) | 0.01 (0.01-0.02) | 0.00 (0.00-0.00) | 0.00 (0.00-0.00) | 0.01 (0.00-0.01) | |
| 25-29 | 0.15 (0.11-0.21) | 0.26 (0.23-0.28) | 0.00 (0.00-0.01) | 0.00 (0.00-0.00) | 0.03 (0.02-0.04) | 0.01 (0.00-0.01) | 0.00 (0.00-0.00) | 0.02 (0.01-0.02) | 0.00 (0.00-0.00) | 0.00 (0.00-0.00) | 0.01 (0.01-0.01) | |
| 30-34 | 0.15 (0.11-0.21) | 0.24 (0.22-0.27) | 0.01 (0.01-0.01) | 0.00 (0.00-0.00) | 0.03 (0.02-0.04) | 0.01 (0.01-0.01) | 0.00 (0.00-0.00) | 0.03 (0.02-0.03) | 0.00 (0.00-0.00) | 0.00 (0.00-0.00) | 0.01 (0.01-0.01) | |
| 35-39 | 0.15 (0.11-0.21) | 0.21 (0.19-0.23) | 0.01 (0.01-0.01) | 0.00 (0.00-0.00) | 0.04 (0.03-0.04) | 0.01 (0.01-0.01) | 0.00 (0.00-0.00) | 0.04 (0.03-0.05) | 0.00 (0.00-0.00) | 0.00 (0.00-0.00) | 0.01 (0.01-0.01) | |
| 40-44 | 0.15 (0.11-0.21) | 0.18 (0.16-0.19) | 0.02 (0.01-0.02) | 0.00 (0.00-0.00) | 0.04 (0.03-0.05) | 0.02 (0.01-0.02) | 0.00 (0.00-0.00) | 0.04 (0.03-0.05) | 0.00 (0.00-0.00) | 0.00 (0.00-0.00) | 0.01 (0.01-0.02) | |
| 45-49 | 0.15 (0.11-0.21) | 0.15 (0.14-0.17) | 0.02 (0.02-0.03) | 0.00 (0.00-0.01) | 0.05 (0.04-0.06) | 0.02 (0.01-0.02) | 0.00 (0.00-0.00) | 0.05 (0.04-0.06) | 0.00 (0.00-0.00) | 0.00 (0.00-0.00) | 0.01 (0.01-0.02) | |
| 50-54 | 0.15 (0.11-0.20) | 0.13 (0.12-0.14) | 0.03 (0.03-0.04) | 0.01 (0.00-0.01) | 0.05 (0.04-0.06) | 0.02 (0.02-0.02) | 0.00 (0.00-0.00) | 0.06 (0.04-0.07) | 0.00 (0.00-0.00) | 0.00 (0.00-0.00) | 0.02 (0.01-0.02) | |
| 55-59 | 0.15 (0.10-0.20) | 0.12 (0.11-0.13) | 0.04 (0.04-0.05) | 0.01 (0.01-0.01) | 0.05 (0.04-0.06) | 0.02 (0.02-0.03) | 0.00 (0.00-0.00) | 0.06 (0.05-0.08) | 0.00 (0.00-0.00) | 0.00 (0.00-0.00) | 0.02 (0.01-0.02) | |
| 60-64 | 0.14 (0.10-0.20) | 0.10 (0.09-0.11) | 0.05 (0.05-0.06) | 0.01 (0.01-0.01) | 0.06 (0.05-0.07) | 0.02 (0.02-0.03) | 0.00 (0.00-0.00) | 0.07 (0.05-0.09) | 0.00 (0.00-0.00) | 0.01 (0.00-0.01) | 0.02 (0.02-0.02) | |
| 65-69 | 0.14 (0.10-0.19) | 0.09 (0.07-0.09) | 0.07 (0.06-0.08) | 0.01 (0.01-0.01) | 0.06 (0.05-0.07) | 0.02 (0.02-0.03) | 0.01 (0.00-0.01) | 0.08 (0.06-0.10) | 0.00 (0.00-0.00) | 0.01 (0.01-0.01) | 0.02 (0.02-0.03) | |
| 70-74 | 0.14 (0.10-0.19) | 0.07 (0.06-0.08) | 0.08 (0.07-0.09) | 0.01 (0.01-0.01) | 0.07 (0.05-0.08) | 0.02 (0.02-0.03) | 0.01 (0.01-0.01) | 0.09 (0.06-0.11) | 0.00 (0.00-0.00) | 0.01 (0.01-0.01) | 0.03 (0.02-0.03) | |
| 75+ | 0.14 (0.10-0.19) | 0.06 (0.05-0.07) | 0.09 (0.08-0.11) | 0.01 (0.01-0.01) | 0.06 (0.05-0.08) | 0.02 (0.01-0.02) | 0.01 (0.01-0.01) | 0.10 (0.07-0.12) | 0.00 (0.00-0.00) | 0.01 (0.01-0.01) | 0.03 (0.03-0.04) | |

**Supplementary table 6.** Age-specific health loss proportion (HeLP) and 95% confidence interval for persons diagnosed with opioid use disorder during 2000-2015. All estimates are adjusted for observed comorbidity from mental and substance use disorders and general medical conditions.

|  | Index disorder | Mental disorders | Circulatory diseases | Endocrine diseases | Pulmonary diseases | Gastrointestinal diseases | Urogenital diseases | Musculoskeletal diseases | Hematological diseases | Cancers | Neurological diseases | Other |
| --- | --- | --- | --- | --- | --- | --- | --- | --- | --- | --- | --- | --- |
| 15-19 | 0.43 (0.34-0.52) | 0.18 (0.12-0.24) | |  | 0.04 (0.02-0.07) | |  | 0.01 (0.00-0.01) | |  |  | 0.02 (0.00-0.03) |
| 20-24 | 0.42 (0.34-0.50) | 0.20 (0.17-0.22) | 0.00 (0.00-0.00) | | 0.03 (0.02-0.04) | 0.02 (0.01-0.02) | | 0.02 (0.01-0.03) | 0.00 (0.00-0.00) | | 0.01 (0.00-0.01) | 0.00 (0.00-0.00) |
| 25-29 | 0.41 (0.33-0.49) | 0.20 (0.18-0.22) | 0.00 (0.00-0.01) | 0.00 (0.00-0.00) | 0.03 (0.02-0.04) | 0.02 (0.02-0.03) | 0.00 (0.00-0.00) | 0.03 (0.03-0.04) | 0.00 (0.00-0.00) | 0.00 (0.00-0.00) | 0.01 (0.01-0.01) | |
| 30-34 | 0.40 (0.32-0.48) | 0.20 (0.18-0.22) | 0.01 (0.00-0.01) | 0.00 (0.00-0.00) | 0.03 (0.02-0.03) | 0.03 (0.03-0.04) | 0.00 (0.00-0.00) | 0.05 (0.03-0.06) | 0.00 (0.00-0.00) | 0.00 (0.00-0.00) | 0.01 (0.01-0.02) | |
| 35-39 | 0.38 (0.31-0.46) | 0.21 (0.18-0.23) | 0.01 (0.01-0.01) | 0.00 (0.00-0.00) | 0.03 (0.03-0.04) | 0.04 (0.03-0.05) | 0.00 (0.00-0.00) | 0.05 (0.04-0.07) | 0.00 (0.00-0.00) | 0.00 (0.00-0.00) | 0.02 (0.01-0.02) | |
| 40-44 | 0.37 (0.30-0.45) | 0.21 (0.19-0.23) | 0.01 (0.01-0.02) | 0.00 (0.00-0.00) | 0.04 (0.03-0.05) | 0.04 (0.03-0.05) | 0.00 (0.00-0.00) | 0.06 (0.05-0.08) | 0.00 (0.00-0.00) | 0.00 (0.00-0.00) | 0.02 (0.01-0.02) | |
| 45-49 | 0.37 (0.30-0.44) | 0.21 (0.18-0.22) | 0.02 (0.01-0.02) | 0.00 (0.00-0.01) | 0.04 (0.03-0.05) | 0.04 (0.03-0.05) | 0.00 (0.00-0.00) | 0.07 (0.05-0.08) | 0.00 (0.00-0.00) | 0.00 (0.00-0.00) | 0.02 (0.01-0.02) | |
| 50-54 | 0.36 (0.29-0.43) | 0.19 (0.17-0.21) | 0.02 (0.02-0.03) | 0.00 (0.00-0.01) | 0.05 (0.04-0.06) | 0.04 (0.03-0.05) | 0.00 (0.00-0.00) | 0.07 (0.06-0.09) | 0.00 (0.00-0.00) | 0.00 (0.00-0.00) | 0.02 (0.01-0.02) | |
| 55-59 | 0.35 (0.28-0.42) | 0.18 (0.16-0.20) | 0.03 (0.03-0.04) | 0.01 (0.01-0.01) | 0.05 (0.04-0.07) | 0.04 (0.03-0.05) | 0.00 (0.00-0.00) | 0.08 (0.06-0.10) | 0.00 (0.00-0.00) | 0.00 (0.00-0.00) | 0.02 (0.02-0.02) | |
| 60-64 | 0.35 (0.28-0.41) | 0.16 (0.13-0.18) | 0.05 (0.04-0.06) | 0.01 (0.01-0.01) | 0.07 (0.05-0.08) | 0.04 (0.03-0.04) | 0.00 (0.00-0.01) | 0.10 (0.07-0.12) | 0.00 (0.00-0.00) | 0.00 (0.00-0.00) | 0.02 (0.02-0.03) | |
| 65-69 | 0.34 (0.28-0.41) | 0.12 (0.10-0.15) | 0.06 (0.05-0.07) | 0.01 (0.01-0.02) | 0.08 (0.06-0.10) | 0.02 (0.02-0.03) | 0.01 (0.00-0.01) | 0.11 (0.08-0.13) | 0.00 (0.00-0.00) | 0.00 (0.00-0.01) | 0.02 (0.02-0.03) | |
| 70-74 | 0.34 (0.27-0.40) | 0.09 (0.07-0.12) | 0.07 (0.06-0.09) | 0.01 (0.01-0.02) | 0.08 (0.06-0.10) | 0.02 (0.02-0.04) | 0.01 (0.00-0.01) | 0.12 (0.09-0.14) | 0.00 (0.00-0.00) | 0.01 (0.00-0.01) | 0.03 (0.02-0.04) | |
| 75+ | 0.36 (0.28-0.42) | 0.05 (0.04-0.07) | 0.09 (0.07-0.11) | 0.01 (0.01-0.02) | 0.07 (0.05-0.09) | 0.02 (0.01-0.03) | 0.01 (0.00-0.01) | 0.13 (0.10-0.15) | 0.00 (0.00-0.00) | 0.01 (0.00-0.01) | 0.03 (0.02-0.04) | |

**Supplementary table 7.** Age-specific health loss proportion (HeLP) and 95% confidence interval for persons diagnosed with cannabis use disorder during 2000-2015. All estimates are adjusted for observed comorbidity from mental and substance use disorders and general medical conditions.

|  | Index disorder | Mental disorders | Circulatory diseases | Endocrine diseases | Pulmonary diseases | Gastrointestinal diseases | Urogenital diseases | Musculoskeletal diseases | Hematological diseases | Cancers | Neurological diseases | Other |
| --- | --- | --- | --- | --- | --- | --- | --- | --- | --- | --- | --- | --- |
| 10-14 | 0.07 (0.04-0.09) | 0.09 (0.07-0.12) | |  | 0.05 (0.03-0.07) | |  |  |  |  | 0.01 (0.00-0.02) | 0.01 (0.00-0.01) |
| 15-19 | 0.07 (0.04-0.09) | 0.16 (0.14-0.17) | 0.00 (0.00-0.00) | 0.00 (0.00-0.00) | 0.05 (0.04-0.06) | 0.00 (0.00-0.00) | | 0.00 (0.00-0.01) | 0.00 (0.00-0.00) | | 0.00 (0.00-0.00) | 0.00 (0.00-0.00) |
| 20-24 | 0.06 (0.04-0.09) | 0.25 (0.23-0.28) | 0.00 (0.00-0.00) | 0.00 (0.00-0.00) | 0.04 (0.03-0.05) | 0.00 (0.00-0.00) | 0.00 (0.00-0.00) | 0.01 (0.01-0.01) | 0.00 (0.00-0.00) | 0.00 (0.00-0.00) | 0.01 (0.00-0.01) | |
| 25-29 | 0.06 (0.04-0.09) | 0.32 (0.28-0.35) | 0.00 (0.00-0.00) | 0.00 (0.00-0.00) | 0.03 (0.03-0.04) | 0.00 (0.00-0.01) | 0.00 (0.00-0.00) | 0.02 (0.01-0.03) | 0.00 (0.00-0.00) | 0.00 (0.00-0.00) | 0.01 (0.01-0.01) | |
| 30-34 | 0.06 (0.04-0.08) | 0.35 (0.32-0.39) | 0.00 (0.00-0.01) | 0.00 (0.00-0.00) | 0.03 (0.02-0.04) | 0.01 (0.01-0.01) | 0.00 (0.00-0.00) | 0.03 (0.02-0.04) | 0.00 (0.00-0.00) | 0.00 (0.00-0.00) | 0.01 (0.01-0.01) | |
| 35-39 | 0.06 (0.04-0.08) | 0.38 (0.34-0.42) | 0.01 (0.01-0.01) | 0.00 (0.00-0.00) | 0.03 (0.03-0.04) | 0.01 (0.01-0.01) | 0.00 (0.00-0.00) | 0.04 (0.03-0.05) | 0.00 (0.00-0.00) | 0.00 (0.00-0.00) | 0.01 (0.01-0.01) | |
| 40-44 | 0.06 (0.04-0.08) | 0.39 (0.35-0.42) | 0.01 (0.01-0.01) | 0.00 (0.00-0.00) | 0.04 (0.03-0.05) | 0.02 (0.01-0.02) | 0.00 (0.00-0.00) | 0.04 (0.03-0.06) | 0.00 (0.00-0.00) | 0.00 (0.00-0.00) | 0.01 (0.01-0.01) | |
| 45-49 | 0.06 (0.04-0.08) | 0.38 (0.35-0.42) | 0.01 (0.01-0.02) | 0.00 (0.00-0.00) | 0.04 (0.03-0.05) | 0.02 (0.01-0.02) | 0.00 (0.00-0.00) | 0.05 (0.04-0.06) | 0.00 (0.00-0.00) | 0.00 (0.00-0.00) | 0.01 (0.01-0.01) | |
| 50-54 | 0.05 (0.04-0.08) | 0.39 (0.35-0.43) | 0.02 (0.02-0.02) | 0.00 (0.00-0.01) | 0.05 (0.04-0.06) | 0.02 (0.02-0.02) | 0.00 (0.00-0.00) | 0.05 (0.04-0.07) | 0.00 (0.00-0.00) | 0.00 (0.00-0.00) | 0.01 (0.01-0.02) | |
| 55-59 | 0.05 (0.04-0.07) | 0.39 (0.35-0.43) | 0.03 (0.02-0.04) | 0.01 (0.00-0.01) | 0.05 (0.04-0.06) | 0.02 (0.02-0.03) | 0.00 (0.00-0.00) | 0.06 (0.05-0.08) | 0.00 (0.00-0.00) | 0.00 (0.00-0.01) | 0.02 (0.01-0.02) | |
| 60-64 | 0.05 (0.04-0.07) | 0.35 (0.32-0.41) | 0.04 (0.03-0.05) | 0.01 (0.01-0.01) | 0.06 (0.04-0.07) | 0.02 (0.02-0.03) | 0.00 (0.00-0.00) | 0.07 (0.05-0.09) | 0.00 (0.00-0.00) | 0.01 (0.00-0.01) | 0.02 (0.01-0.03) | |
| 65-69 | 0.05 (0.03-0.07) | 0.36 (0.29-0.42) | 0.05 (0.03-0.06) | 0.01 (0.00-0.01) | 0.06 (0.05-0.10) | 0.02 (0.01-0.03) | 0.00 (0.00-0.01) | 0.08 (0.06-0.10) | 0.00 (0.00-0.00) | 0.01 (0.00-0.01) | 0.02 (0.01-0.03) | |
| 70-74 | 0.05 (0.03-0.07) | 0.33 (0.22-0.43) | 0.05 (0.03-0.08) | 0.01 (0.00-0.01) | 0.06 (0.05-0.12) | 0.04 (0.01-0.06) | 0.01 (0.00-0.02) | 0.08 (0.05-0.11) | 0.00 (0.00-0.01) | 0.01 (0.00-0.01) | 0.03 (0.02-0.05) | |
| 75+ | 0.05 (0.03-0.07) | 0.13 (0.03-0.25) | 0.09 (0.04-0.13) | | 0.05 (0.01-0.16) | |  | 0.12 (0.08-0.16) | |  | 0.06 (0.03-0.08) | 0.05 (0.02-0.08) |

**Supplementary table 8.** Age-specific health loss proportion (HeLP) and 95% confidence interval for persons diagnosed with cocaine use disorder during 2000-2015. All estimates are adjusted for observed comorbidity from mental and substance use disorders and general medical conditions.

|  | Index disorder | Mental disorders | Circulatory diseases | Endocrine diseases | Pulmonary diseases | Gastrointestinal diseases | Urogenital diseases | Musculoskeletal diseases | Hematological diseases | Cancers | Neurological diseases | Other |
| --- | --- | --- | --- | --- | --- | --- | --- | --- | --- | --- | --- | --- |
| 10-14 |  |  |  |  |  |  |  |  |  |  |  |  |
| 15-19 | 0.24 (0.18-0.31) | 0.23 (0.18-0.28) | |  | 0.04 (0.03-0.06) | |  | 0.01 (0.00-0.02) | |  | 0.00 (0.00-0.01) | 0.00 (0.00-0.00) |
| 20-24 | 0.23 (0.17-0.29) | 0.29 (0.27-0.34) | 0.00 (0.00-0.00) | 0.00 (0.00-0.00) | 0.04 (0.03-0.05) | 0.00 (0.00-0.01) | | 0.01 (0.01-0.01) | |  | 0.01 (0.00-0.01) | 0.00 (0.00-0.00) |
| 25-29 | 0.22 (0.17-0.28) | 0.31 (0.28-0.35) | 0.00 (0.00-0.01) | 0.00 (0.00-0.00) | 0.04 (0.03-0.05) | 0.01 (0.00-0.01) | | 0.02 (0.02-0.03) | 0.00 (0.00-0.00) | | 0.01 (0.01-0.01) | 0.00 (0.00-0.00) |
| 30-34 | 0.22 (0.16-0.27) | 0.32 (0.29-0.35) | 0.01 (0.00-0.01) | 0.00 (0.00-0.00) | 0.03 (0.03-0.04) | 0.01 (0.01-0.02) | 0.00 (0.00-0.00) | 0.03 (0.02-0.04) | 0.00 (0.00-0.00) | 0.00 (0.00-0.00) | 0.01 (0.01-0.01) | |
| 35-39 | 0.21 (0.15-0.26) | 0.34 (0.31-0.38) | 0.01 (0.01-0.01) | 0.00 (0.00-0.00) | 0.03 (0.03-0.05) | 0.02 (0.02-0.03) | 0.00 (0.00-0.00) | 0.04 (0.03-0.05) | 0.00 (0.00-0.00) | 0.00 (0.00-0.00) | 0.01 (0.01-0.01) | |
| 40-44 | 0.20 (0.15-0.25) | 0.37 (0.32-0.40) | 0.01 (0.01-0.02) | 0.00 (0.00-0.00) | 0.04 (0.03-0.05) | 0.03 (0.02-0.04) | 0.00 (0.00-0.00) | 0.05 (0.03-0.06) | 0.00 (0.00-0.00) | 0.00 (0.00-0.00) | 0.01 (0.01-0.02) | |
| 45-49 | 0.20 (0.15-0.24) | 0.38 (0.34-0.42) | 0.02 (0.01-0.02) | 0.00 (0.00-0.00) | 0.03 (0.03-0.05) | 0.03 (0.02-0.04) | 0.00 (0.00-0.00) | 0.05 (0.03-0.06) | 0.00 (0.00-0.00) | 0.00 (0.00-0.00) | 0.01 (0.01-0.02) | |
| 50-54 | 0.19 (0.14-0.24) | 0.40 (0.33-0.44) | 0.02 (0.01-0.03) | 0.00 (0.00-0.00) | 0.04 (0.03-0.06) | 0.03 (0.02-0.04) | | 0.06 (0.04-0.07) | 0.00 (0.00-0.00) | | 0.01 (0.01-0.02) | 0.00 (0.00-0.00) |
| 55-59 | 0.18 (0.13-0.23) | 0.40 (0.33-0.47) | 0.03 (0.02-0.04) | 0.01 (0.00-0.01) | 0.05 (0.04-0.07) | 0.03 (0.01-0.04) | | 0.06 (0.04-0.08) | 0.00 (0.00-0.01) | | 0.01 (0.00-0.02) | 0.00 (0.00-0.00) |
| 60-64 | 0.18 (0.13-0.23) | 0.41 (0.25-0.47) | 0.02 (0.01-0.04) | 0.01 (0.00-0.03) | 0.06 (0.04-0.11) | 0.02 (0.01-0.04) | | 0.06 (0.04-0.10) | |  | 0.00 (0.00-0.01) | 0.01 (0.00-0.02) |
| 65-69 | 0.18 (0.13-0.23) | 0.43 (0.13-0.56) | 0.03 (0.01-0.07) | | 0.04 (0.02-0.14) | |  | 0.06 (0.02-0.12) | |  |  | 0.05 (0.03-0.12) |
| 70-74 |  |  |  |  |  |  |  |  |  |  |  |  |
| 75+ |  |  |  |  |  |  |  |  |  |  |  |  |

**Supplementary table 9.** Age-specific health loss proportion (HeLP) and 95% confidence interval for persons diagnosed with amphetamine use disorder during 2000-2015. All estimates are adjusted for observed comorbidity from mental and substance use disorders and general medical conditions.

|  | Index disorder | Mental disorders | Circulatory diseases | Endocrine diseases | Pulmonary diseases | Gastrointestinal diseases | Urogenital diseases | Musculoskeletal diseases | Hematological diseases | Cancers | Neurological diseases | Other |
| --- | --- | --- | --- | --- | --- | --- | --- | --- | --- | --- | --- | --- |
| 10-14 |  |  |  |  |  |  |  |  |  |  |  |  |
| 15-19 | 0.27 (0.20-0.33) | 0.17 (0.14-0.20) | |  | 0.04 (0.03-0.06) | 0.00 (0.00-0.00) | | 0.01 (0.00-0.01) | |  | 0.01 (0.00-0.01) | 0.00 (0.00-0.00) |
| 20-24 | 0.25 (0.19-0.31) | 0.25 (0.22-0.27) | 0.00 (0.00-0.00) | 0.00 (0.00-0.00) | 0.04 (0.03-0.05) | 0.00 (0.00-0.00) | | 0.01 (0.01-0.02) | |  | 0.01 (0.00-0.01) | 0.00 (0.00-0.00) |
| 25-29 | 0.24 (0.18-0.29) | 0.30 (0.27-0.33) | 0.00 (0.00-0.00) | 0.00 (0.00-0.00) | 0.04 (0.03-0.05) | 0.00 (0.00-0.01) | | 0.03 (0.02-0.03) | 0.00 (0.00-0.00) | | 0.01 (0.01-0.01) | 0.00 (0.00-0.00) |
| 30-34 | 0.23 (0.17-0.28) | 0.34 (0.30-0.36) | 0.00 (0.00-0.01) | 0.00 (0.00-0.00) | 0.03 (0.02-0.04) | 0.01 (0.01-0.01) | 0.00 (0.00-0.00) | 0.04 (0.03-0.05) | 0.00 (0.00-0.00) | 0.00 (0.00-0.00) | 0.01 (0.01-0.01) | |
| 35-39 | 0.21 (0.16-0.26) | 0.38 (0.33-0.41) | 0.01 (0.01-0.01) | 0.00 (0.00-0.00) | 0.04 (0.03-0.05) | 0.02 (0.01-0.02) | 0.00 (0.00-0.00) | 0.05 (0.03-0.06) | 0.00 (0.00-0.00) | 0.00 (0.00-0.00) | 0.01 (0.01-0.02) | |
| 40-44 | 0.21 (0.16-0.26) | 0.38 (0.34-0.41) | 0.01 (0.01-0.01) | 0.00 (0.00-0.00) | 0.04 (0.03-0.05) | 0.02 (0.02-0.03) | 0.00 (0.00-0.00) | 0.05 (0.03-0.06) | 0.00 (0.00-0.00) | 0.00 (0.00-0.00) | 0.01 (0.01-0.02) | |
| 45-49 | 0.21 (0.16-0.25) | 0.37 (0.32-0.41) | 0.02 (0.01-0.02) | 0.00 (0.00-0.01) | 0.04 (0.03-0.05) | 0.03 (0.02-0.04) | 0.00 (0.00-0.00) | 0.05 (0.04-0.07) | 0.00 (0.00-0.00) | 0.00 (0.00-0.00) | 0.01 (0.01-0.02) | |
| 50-54 | 0.20 (0.15-0.24) | 0.40 (0.35-0.45) | 0.02 (0.02-0.03) | 0.00 (0.00-0.01) | 0.05 (0.03-0.06) | 0.03 (0.02-0.04) | | 0.06 (0.04-0.08) | 0.00 (0.00-0.00) | | 0.01 (0.00-0.01) | 0.00 (0.00-0.00) |
| 55-59 | 0.19 (0.15-0.23) | 0.42 (0.33-0.47) | 0.03 (0.02-0.04) | 0.01 (0.00-0.01) | 0.06 (0.03-0.07) | 0.02 (0.02-0.04) | | 0.07 (0.05-0.09) | 0.00 (0.00-0.00) | | 0.01 (0.00-0.01) | 0.00 (0.00-0.01) |
| 60-64 | 0.18 (0.14-0.23) | 0.41 (0.26-0.47) | 0.03 (0.02-0.05) | 0.01 (0.00-0.02) | 0.05 (0.03-0.09) | 0.03 (0.02-0.05) | | 0.07 (0.05-0.11) | 0.00 (0.00-0.00) | | 0.00 (0.00-0.03) | 0.00 (0.00-0.01) |
| 65-69 | 0.20 (0.15-0.25) | 0.34 (0.18-0.49) | 0.05 (0.02-0.08) | | 0.03 (0.01-0.09) | 0.04 (0.00-0.06) | | 0.05 (0.03-0.11) | |  |  | 0.03 (0.01-0.06) |
| 70-74 |  |  |  |  |  |  |  |  |  |  |  |  |
| 75+ | 0.21 (0.16-0.27) | |  |  |  |  |  | 0.15 (0.09-0.20) | |  |  | 0.29 (0.18-0.49) |

**Supplementary table 10.** Age-specific health loss proportion (HeLP) and 95% confidence interval for persons diagnosed with other drug use disorders during 2000-2015. All estimates are adjusted for observed comorbidity from mental and substance use disorders and general medical conditions.

|  | Index disorder | Mental disorders | Circulatory diseases | Endocrine diseases | Pulmonary diseases | Gastrointestinal diseases | Urogenital diseases | Musculoskeletal diseases | Hematological diseases | Cancers | Neurological diseases | Other |
| --- | --- | --- | --- | --- | --- | --- | --- | --- | --- | --- | --- | --- |
| 10-14 | 0.11 (0.07-0.14) | |  |  |  |  |  |  |  |  |  | 0.21 (0.06-0.43) |
| 15-19 | 0.10 (0.07-0.13) | 0.30 (0.20-0.31) | |  | 0.04 (0.03-0.08) | |  | 0.01 (0.00-0.02) | |  | 0.00 (0.00-0.02) | 0.01 (0.00-0.01) |
| 20-24 | 0.09 (0.06-0.12) | 0.43 (0.36-0.45) | 0.00 (0.00-0.00) | | 0.04 (0.02-0.05) | 0.00 (0.00-0.01) | | 0.02 (0.01-0.03) | |  | 0.01 (0.00-0.01) | 0.00 (0.00-0.00) |
| 25-29 | 0.09 (0.06-0.11) | 0.42 (0.38-0.46) | 0.00 (0.00-0.01) | 0.00 (0.00-0.00) | 0.04 (0.03-0.05) | 0.01 (0.01-0.01) | | 0.04 (0.03-0.05) | 0.00 (0.00-0.00) | | 0.02 (0.01-0.02) | 0.00 (0.00-0.00) |
| 30-34 | 0.08 (0.06-0.11) | 0.46 (0.42-0.50) | 0.01 (0.00-0.01) | 0.00 (0.00-0.00) | 0.03 (0.02-0.04) | 0.02 (0.01-0.02) | | 0.05 (0.03-0.06) | 0.00 (0.00-0.00) | | 0.01 (0.01-0.02) | 0.00 (0.00-0.00) |
| 35-39 | 0.08 (0.06-0.11) | 0.43 (0.40-0.47) | 0.01 (0.01-0.02) | 0.00 (0.00-0.00) | 0.05 (0.03-0.05) | 0.02 (0.01-0.02) | 0.00 (0.00-0.00) | 0.06 (0.04-0.07) | 0.00 (0.00-0.00) | 0.00 (0.00-0.00) | 0.02 (0.01-0.02) | |
| 40-44 | 0.08 (0.06-0.11) | 0.41 (0.36-0.43) | 0.02 (0.01-0.02) | 0.00 (0.00-0.01) | 0.06 (0.05-0.07) | 0.02 (0.02-0.03) | 0.00 (0.00-0.00) | 0.06 (0.04-0.08) | 0.00 (0.00-0.00) | 0.00 (0.00-0.00) | 0.02 (0.01-0.02) | |
| 45-49 | 0.08 (0.06-0.11) | 0.36 (0.32-0.39) | 0.03 (0.02-0.03) | 0.01 (0.00-0.01) | 0.07 (0.05-0.08) | 0.02 (0.02-0.03) | 0.00 (0.00-0.00) | 0.07 (0.05-0.09) | 0.00 (0.00-0.00) | 0.00 (0.00-0.00) | 0.02 (0.01-0.02) | |
| 50-54 | 0.08 (0.06-0.11) | 0.32 (0.29-0.36) | 0.03 (0.03-0.04) | 0.01 (0.01-0.01) | 0.07 (0.05-0.08) | 0.02 (0.01-0.02) | 0.00 (0.00-0.00) | 0.08 (0.06-0.10) | 0.00 (0.00-0.00) | 0.00 (0.00-0.00) | 0.02 (0.01-0.02) | |
| 55-59 | 0.08 (0.06-0.11) | 0.28 (0.26-0.32) | 0.04 (0.03-0.05) | 0.01 (0.01-0.01) | 0.07 (0.06-0.08) | 0.02 (0.01-0.02) | 0.00 (0.00-0.00) | 0.08 (0.06-0.10) | 0.00 (0.00-0.00) | 0.00 (0.00-0.00) | 0.02 (0.01-0.02) | |
| 60-64 | 0.08 (0.06-0.11) | 0.26 (0.22-0.29) | 0.05 (0.04-0.06) | 0.01 (0.01-0.01) | 0.08 (0.06-0.09) | 0.02 (0.01-0.02) | 0.00 (0.00-0.00) | 0.09 (0.07-0.11) | 0.00 (0.00-0.00) | 0.00 (0.00-0.01) | 0.02 (0.01-0.02) | |
| 65-69 | 0.08 (0.06-0.11) | 0.24 (0.21-0.28) | 0.06 (0.05-0.08) | 0.01 (0.01-0.01) | 0.07 (0.05-0.09) | 0.02 (0.01-0.02) | 0.00 (0.00-0.01) | 0.09 (0.07-0.11) | 0.00 (0.00-0.00) | 0.01 (0.00-0.01) | 0.02 (0.01-0.03) | |
| 70-74 | 0.08 (0.06-0.11) | 0.19 (0.17-0.24) | 0.06 (0.05-0.08) | 0.01 (0.01-0.02) | 0.07 (0.06-0.10) | 0.02 (0.01-0.03) | 0.00 (0.00-0.01) | 0.10 (0.07-0.12) | 0.00 (0.00-0.00) | 0.01 (0.01-0.02) | 0.02 (0.02-0.03) | |
| 75+ | 0.09 (0.06-0.11) | 0.16 (0.13-0.19) | 0.07 (0.06-0.09) | 0.01 (0.01-0.01) | 0.06 (0.05-0.09) | 0.01 (0.01-0.01) | 0.00 (0.00-0.01) | 0.11 (0.08-0.13) | 0.00 (0.00-0.00) | 0.01 (0.01-0.01) | 0.03 (0.02-0.04) | |

**Supplementary table 11.** Age-specific health loss proportion (HeLP) and 95% confidence interval for persons diagnosed with schizophrenia during 2000-2015. All estimates are adjusted for observed comorbidity from mental and substance use disorders and general medical conditions.

|  | Index disorder | Mental disorders | Circulatory diseases | Endocrine diseases | Pulmonary diseases | Gastrointestinal diseases | Urogenital diseases | Musculoskeletal diseases | Hematological diseases | Cancers | Neurological diseases | Other |
| --- | --- | --- | --- | --- | --- | --- | --- | --- | --- | --- | --- | --- |
| 10-14 | 0.61 (0.51-0.69) | 0.11 (0.09-0.13) | |  | 0.04 (0.03-0.05) | |  | 0.00 (0.00-0.00) | |  | 0.00 (0.00-0.01) | 0.00 (0.00-0.00) |
| 15-19 | 0.61 (0.52-0.70) | 0.09 (0.08-0.10) | 0.00 (0.00-0.00) | 0.00 (0.00-0.00) | 0.04 (0.03-0.05) | 0.00 (0.00-0.00) | 0.00 (0.00-0.00) | 0.01 (0.00-0.01) | 0.00 (0.00-0.00) | 0.00 (0.00-0.00) | 0.01 (0.00-0.01) | |
| 20-24 | 0.61 (0.51-0.70) | 0.09 (0.08-0.10) | 0.00 (0.00-0.00) | 0.00 (0.00-0.00) | 0.04 (0.03-0.05) | 0.00 (0.00-0.00) | 0.00 (0.00-0.00) | 0.01 (0.01-0.01) | 0.00 (0.00-0.00) | 0.00 (0.00-0.00) | 0.01 (0.01-0.01) | |
| 25-29 | 0.60 (0.51-0.69) | 0.09 (0.08-0.10) | 0.00 (0.00-0.00) | 0.00 (0.00-0.00) | 0.04 (0.03-0.04) | 0.00 (0.00-0.00) | 0.00 (0.00-0.00) | 0.02 (0.01-0.02) | 0.00 (0.00-0.00) | 0.00 (0.00-0.00) | 0.01 (0.01-0.01) | |
| 30-34 | 0.60 (0.51-0.69) | 0.09 (0.08-0.10) | 0.01 (0.00-0.01) | 0.00 (0.00-0.00) | 0.03 (0.03-0.04) | 0.00 (0.00-0.01) | 0.00 (0.00-0.00) | 0.02 (0.02-0.02) | 0.00 (0.00-0.00) | 0.00 (0.00-0.00) | 0.01 (0.01-0.01) | |
| 35-39 | 0.60 (0.51-0.69) | 0.08 (0.07-0.09) | 0.01 (0.01-0.01) | 0.00 (0.00-0.01) | 0.03 (0.03-0.04) | 0.01 (0.00-0.01) | 0.00 (0.00-0.00) | 0.02 (0.02-0.03) | 0.00 (0.00-0.00) | 0.00 (0.00-0.00) | 0.01 (0.01-0.01) | |
| 40-44 | 0.60 (0.51-0.68) | 0.07 (0.06-0.08) | 0.01 (0.01-0.01) | 0.01 (0.00-0.01) | 0.04 (0.03-0.04) | 0.01 (0.00-0.01) | 0.00 (0.00-0.00) | 0.03 (0.02-0.04) | 0.00 (0.00-0.00) | 0.00 (0.00-0.00) | 0.01 (0.01-0.01) | |
| 45-49 | 0.59 (0.50-0.68) | 0.07 (0.06-0.07) | 0.02 (0.01-0.02) | 0.01 (0.01-0.01) | 0.04 (0.03-0.04) | 0.01 (0.01-0.01) | 0.00 (0.00-0.00) | 0.03 (0.03-0.04) | 0.00 (0.00-0.00) | 0.00 (0.00-0.00) | 0.01 (0.01-0.01) | |
| 50-54 | 0.59 (0.50-0.67) | 0.06 (0.05-0.06) | 0.02 (0.02-0.02) | 0.01 (0.01-0.01) | 0.04 (0.03-0.05) | 0.01 (0.01-0.01) | 0.00 (0.00-0.00) | 0.04 (0.03-0.05) | 0.00 (0.00-0.00) | 0.00 (0.00-0.00) | 0.01 (0.01-0.01) | |
| 55-59 | 0.58 (0.49-0.67) | 0.05 (0.04-0.05) | 0.03 (0.02-0.03) | 0.01 (0.01-0.01) | 0.04 (0.03-0.05) | 0.01 (0.01-0.01) | 0.00 (0.00-0.00) | 0.04 (0.03-0.05) | 0.00 (0.00-0.00) | 0.00 (0.00-0.00) | 0.01 (0.01-0.01) | |
| 60-64 | 0.58 (0.49-0.66) | 0.04 (0.03-0.04) | 0.03 (0.03-0.04) | 0.01 (0.01-0.02) | 0.04 (0.03-0.05) | 0.01 (0.01-0.01) | 0.00 (0.00-0.00) | 0.05 (0.04-0.06) | 0.00 (0.00-0.00) | 0.00 (0.00-0.00) | 0.01 (0.01-0.02) | |
| 65-69 | 0.57 (0.48-0.65) | 0.03 (0.03-0.04) | 0.04 (0.04-0.05) | 0.01 (0.01-0.02) | 0.04 (0.03-0.05) | 0.01 (0.01-0.01) | 0.00 (0.00-0.00) | 0.06 (0.04-0.07) | 0.00 (0.00-0.00) | 0.01 (0.00-0.01) | 0.02 (0.01-0.02) | |
| 70-74 | 0.56 (0.48-0.64) | 0.02 (0.02-0.03) | 0.05 (0.04-0.06) | 0.01 (0.01-0.02) | 0.04 (0.03-0.05) | 0.01 (0.01-0.01) | 0.00 (0.00-0.01) | 0.06 (0.05-0.08) | 0.00 (0.00-0.00) | 0.01 (0.01-0.01) | 0.02 (0.01-0.02) | |
| 75+ | 0.55 (0.47-0.63) | 0.02 (0.01-0.02) | 0.06 (0.05-0.07) | 0.01 (0.01-0.02) | 0.04 (0.03-0.04) | 0.01 (0.00-0.01) | 0.00 (0.00-0.00) | 0.08 (0.06-0.09) | 0.00 (0.00-0.00) | 0.01 (0.01-0.01) | 0.02 (0.02-0.03) | |

**Supplementary table 12.** Age-specific health loss proportion (HeLP) and 95% confidence interval for persons diagnosed with bipolar disorder during 2000-2015. All estimates are adjusted for observed comorbidity from mental and substance use disorders and general medical conditions.

|  | Index disorder | Mental disorders | Circulatory diseases | Endocrine diseases | Pulmonary diseases | Gastrointestinal diseases | Urogenital diseases | Musculoskeletal diseases | Hematological diseases | Cancers | Neurological diseases | Other |
| --- | --- | --- | --- | --- | --- | --- | --- | --- | --- | --- | --- | --- |
| 10-14 | 0.20 (0.15-0.25) | 0.12 (0.08-0.16) | |  | 0.05 (0.02-0.07) | |  |  |  |  | 0.01 (0.00-0.03) | 0.00 (0.00-0.01) |
| 15-19 | 0.20 (0.15-0.25) | 0.12 (0.11-0.14) | 0.00 (0.00-0.00) | 0.00 (0.00-0.00) | 0.04 (0.03-0.06) | 0.00 (0.00-0.00) | | 0.01 (0.00-0.01) | 0.00 (0.00-0.00) | | 0.01 (0.00-0.01) | 0.00 (0.00-0.00) |
| 20-24 | 0.19 (0.14-0.25) | 0.14 (0.13-0.16) | 0.00 (0.00-0.00) | 0.00 (0.00-0.00) | 0.04 (0.03-0.05) | 0.00 (0.00-0.00) | 0.00 (0.00-0.00) | 0.01 (0.01-0.02) | 0.00 (0.00-0.00) | 0.00 (0.00-0.00) | 0.01 (0.01-0.01) | |
| 25-29 | 0.19 (0.14-0.25) | 0.15 (0.13-0.17) | 0.00 (0.00-0.00) | 0.00 (0.00-0.00) | 0.04 (0.03-0.06) | 0.00 (0.00-0.00) | 0.00 (0.00-0.00) | 0.02 (0.01-0.03) | 0.00 (0.00-0.00) | 0.00 (0.00-0.00) | 0.01 (0.01-0.01) | |
| 30-34 | 0.19 (0.14-0.25) | 0.15 (0.13-0.16) | 0.01 (0.00-0.01) | 0.00 (0.00-0.01) | 0.04 (0.03-0.06) | 0.00 (0.00-0.01) | 0.00 (0.00-0.00) | 0.03 (0.02-0.03) | 0.00 (0.00-0.00) | 0.00 (0.00-0.00) | 0.01 (0.01-0.01) | |
| 35-39 | 0.19 (0.14-0.24) | 0.14 (0.13-0.16) | 0.01 (0.01-0.01) | 0.01 (0.00-0.01) | 0.05 (0.04-0.06) | 0.01 (0.00-0.01) | 0.00 (0.00-0.00) | 0.03 (0.02-0.04) | 0.00 (0.00-0.00) | 0.00 (0.00-0.00) | 0.01 (0.01-0.01) | |
| 40-44 | 0.19 (0.14-0.24) | 0.13 (0.12-0.15) | 0.01 (0.01-0.02) | 0.01 (0.01-0.01) | 0.05 (0.04-0.06) | 0.01 (0.00-0.01) | 0.00 (0.00-0.00) | 0.04 (0.03-0.05) | 0.00 (0.00-0.00) | 0.00 (0.00-0.00) | 0.01 (0.01-0.01) | |
| 45-49 | 0.18 (0.14-0.24) | 0.12 (0.11-0.14) | 0.02 (0.02-0.02) | 0.01 (0.01-0.01) | 0.05 (0.04-0.06) | 0.01 (0.00-0.01) | 0.00 (0.00-0.00) | 0.04 (0.03-0.05) | 0.00 (0.00-0.00) | 0.00 (0.00-0.00) | 0.01 (0.01-0.01) | |
| 50-54 | 0.18 (0.14-0.24) | 0.11 (0.10-0.12) | 0.03 (0.02-0.03) | 0.01 (0.01-0.01) | 0.05 (0.04-0.06) | 0.01 (0.01-0.01) | 0.00 (0.00-0.00) | 0.05 (0.04-0.06) | 0.00 (0.00-0.00) | 0.00 (0.00-0.00) | 0.01 (0.01-0.02) | |
| 55-59 | 0.18 (0.14-0.23) | 0.10 (0.09-0.11) | 0.04 (0.03-0.04) | 0.01 (0.01-0.02) | 0.05 (0.04-0.06) | 0.01 (0.01-0.01) | 0.00 (0.00-0.00) | 0.05 (0.04-0.07) | 0.00 (0.00-0.00) | 0.00 (0.00-0.00) | 0.01 (0.01-0.02) | |
| 60-64 | 0.18 (0.13-0.23) | 0.08 (0.07-0.09) | 0.05 (0.04-0.05) | 0.02 (0.01-0.02) | 0.06 (0.04-0.07) | 0.01 (0.01-0.01) | 0.00 (0.00-0.00) | 0.06 (0.05-0.08) | 0.00 (0.00-0.00) | 0.01 (0.01-0.01) | 0.02 (0.01-0.02) | |
| 65-69 | 0.18 (0.13-0.23) | 0.06 (0.06-0.07) | 0.06 (0.05-0.07) | 0.02 (0.01-0.02) | 0.06 (0.05-0.07) | 0.01 (0.01-0.01) | 0.00 (0.00-0.01) | 0.07 (0.06-0.09) | 0.00 (0.00-0.00) | 0.01 (0.01-0.01) | 0.02 (0.02-0.02) | |
| 70-74 | 0.17 (0.13-0.22) | 0.05 (0.04-0.06) | 0.07 (0.06-0.08) | 0.02 (0.01-0.02) | 0.06 (0.05-0.07) | 0.01 (0.01-0.01) | 0.01 (0.00-0.01) | 0.08 (0.06-0.10) | 0.00 (0.00-0.00) | 0.01 (0.01-0.01) | 0.02 (0.02-0.03) | |
| 75+ | 0.17 (0.13-0.22) | 0.04 (0.03-0.04) | 0.08 (0.07-0.09) | 0.02 (0.01-0.02) | 0.05 (0.04-0.07) | 0.01 (0.01-0.01) | 0.01 (0.00-0.01) | 0.09 (0.07-0.11) | 0.00 (0.00-0.00) | 0.01 (0.01-0.01) | 0.03 (0.03-0.04) | |

**Supplementary table 13.** Age-specific health loss proportion (HeLP) and 95% confidence interval for persons diagnosed with dysthymia during 2000-2015. All estimates are adjusted for observed comorbidity from mental and substance use disorders and general medical conditions.

|  | Index disorder | Mental disorders | Circulatory diseases | Endocrine diseases | Pulmonary diseases | Gastrointestinal diseases | Urogenital diseases | Musculoskeletal diseases | Hematological diseases | Cancers | Neurological diseases | Other |
| --- | --- | --- | --- | --- | --- | --- | --- | --- | --- | --- | --- | --- |
| 10-14 | 0.13 (0.10-0.18) | 0.08 (0.06-0.11) | |  | 0.06 (0.04-0.09) | |  |  |  |  |  | 0.00 (0.00-0.01) |
| 15-19 | 0.13 (0.10-0.17) | 0.12 (0.10-0.14) | 0.00 (0.00-0.00) | 0.00 (0.00-0.00) | 0.05 (0.04-0.06) | |  | 0.00 (0.00-0.01) | |  | 0.00 (0.00-0.00) | 0.00 (0.00-0.00) |
| 20-24 | 0.13 (0.10-0.17) | 0.13 (0.12-0.16) | 0.00 (0.00-0.00) | 0.00 (0.00-0.00) | 0.05 (0.03-0.06) | 0.00 (0.00-0.00) | | 0.02 (0.01-0.02) | 0.00 (0.00-0.00) | | 0.01 (0.00-0.01) | 0.00 (0.00-0.00) |
| 25-29 | 0.13 (0.10-0.17) | 0.15 (0.13-0.17) | 0.00 (0.00-0.00) | 0.00 (0.00-0.00) | 0.04 (0.03-0.06) | 0.00 (0.00-0.01) | 0.00 (0.00-0.00) | 0.02 (0.01-0.03) | 0.00 (0.00-0.00) | 0.00 (0.00-0.00) | 0.01 (0.00-0.01) | |
| 30-34 | 0.13 (0.09-0.16) | 0.15 (0.12-0.17) | 0.01 (0.00-0.01) | 0.00 (0.00-0.01) | 0.04 (0.03-0.06) | 0.00 (0.00-0.01) | 0.00 (0.00-0.00) | 0.03 (0.02-0.04) | 0.00 (0.00-0.00) | 0.00 (0.00-0.00) | 0.01 (0.01-0.01) | |
| 35-39 | 0.12 (0.09-0.16) | 0.14 (0.12-0.16) | 0.01 (0.01-0.01) | 0.01 (0.00-0.01) | 0.05 (0.04-0.07) | 0.01 (0.00-0.01) | 0.00 (0.00-0.00) | 0.04 (0.03-0.05) | 0.00 (0.00-0.00) | 0.00 (0.00-0.00) | 0.01 (0.01-0.01) | |
| 40-44 | 0.12 (0.09-0.16) | 0.12 (0.11-0.15) | 0.01 (0.01-0.02) | 0.01 (0.01-0.01) | 0.06 (0.05-0.08) | 0.01 (0.00-0.01) | 0.00 (0.00-0.00) | 0.05 (0.04-0.06) | 0.00 (0.00-0.00) | 0.00 (0.00-0.00) | 0.01 (0.01-0.02) | |
| 45-49 | 0.12 (0.09-0.16) | 0.13 (0.11-0.14) | 0.02 (0.02-0.03) | 0.01 (0.01-0.01) | 0.07 (0.05-0.08) | 0.01 (0.01-0.01) | 0.00 (0.00-0.00) | 0.06 (0.04-0.07) | 0.00 (0.00-0.00) | 0.00 (0.00-0.00) | 0.01 (0.01-0.02) | |
| 50-54 | 0.12 (0.09-0.16) | 0.12 (0.10-0.14) | 0.03 (0.02-0.03) | 0.01 (0.01-0.01) | 0.07 (0.05-0.08) | 0.01 (0.01-0.01) | 0.00 (0.00-0.00) | 0.07 (0.05-0.08) | 0.00 (0.00-0.00) | 0.00 (0.00-0.00) | 0.01 (0.01-0.02) | |
| 55-59 | 0.12 (0.09-0.15) | 0.12 (0.10-0.13) | 0.04 (0.03-0.05) | 0.01 (0.01-0.02) | 0.07 (0.05-0.08) | 0.01 (0.01-0.01) | 0.00 (0.00-0.00) | 0.07 (0.05-0.09) | 0.00 (0.00-0.00) | 0.00 (0.00-0.00) | 0.02 (0.01-0.02) | |
| 60-64 | 0.11 (0.09-0.15) | 0.10 (0.09-0.12) | 0.05 (0.04-0.06) | 0.01 (0.01-0.02) | 0.07 (0.06-0.09) | 0.01 (0.01-0.01) | 0.00 (0.00-0.00) | 0.08 (0.06-0.10) | 0.00 (0.00-0.00) | 0.01 (0.00-0.01) | 0.02 (0.01-0.02) | |
| 65-69 | 0.11 (0.08-0.15) | 0.10 (0.08-0.11) | 0.06 (0.05-0.07) | 0.01 (0.01-0.02) | 0.07 (0.06-0.09) | 0.01 (0.01-0.01) | 0.00 (0.00-0.01) | 0.09 (0.07-0.11) | 0.00 (0.00-0.00) | 0.01 (0.00-0.01) | 0.02 (0.02-0.03) | |
| 70-74 | 0.11 (0.08-0.15) | 0.09 (0.07-0.10) | 0.08 (0.06-0.09) | 0.02 (0.01-0.02) | 0.08 (0.06-0.09) | 0.01 (0.01-0.02) | 0.00 (0.00-0.01) | 0.10 (0.07-0.12) | 0.00 (0.00-0.00) | 0.01 (0.00-0.01) | 0.03 (0.02-0.03) | |
| 75+ | 0.11 (0.08-0.15) | 0.05 (0.04-0.07) | 0.08 (0.07-0.10) | 0.01 (0.01-0.02) | 0.06 (0.05-0.08) | 0.01 (0.01-0.01) | 0.01 (0.00-0.01) | 0.11 (0.09-0.14) | 0.00 (0.00-0.00) | 0.01 (0.01-0.01) | 0.03 (0.03-0.04) | |

**Supplementary table 14.** Age-specific health loss proportion (HeLP) and 95% confidence interval for persons diagnosed with major depressive disorder during 2000-2015. All estimates are adjusted for observed comorbidity from mental and substance use disorders and general medical conditions.

|  | Index disorder | Mental disorders | Circulatory diseases | Endocrine diseases | Pulmonary diseases | Gastrointestinal diseases | Urogenital diseases | Musculoskeletal diseases | Hematological diseases | Cancers | Neurological diseases | Other |
| --- | --- | --- | --- | --- | --- | --- | --- | --- | --- | --- | --- | --- |
| 10-14 | 0.23 (0.16-0.32) | 0.08 (0.07-0.09) | 0.00 (0.00-0.00) | 0.00 (0.00-0.00) | 0.04 (0.03-0.05) | 0.00 (0.00-0.00) | | 0.00 (0.00-0.00) | 0.00 (0.00-0.00) | 0.00 (0.00-0.00) | 0.00 (0.00-0.00) | 0.00 (0.00-0.00) |
| 15-19 | 0.23 (0.16-0.31) | 0.09 (0.08-0.10) | 0.00 (0.00-0.00) | 0.00 (0.00-0.00) | 0.05 (0.04-0.06) | 0.00 (0.00-0.00) | 0.00 (0.00-0.00) | 0.01 (0.01-0.01) | 0.00 (0.00-0.00) | 0.00 (0.00-0.00) | 0.00 (0.00-0.01) | |
| 20-24 | 0.22 (0.15-0.31) | 0.11 (0.10-0.13) | 0.00 (0.00-0.00) | 0.00 (0.00-0.00) | 0.04 (0.03-0.05) | 0.00 (0.00-0.00) | 0.00 (0.00-0.00) | 0.01 (0.01-0.02) | 0.00 (0.00-0.00) | 0.00 (0.00-0.00) | 0.01 (0.00-0.01) | |
| 25-29 | 0.22 (0.15-0.30) | 0.12 (0.10-0.13) | 0.00 (0.00-0.00) | 0.00 (0.00-0.00) | 0.04 (0.03-0.05) | 0.00 (0.00-0.00) | 0.00 (0.00-0.00) | 0.02 (0.02-0.03) | 0.00 (0.00-0.00) | 0.00 (0.00-0.00) | 0.01 (0.01-0.01) | |
| 30-34 | 0.22 (0.15-0.30) | 0.11 (0.09-0.12) | 0.01 (0.00-0.01) | 0.00 (0.00-0.00) | 0.04 (0.03-0.06) | 0.00 (0.00-0.00) | 0.00 (0.00-0.00) | 0.03 (0.02-0.04) | 0.00 (0.00-0.00) | 0.00 (0.00-0.00) | 0.01 (0.01-0.01) | |
| 35-39 | 0.22 (0.15-0.30) | 0.10 (0.09-0.12) | 0.01 (0.01-0.01) | 0.00 (0.00-0.01) | 0.05 (0.04-0.06) | 0.01 (0.00-0.01) | 0.00 (0.00-0.00) | 0.04 (0.03-0.05) | 0.00 (0.00-0.00) | 0.00 (0.00-0.00) | 0.01 (0.01-0.01) | |
| 40-44 | 0.21 (0.15-0.29) | 0.10 (0.09-0.11) | 0.01 (0.01-0.02) | 0.01 (0.00-0.01) | 0.05 (0.04-0.06) | 0.01 (0.01-0.01) | 0.00 (0.00-0.00) | 0.05 (0.03-0.06) | 0.00 (0.00-0.00) | 0.00 (0.00-0.00) | 0.01 (0.01-0.01) | |
| 45-49 | 0.21 (0.14-0.29) | 0.10 (0.09-0.11) | 0.02 (0.02-0.03) | 0.01 (0.01-0.01) | 0.06 (0.05-0.07) | 0.01 (0.01-0.01) | 0.00 (0.00-0.00) | 0.05 (0.04-0.06) | 0.00 (0.00-0.00) | 0.00 (0.00-0.00) | 0.01 (0.01-0.01) | |
| 50-54 | 0.21 (0.14-0.28) | 0.10 (0.08-0.11) | 0.03 (0.02-0.03) | 0.01 (0.01-0.01) | 0.06 (0.05-0.07) | 0.01 (0.01-0.01) | 0.00 (0.00-0.00) | 0.06 (0.04-0.07) | 0.00 (0.00-0.00) | 0.00 (0.00-0.00) | 0.01 (0.01-0.02) | |
| 55-59 | 0.21 (0.14-0.28) | 0.09 (0.08-0.10) | 0.04 (0.03-0.04) | 0.01 (0.01-0.01) | 0.06 (0.05-0.07) | 0.01 (0.01-0.01) | 0.00 (0.00-0.00) | 0.06 (0.05-0.08) | 0.00 (0.00-0.00) | 0.00 (0.00-0.00) | 0.01 (0.01-0.02) | |
| 60-64 | 0.20 (0.14-0.28) | 0.08 (0.07-0.09) | 0.05 (0.04-0.06) | 0.01 (0.01-0.01) | 0.06 (0.04-0.07) | 0.01 (0.01-0.01) | 0.00 (0.00-0.00) | 0.06 (0.05-0.08) | 0.00 (0.00-0.00) | 0.01 (0.01-0.01) | 0.02 (0.01-0.02) | |
| 65-69 | 0.20 (0.14-0.27) | 0.06 (0.06-0.07) | 0.06 (0.05-0.07) | 0.01 (0.01-0.02) | 0.06 (0.05-0.07) | 0.01 (0.01-0.01) | 0.00 (0.00-0.01) | 0.07 (0.06-0.09) | 0.00 (0.00-0.00) | 0.01 (0.01-0.01) | 0.02 (0.02-0.02) | |
| 70-74 | 0.20 (0.14-0.27) | 0.05 (0.04-0.05) | 0.07 (0.06-0.08) | 0.01 (0.01-0.02) | 0.06 (0.04-0.07) | 0.01 (0.01-0.01) | 0.01 (0.00-0.01) | 0.08 (0.06-0.10) | 0.00 (0.00-0.00) | 0.01 (0.01-0.01) | 0.02 (0.02-0.03) | |
| 75+ | 0.20 (0.14-0.27) | 0.03 (0.02-0.03) | 0.09 (0.07-0.10) | 0.01 (0.01-0.02) | 0.05 (0.04-0.06) | 0.01 (0.01-0.01) | 0.01 (0.00-0.01) | 0.10 (0.08-0.12) | 0.00 (0.00-0.00) | 0.01 (0.01-0.01) | 0.03 (0.03-0.04) | |

**Supplementary table 15.** Age-specific health loss proportion (HeLP) and 95% confidence interval for persons diagnosed with anxiety disorders during 2000-2015. All estimates are adjusted for observed comorbidity from mental and substance use disorders and general medical conditions.

|  | Index disorder | Mental disorders | Circulatory diseases | Endocrine diseases | Pulmonary diseases | Gastrointestinal diseases | Urogenital diseases | Musculoskeletal diseases | Hematological diseases | Cancers | Neurological diseases | Other |
| --- | --- | --- | --- | --- | --- | --- | --- | --- | --- | --- | --- | --- |
| 0-4 | 0.14 (0.10-0.19) | 0.03 (0.02-0.04) | |  | 0.03 (0.02-0.04) | |  |  |  |  | 0.01 (0.00-0.02) | 0.00 (0.00-0.01) |
| 5-9 | 0.14 (0.10-0.19) | 0.03 (0.03-0.04) | 0.00 (0.00-0.00) | 0.00 (0.00-0.00) | 0.04 (0.03-0.05) | |  | 0.00 (0.00-0.00) | 0.00 (0.00-0.00) | 0.00 (0.00-0.00) | 0.00 (0.00-0.01) | 0.00 (0.00-0.00) |
| 10-14 | 0.14 (0.10-0.18) | 0.04 (0.04-0.05) | 0.00 (0.00-0.00) | 0.00 (0.00-0.00) | 0.05 (0.04-0.05) | 0.00 (0.00-0.00) | 0.00 (0.00-0.00) | 0.00 (0.00-0.00) | 0.00 (0.00-0.00) | 0.00 (0.00-0.00) | 0.00 (0.00-0.01) | |
| 15-19 | 0.14 (0.10-0.18) | 0.07 (0.06-0.08) | 0.00 (0.00-0.00) | 0.00 (0.00-0.00) | 0.05 (0.04-0.06) | 0.00 (0.00-0.00) | 0.00 (0.00-0.00) | 0.01 (0.00-0.01) | 0.00 (0.00-0.00) | 0.00 (0.00-0.00) | 0.01 (0.00-0.01) | |
| 20-24 | 0.13 (0.10-0.18) | 0.10 (0.09-0.11) | 0.00 (0.00-0.00) | 0.00 (0.00-0.00) | 0.05 (0.04-0.06) | 0.00 (0.00-0.00) | 0.00 (0.00-0.00) | 0.01 (0.01-0.02) | 0.00 (0.00-0.00) | 0.00 (0.00-0.00) | 0.01 (0.01-0.01) | |
| 25-29 | 0.13 (0.10-0.18) | 0.11 (0.09-0.12) | 0.00 (0.00-0.00) | 0.00 (0.00-0.00) | 0.05 (0.04-0.06) | 0.00 (0.00-0.00) | 0.00 (0.00-0.00) | 0.02 (0.02-0.03) | 0.00 (0.00-0.00) | 0.00 (0.00-0.00) | 0.01 (0.01-0.01) | |
| 30-34 | 0.13 (0.09-0.17) | 0.10 (0.09-0.11) | 0.01 (0.01-0.01) | 0.00 (0.00-0.00) | 0.05 (0.04-0.06) | 0.00 (0.00-0.01) | 0.00 (0.00-0.00) | 0.03 (0.02-0.04) | 0.00 (0.00-0.00) | 0.00 (0.00-0.00) | 0.01 (0.01-0.01) | |
| 35-39 | 0.13 (0.09-0.17) | 0.10 (0.09-0.11) | 0.01 (0.01-0.01) | 0.00 (0.00-0.01) | 0.06 (0.04-0.07) | 0.01 (0.00-0.01) | 0.00 (0.00-0.00) | 0.04 (0.03-0.05) | 0.00 (0.00-0.00) | 0.00 (0.00-0.00) | 0.01 (0.01-0.01) | |
| 40-44 | 0.13 (0.09-0.17) | 0.09 (0.08-0.10) | 0.02 (0.01-0.02) | 0.01 (0.00-0.01) | 0.06 (0.05-0.07) | 0.01 (0.01-0.01) | 0.00 (0.00-0.00) | 0.05 (0.04-0.06) | 0.00 (0.00-0.00) | 0.00 (0.00-0.00) | 0.01 (0.01-0.01) | |
| 45-49 | 0.12 (0.09-0.17) | 0.09 (0.08-0.10) | 0.02 (0.02-0.03) | 0.01 (0.01-0.01) | 0.07 (0.05-0.08) | 0.01 (0.01-0.01) | 0.00 (0.00-0.00) | 0.06 (0.04-0.07) | 0.00 (0.00-0.00) | 0.00 (0.00-0.00) | 0.01 (0.01-0.02) | |
| 50-54 | 0.12 (0.09-0.16) | 0.09 (0.08-0.10) | 0.03 (0.03-0.04) | 0.01 (0.01-0.01) | 0.07 (0.06-0.08) | 0.01 (0.01-0.01) | 0.00 (0.00-0.00) | 0.06 (0.05-0.08) | 0.00 (0.00-0.00) | 0.00 (0.00-0.00) | 0.01 (0.01-0.02) | |
| 55-59 | 0.12 (0.09-0.16) | 0.09 (0.08-0.10) | 0.04 (0.04-0.05) | 0.01 (0.01-0.01) | 0.07 (0.06-0.09) | 0.01 (0.01-0.01) | 0.00 (0.00-0.00) | 0.07 (0.05-0.09) | 0.00 (0.00-0.00) | 0.00 (0.00-0.00) | 0.02 (0.01-0.02) | |
| 60-64 | 0.12 (0.09-0.16) | 0.08 (0.07-0.09) | 0.05 (0.05-0.06) | 0.01 (0.01-0.02) | 0.07 (0.06-0.09) | 0.01 (0.01-0.01) | 0.00 (0.00-0.00) | 0.07 (0.06-0.09) | 0.00 (0.00-0.00) | 0.01 (0.00-0.01) | 0.02 (0.02-0.02) | |
| 65-69 | 0.12 (0.08-0.15) | 0.07 (0.06-0.08) | 0.07 (0.06-0.08) | 0.01 (0.01-0.02) | 0.08 (0.06-0.09) | 0.01 (0.01-0.01) | 0.00 (0.00-0.00) | 0.08 (0.06-0.10) | 0.00 (0.00-0.00) | 0.01 (0.01-0.01) | 0.02 (0.02-0.03) | |
| 70-74 | 0.11 (0.08-0.15) | 0.06 (0.05-0.07) | 0.08 (0.06-0.09) | 0.01 (0.01-0.02) | 0.07 (0.06-0.09) | 0.01 (0.01-0.01) | 0.00 (0.00-0.00) | 0.09 (0.07-0.11) | 0.00 (0.00-0.00) | 0.01 (0.01-0.01) | 0.03 (0.02-0.03) | |
| 75+ | 0.11 (0.08-0.15) | 0.05 (0.04-0.05) | 0.09 (0.08-0.11) | 0.01 (0.01-0.02) | 0.07 (0.06-0.08) | 0.01 (0.01-0.01) | 0.00 (0.00-0.01) | 0.11 (0.08-0.13) | 0.00 (0.00-0.00) | 0.01 (0.01-0.01) | 0.04 (0.03-0.04) | |

**Supplementary table 16.** Age-specific health loss proportion (HeLP) and 95% confidence interval for persons diagnosed with anorexia during 2000-2015. All estimates are adjusted for observed comorbidity from mental and substance use disorders and general medical conditions.

|  | Index disorder | Mental disorders | Circulatory diseases | Endocrine diseases | Pulmonary diseases | Gastrointestinal diseases | Urogenital diseases | Musculoskeletal diseases | Hematological diseases | Cancers | Neurological diseases | Other |
| --- | --- | --- | --- | --- | --- | --- | --- | --- | --- | --- | --- | --- |
| 0-4 |  |  |  |  |  |  |  |  |  |  |  |  |
| 5-9 | 0.22 (0.16-0.28) | |  |  |  |  |  |  |  |  |  | 0.03 (0.01-0.05) |
| 10-14 | 0.21 (0.16-0.27) | 0.04 (0.04-0.05) | 0.00 (0.00-0.00) | 0.00 (0.00-0.00) | 0.04 (0.03-0.05) | 0.00 (0.00-0.00) | 0.00 (0.00-0.00) | 0.00 (0.00-0.00) | 0.00 (0.00-0.00) | 0.00 (0.00-0.00) | 0.00 (0.00-0.00) | |
| 15-19 | 0.21 (0.15-0.27) | 0.06 (0.05-0.07) | 0.00 (0.00-0.00) | 0.00 (0.00-0.00) | 0.04 (0.03-0.05) | 0.00 (0.00-0.00) | 0.00 (0.00-0.00) | 0.00 (0.00-0.01) | 0.00 (0.00-0.00) | 0.00 (0.00-0.00) | 0.00 (0.00-0.00) | |
| 20-24 | 0.20 (0.15-0.26) | 0.09 (0.08-0.10) | 0.00 (0.00-0.00) | 0.00 (0.00-0.00) | 0.04 (0.03-0.05) | 0.00 (0.00-0.00) | 0.00 (0.00-0.00) | 0.01 (0.01-0.01) | 0.00 (0.00-0.00) | 0.00 (0.00-0.00) | 0.00 (0.00-0.01) | |
| 25-29 | 0.20 (0.15-0.26) | 0.10 (0.09-0.12) | 0.00 (0.00-0.00) | 0.00 (0.00-0.00) | 0.04 (0.03-0.05) | 0.00 (0.00-0.00) | 0.00 (0.00-0.00) | 0.02 (0.02-0.03) | 0.00 (0.00-0.00) | 0.00 (0.00-0.00) | 0.01 (0.00-0.01) | |
| 30-34 | 0.19 (0.14-0.25) | 0.12 (0.10-0.14) | 0.01 (0.00-0.01) | 0.00 (0.00-0.01) | 0.04 (0.03-0.06) | 0.00 (0.00-0.01) | 0.00 (0.00-0.00) | 0.03 (0.03-0.04) | 0.00 (0.00-0.00) | 0.00 (0.00-0.00) | 0.01 (0.01-0.01) | |
| 35-39 | 0.19 (0.14-0.24) | 0.12 (0.11-0.14) | 0.01 (0.01-0.01) | 0.00 (0.00-0.01) | 0.05 (0.04-0.07) | 0.01 (0.00-0.01) | 0.00 (0.00-0.00) | 0.04 (0.03-0.05) | 0.00 (0.00-0.00) | 0.00 (0.00-0.00) | 0.01 (0.01-0.01) | |
| 40-44 | 0.19 (0.14-0.24) | 0.12 (0.11-0.15) | 0.01 (0.01-0.02) | 0.00 (0.00-0.01) | 0.05 (0.04-0.07) | 0.01 (0.00-0.01) | 0.00 (0.00-0.00) | 0.06 (0.05-0.07) | 0.00 (0.00-0.00) | 0.00 (0.00-0.00) | 0.01 (0.01-0.02) | |
| 45-49 | 0.18 (0.14-0.23) | 0.12 (0.10-0.15) | 0.02 (0.01-0.02) | 0.01 (0.00-0.01) | 0.06 (0.04-0.08) | 0.01 (0.01-0.02) | 0.00 (0.00-0.00) | 0.07 (0.05-0.08) | 0.00 (0.00-0.00) | 0.00 (0.00-0.00) | 0.02 (0.01-0.02) | |
| 50-54 | 0.17 (0.13-0.23) | 0.13 (0.11-0.17) | 0.03 (0.02-0.03) | 0.01 (0.00-0.01) | 0.06 (0.05-0.08) | 0.01 (0.01-0.02) | 0.00 (0.00-0.00) | 0.08 (0.06-0.10) | 0.00 (0.00-0.00) | 0.00 (0.00-0.00) | 0.02 (0.01-0.03) | |
| 55-59 | 0.17 (0.13-0.22) | 0.12 (0.10-0.16) | 0.03 (0.02-0.04) | 0.01 (0.00-0.01) | 0.07 (0.05-0.09) | 0.02 (0.01-0.03) | 0.00 (0.00-0.00) | 0.09 (0.07-0.11) | 0.00 (0.00-0.00) | 0.00 (0.00-0.01) | 0.02 (0.01-0.03) | |
| 60-64 | 0.17 (0.13-0.22) | 0.12 (0.08-0.17) | 0.03 (0.03-0.05) | 0.01 (0.00-0.01) | 0.07 (0.04-0.09) | 0.01 (0.01-0.02) | 0.00 (0.00-0.00) | 0.11 (0.08-0.13) | 0.00 (0.00-0.00) | 0.01 (0.00-0.01) | 0.02 (0.01-0.04) | |
| 65-69 | 0.16 (0.12-0.21) | 0.13 (0.07-0.18) | 0.04 (0.02-0.06) | 0.01 (0.00-0.02) | 0.08 (0.05-0.12) | 0.01 (0.00-0.01) | 0.00 (0.00-0.00) | 0.13 (0.09-0.15) | 0.00 (0.00-0.01) | 0.00 (0.00-0.01) | 0.03 (0.02-0.05) | |
| 70-74 | 0.16 (0.12-0.22) | 0.19 (0.05-0.28) | 0.09 (0.02-0.10) | 0.01 (0.00-0.03) | 0.09 (0.03-0.14) | |  | 0.08 (0.04-0.14) | 0.00 (0.00-0.01) | | 0.02 (0.01-0.05) | 0.01 (0.00-0.03) |
| 75+ | 0.16 (0.12-0.21) | 0.12 (0.03-0.30) | 0.07 (0.03-0.10) | 0.01 (0.00-0.03) | 0.04 (0.00-0.10) | |  | 0.12 (0.06-0.16) | |  | 0.08 (0.01-0.14) | 0.02 (0.01-0.04) |

**Supplementary table 17.** Age-specific health loss proportion (HeLP) and 95% confidence interval for persons diagnosed with bulimia during 2000-2015. All estimates are adjusted for observed comorbidity from mental and substance use disorders and general medical conditions.

|  | Index disorder | Mental disorders | Circulatory diseases | Endocrine diseases | Pulmonary diseases | Gastrointestinal diseases | Urogenital diseases | Musculoskeletal diseases | Hematological diseases | Cancers | Neurological diseases | Other |
| --- | --- | --- | --- | --- | --- | --- | --- | --- | --- | --- | --- | --- |
| 10-14 | 0.20 (0.15-0.26) | 0.10 (0.06-0.12) | |  | 0.04 (0.02-0.06) | |  |  |  |  | 0.00 (0.00-0.01) | 0.01 (0.00-0.01) |
| 15-19 | 0.20 (0.15-0.26) | 0.09 (0.07-0.10) | 0.00 (0.00-0.00) | 0.00 (0.00-0.00) | 0.04 (0.03-0.05) | |  | 0.01 (0.00-0.01) | 0.00 (0.00-0.00) | | 0.00 (0.00-0.00) | 0.00 (0.00-0.00) |
| 20-24 | 0.20 (0.15-0.26) | 0.10 (0.09-0.11) | 0.00 (0.00-0.00) | 0.00 (0.00-0.00) | 0.04 (0.03-0.05) | 0.00 (0.00-0.00) | 0.00 (0.00-0.00) | 0.01 (0.01-0.01) | 0.00 (0.00-0.00) | 0.00 (0.00-0.00) | 0.00 (0.00-0.00) | |
| 25-29 | 0.20 (0.15-0.26) | 0.11 (0.09-0.12) | 0.00 (0.00-0.00) | 0.00 (0.00-0.00) | 0.04 (0.03-0.05) | 0.00 (0.00-0.00) | 0.00 (0.00-0.00) | 0.01 (0.01-0.02) | 0.00 (0.00-0.00) | 0.00 (0.00-0.00) | 0.01 (0.00-0.01) | |
| 30-34 | 0.19 (0.15-0.25) | 0.12 (0.10-0.13) | 0.00 (0.00-0.01) | 0.00 (0.00-0.01) | 0.05 (0.03-0.06) | 0.00 (0.00-0.01) | 0.00 (0.00-0.00) | 0.02 (0.02-0.03) | 0.00 (0.00-0.00) | 0.00 (0.00-0.00) | 0.01 (0.01-0.01) | |
| 35-39 | 0.19 (0.14-0.25) | 0.12 (0.10-0.14) | 0.01 (0.00-0.01) | 0.00 (0.00-0.01) | 0.05 (0.04-0.07) | 0.01 (0.00-0.01) | 0.00 (0.00-0.00) | 0.03 (0.02-0.04) | 0.00 (0.00-0.00) | 0.00 (0.00-0.00) | 0.01 (0.01-0.01) | |
| 40-44 | 0.19 (0.14-0.24) | 0.12 (0.10-0.14) | 0.01 (0.01-0.01) | 0.01 (0.00-0.01) | 0.06 (0.04-0.07) | 0.01 (0.00-0.01) | 0.00 (0.00-0.00) | 0.04 (0.03-0.05) | 0.00 (0.00-0.00) | 0.00 (0.00-0.00) | 0.01 (0.01-0.01) | |
| 45-49 | 0.18 (0.14-0.23) | 0.13 (0.11-0.16) | 0.01 (0.01-0.02) | 0.00 (0.00-0.01) | 0.06 (0.04-0.08) | 0.01 (0.00-0.01) | | 0.05 (0.04-0.07) | | 0.00 (0.00-0.01) | 0.01 (0.01-0.02) | 0.00 (0.00-0.00) |
| 50-54 | 0.18 (0.13-0.22) | 0.14 (0.11-0.18) | 0.03 (0.02-0.04) | 0.01 (0.00-0.01) | 0.06 (0.04-0.08) | 0.01 (0.00-0.02) | | 0.07 (0.05-0.09) | | 0.00 (0.00-0.01) | 0.02 (0.01-0.02) | 0.00 (0.00-0.00) |
| 55-59 | 0.17 (0.13-0.22) | 0.15 (0.11-0.19) | 0.04 (0.03-0.05) | 0.02 (0.01-0.02) | 0.05 (0.04-0.09) | 0.00 (0.00-0.01) | | 0.07 (0.05-0.10) | | 0.00 (0.00-0.01) | 0.02 (0.01-0.03) | 0.00 (0.00-0.00) |
| 60-64 | 0.18 (0.13-0.22) | 0.10 (0.07-0.16) | 0.05 (0.03-0.08) | 0.03 (0.01-0.04) | 0.05 (0.03-0.09) | |  | 0.07 (0.05-0.11) | |  | 0.01 (0.01-0.04) | 0.01 (0.00-0.01) |
| 65-69 | 0.17 (0.12-0.22) | 0.12 (0.05-0.20) | 0.07 (0.04-0.12) | 0.02 (0.01-0.05) | 0.04 (0.01-0.09) | |  | 0.06 (0.03-0.11) | |  | 0.02 (0.01-0.06) | 0.01 (0.00-0.02) |
| 70-74 | 0.17 (0.12-0.22) | |  |  |  |  |  |  |  |  |  | 0.34 (0.29-0.58) |
| 75+ |  |  |  |  |  |  |  |  |  |  |  |  |

**Supplementary table 18.** Age-specific health loss proportion (HeLP) and 95% confidence interval for persons diagnosed with personality disorders during 2000-2015. All estimates are adjusted for observed comorbidity from mental and substance use disorders and general medical conditions.

|  | Index disorder | Mental disorders | Circulatory diseases | Endocrine diseases | Pulmonary diseases | Gastrointestinal diseases | Urogenital diseases | Musculoskeletal diseases | Hematological diseases | Cancers | Neurological diseases | Other |
| --- | --- | --- | --- | --- | --- | --- | --- | --- | --- | --- | --- | --- |
| 10-14 | 0.14 (0.10-0.18) | 0.09 (0.07-0.10) | 0.00 (0.00-0.00) | 0.00 (0.00-0.00) | 0.04 (0.03-0.05) | |  | 0.00 (0.00-0.00) | |  | 0.00 (0.00-0.01) | 0.00 (0.00-0.00) |
| 15-19 | 0.14 (0.10-0.18) | 0.09 (0.08-0.10) | 0.00 (0.00-0.00) | 0.00 (0.00-0.00) | 0.05 (0.04-0.06) | 0.00 (0.00-0.00) | 0.00 (0.00-0.00) | 0.01 (0.01-0.01) | 0.00 (0.00-0.00) | 0.00 (0.00-0.00) | 0.00 (0.00-0.01) | |
| 20-24 | 0.14 (0.10-0.18) | 0.11 (0.10-0.12) | 0.00 (0.00-0.00) | 0.00 (0.00-0.00) | 0.05 (0.04-0.06) | 0.00 (0.00-0.00) | 0.00 (0.00-0.00) | 0.02 (0.01-0.02) | 0.00 (0.00-0.00) | 0.00 (0.00-0.00) | 0.01 (0.01-0.01) | |
| 25-29 | 0.14 (0.10-0.18) | 0.12 (0.10-0.13) | 0.00 (0.00-0.00) | 0.00 (0.00-0.00) | 0.05 (0.04-0.06) | 0.00 (0.00-0.00) | 0.00 (0.00-0.00) | 0.03 (0.02-0.03) | 0.00 (0.00-0.00) | 0.00 (0.00-0.00) | 0.01 (0.01-0.01) | |
| 30-34 | 0.13 (0.10-0.17) | 0.12 (0.11-0.13) | 0.01 (0.00-0.01) | 0.00 (0.00-0.00) | 0.05 (0.04-0.06) | 0.01 (0.00-0.01) | 0.00 (0.00-0.00) | 0.04 (0.03-0.04) | 0.00 (0.00-0.00) | 0.00 (0.00-0.00) | 0.01 (0.01-0.01) | |
| 35-39 | 0.13 (0.10-0.17) | 0.12 (0.11-0.13) | 0.01 (0.01-0.01) | 0.00 (0.00-0.01) | 0.05 (0.04-0.07) | 0.01 (0.01-0.01) | 0.00 (0.00-0.00) | 0.04 (0.03-0.05) | 0.00 (0.00-0.00) | 0.00 (0.00-0.00) | 0.01 (0.01-0.01) | |
| 40-44 | 0.13 (0.09-0.17) | 0.12 (0.11-0.13) | 0.01 (0.01-0.02) | 0.01 (0.00-0.01) | 0.06 (0.05-0.07) | 0.01 (0.01-0.01) | 0.00 (0.00-0.00) | 0.05 (0.04-0.06) | 0.00 (0.00-0.00) | 0.00 (0.00-0.00) | 0.01 (0.01-0.02) | |
| 45-49 | 0.13 (0.09-0.17) | 0.11 (0.10-0.13) | 0.02 (0.02-0.02) | 0.01 (0.01-0.01) | 0.07 (0.05-0.08) | 0.01 (0.01-0.01) | 0.00 (0.00-0.00) | 0.06 (0.04-0.07) | 0.00 (0.00-0.00) | 0.00 (0.00-0.00) | 0.01 (0.01-0.02) | |
| 50-54 | 0.13 (0.09-0.16) | 0.11 (0.10-0.12) | 0.03 (0.02-0.03) | 0.01 (0.01-0.01) | 0.07 (0.06-0.08) | 0.01 (0.01-0.01) | 0.00 (0.00-0.00) | 0.07 (0.05-0.08) | 0.00 (0.00-0.00) | 0.00 (0.00-0.00) | 0.02 (0.01-0.02) | |
| 55-59 | 0.12 (0.09-0.16) | 0.10 (0.09-0.11) | 0.04 (0.03-0.05) | 0.01 (0.01-0.01) | 0.07 (0.06-0.09) | 0.01 (0.01-0.01) | 0.00 (0.00-0.00) | 0.07 (0.06-0.09) | 0.00 (0.00-0.00) | 0.00 (0.00-0.00) | 0.02 (0.01-0.02) | |
| 60-64 | 0.12 (0.09-0.16) | 0.09 (0.08-0.10) | 0.05 (0.04-0.06) | 0.01 (0.01-0.02) | 0.07 (0.06-0.09) | 0.01 (0.01-0.01) | 0.00 (0.00-0.00) | 0.08 (0.06-0.10) | 0.00 (0.00-0.00) | 0.01 (0.00-0.01) | 0.02 (0.02-0.02) | |
| 65-69 | 0.12 (0.09-0.15) | 0.08 (0.07-0.09) | 0.06 (0.05-0.07) | 0.01 (0.01-0.02) | 0.07 (0.06-0.09) | 0.01 (0.01-0.01) | 0.00 (0.00-0.01) | 0.09 (0.07-0.11) | 0.00 (0.00-0.00) | 0.01 (0.01-0.01) | 0.02 (0.02-0.03) | |
| 70-74 | 0.12 (0.09-0.15) | 0.07 (0.06-0.08) | 0.07 (0.06-0.08) | 0.02 (0.01-0.02) | 0.08 (0.06-0.09) | 0.01 (0.01-0.01) | 0.00 (0.00-0.01) | 0.09 (0.07-0.11) | 0.00 (0.00-0.00) | 0.01 (0.01-0.01) | 0.03 (0.02-0.03) | |
| 75+ | 0.12 (0.08-0.15) | 0.06 (0.05-0.07) | 0.08 (0.07-0.10) | 0.01 (0.01-0.02) | 0.07 (0.05-0.08) | 0.01 (0.01-0.01) | 0.00 (0.00-0.01) | 0.11 (0.08-0.13) | 0.00 (0.00-0.00) | 0.01 (0.01-0.01) | 0.03 (0.03-0.04) | |

**Supplementary table 19.** Age-specific health loss proportion (HeLP) and 95% confidence interval for persons diagnosed with intellectual disabilities during 2000-2015. All estimates are adjusted for observed comorbidity from mental and substance use disorders and general medical conditions.

|  | Index disorder | Mental disorders | Circulatory diseases | Endocrine diseases | Pulmonary diseases | Gastrointestinal diseases | Urogenital diseases | Musculoskeletal diseases | Hematological diseases | Cancers | Neurological diseases | Other |
| --- | --- | --- | --- | --- | --- | --- | --- | --- | --- | --- | --- | --- |
| 0-4 | 0.06 (0.03-0.12) | 0.11 (0.08-0.14) | 0.00 (0.00-0.00) | 0.00 (0.00-0.00) | 0.02 (0.02-0.03) | |  | 0.00 (0.00-0.00) | 0.00 (0.00-0.00) | | 0.02 (0.01-0.03) | 0.00 (0.00-0.00) |
| 5-9 | 0.06 (0.03-0.12) | 0.09 (0.07-0.11) | 0.00 (0.00-0.00) | 0.00 (0.00-0.00) | 0.03 (0.03-0.04) | 0.00 (0.00-0.00) | 0.00 (0.00-0.00) | 0.00 (0.00-0.00) | 0.00 (0.00-0.00) | 0.00 (0.00-0.00) | 0.02 (0.02-0.03) | |
| 10-14 | 0.06 (0.03-0.12) | 0.08 (0.06-0.09) | 0.00 (0.00-0.00) | 0.00 (0.00-0.00) | 0.04 (0.04-0.05) | 0.00 (0.00-0.00) | 0.00 (0.00-0.00) | 0.00 (0.00-0.00) | 0.00 (0.00-0.00) | 0.00 (0.00-0.00) | 0.03 (0.02-0.03) | |
| 15-19 | 0.06 (0.03-0.12) | 0.09 (0.07-0.10) | 0.00 (0.00-0.00) | 0.00 (0.00-0.00) | 0.05 (0.04-0.06) | 0.00 (0.00-0.00) | 0.00 (0.00-0.00) | 0.01 (0.00-0.01) | 0.00 (0.00-0.00) | 0.00 (0.00-0.00) | 0.03 (0.02-0.03) | |
| 20-24 | 0.06 (0.02-0.12) | 0.11 (0.10-0.13) | 0.00 (0.00-0.00) | 0.00 (0.00-0.00) | 0.05 (0.04-0.06) | 0.00 (0.00-0.00) | 0.00 (0.00-0.00) | 0.01 (0.01-0.02) | 0.00 (0.00-0.00) | 0.00 (0.00-0.00) | 0.03 (0.02-0.04) | |
| 25-29 | 0.06 (0.02-0.12) | 0.13 (0.12-0.14) | 0.00 (0.00-0.00) | 0.00 (0.00-0.00) | 0.05 (0.04-0.06) | 0.00 (0.00-0.00) | 0.00 (0.00-0.00) | 0.02 (0.02-0.03) | 0.00 (0.00-0.00) | 0.00 (0.00-0.00) | 0.04 (0.03-0.05) | |
| 30-34 | 0.06 (0.02-0.11) | 0.14 (0.12-0.16) | 0.01 (0.00-0.01) | 0.00 (0.00-0.00) | 0.05 (0.04-0.06) | 0.00 (0.00-0.00) | 0.00 (0.00-0.00) | 0.03 (0.02-0.04) | 0.00 (0.00-0.00) | 0.00 (0.00-0.00) | 0.04 (0.03-0.05) | |
| 35-39 | 0.06 (0.02-0.11) | 0.14 (0.12-0.16) | 0.01 (0.01-0.01) | 0.01 (0.00-0.01) | 0.05 (0.04-0.07) | 0.00 (0.00-0.00) | 0.00 (0.00-0.00) | 0.03 (0.02-0.04) | 0.00 (0.00-0.00) | 0.00 (0.00-0.00) | 0.04 (0.03-0.05) | |
| 40-44 | 0.06 (0.02-0.11) | 0.13 (0.12-0.15) | 0.01 (0.01-0.02) | 0.01 (0.01-0.01) | 0.05 (0.04-0.06) | 0.00 (0.00-0.01) | 0.00 (0.00-0.00) | 0.04 (0.03-0.05) | 0.00 (0.00-0.00) | 0.00 (0.00-0.00) | 0.04 (0.03-0.05) | |
| 45-49 | 0.06 (0.02-0.11) | 0.13 (0.12-0.15) | 0.02 (0.02-0.02) | 0.01 (0.01-0.01) | 0.05 (0.04-0.07) | 0.00 (0.00-0.01) | 0.00 (0.00-0.00) | 0.05 (0.03-0.06) | 0.00 (0.00-0.00) | 0.00 (0.00-0.00) | 0.04 (0.03-0.05) | |
| 50-54 | 0.06 (0.02-0.11) | 0.13 (0.11-0.14) | 0.02 (0.02-0.03) | 0.01 (0.01-0.01) | 0.05 (0.04-0.06) | 0.01 (0.00-0.01) | 0.00 (0.00-0.00) | 0.05 (0.04-0.06) | 0.00 (0.00-0.00) | 0.00 (0.00-0.00) | 0.04 (0.03-0.05) | |
| 55-59 | 0.06 (0.02-0.11) | 0.12 (0.10-0.13) | 0.03 (0.02-0.04) | 0.01 (0.01-0.02) | 0.05 (0.04-0.06) | 0.01 (0.00-0.01) | 0.00 (0.00-0.00) | 0.06 (0.04-0.07) | 0.00 (0.00-0.00) | 0.00 (0.00-0.00) | 0.04 (0.03-0.05) | |
| 60-64 | 0.06 (0.02-0.11) | 0.12 (0.10-0.13) | 0.04 (0.03-0.04) | 0.01 (0.01-0.02) | 0.05 (0.04-0.06) | 0.01 (0.01-0.01) | 0.00 (0.00-0.01) | 0.06 (0.05-0.08) | 0.00 (0.00-0.00) | 0.00 (0.00-0.00) | 0.04 (0.04-0.06) | |
| 65-69 | 0.06 (0.02-0.11) | 0.11 (0.10-0.13) | 0.05 (0.04-0.06) | 0.01 (0.01-0.02) | 0.05 (0.04-0.06) | 0.01 (0.01-0.01) | 0.01 (0.00-0.01) | 0.07 (0.06-0.09) | 0.00 (0.00-0.00) | 0.00 (0.00-0.01) | 0.04 (0.04-0.05) | |
| 70-74 | 0.06 (0.02-0.11) | 0.10 (0.08-0.12) | 0.06 (0.05-0.07) | 0.01 (0.01-0.02) | 0.05 (0.04-0.06) | 0.01 (0.01-0.01) | 0.01 (0.00-0.01) | 0.09 (0.06-0.11) | 0.00 (0.00-0.00) | 0.00 (0.00-0.01) | 0.04 (0.03-0.05) | |
| 75+ | 0.06 (0.02-0.11) | 0.08 (0.06-0.09) | 0.06 (0.05-0.08) | 0.01 (0.01-0.02) | 0.04 (0.03-0.06) | 0.01 (0.00-0.01) | 0.01 (0.00-0.01) | 0.10 (0.07-0.12) | 0.00 (0.00-0.00) | 0.01 (0.00-0.01) | 0.04 (0.03-0.05) | |

**Supplementary table 20.** Age-specific health loss proportion (HeLP) and 95% confidence interval for persons diagnosed with autism spectrum disorders during 2000-2015. All estimates are adjusted for observed comorbidity from mental and substance use disorders and general medical conditions.

|  | Index disorder | Mental disorders | Circulatory diseases | Endocrine diseases | Pulmonary diseases | Gastrointestinal diseases | Urogenital diseases | Musculoskeletal diseases | Hematological diseases | Cancers | Neurological diseases | Other |
| --- | --- | --- | --- | --- | --- | --- | --- | --- | --- | --- | --- | --- |
| 0-4 | 0.17 (0.12-0.20) | 0.02 (0.01-0.04) | 0.00 (0.00-0.00) | 0.00 (0.00-0.00) | 0.02 (0.02-0.03) | 0.00 (0.00-0.00) | | 0.00 (0.00-0.00) | 0.00 (0.00-0.00) | | 0.01 (0.01-0.01) | 0.00 (0.00-0.00) |
| 5-9 | 0.16 (0.12-0.20) | 0.02 (0.02-0.04) | 0.00 (0.00-0.00) | 0.00 (0.00-0.00) | 0.03 (0.03-0.04) | 0.00 (0.00-0.00) | 0.00 (0.00-0.00) | 0.00 (0.00-0.00) | 0.00 (0.00-0.00) | 0.00 (0.00-0.00) | 0.01 (0.01-0.01) | |
| 10-14 | 0.16 (0.12-0.20) | 0.03 (0.02-0.04) | 0.00 (0.00-0.00) | 0.00 (0.00-0.00) | 0.04 (0.04-0.05) | 0.00 (0.00-0.00) | 0.00 (0.00-0.00) | 0.00 (0.00-0.00) | 0.00 (0.00-0.00) | 0.00 (0.00-0.00) | 0.01 (0.01-0.01) | |
| 15-19 | 0.16 (0.12-0.20) | 0.04 (0.04-0.05) | 0.00 (0.00-0.00) | 0.00 (0.00-0.00) | 0.05 (0.04-0.06) | 0.00 (0.00-0.00) | 0.00 (0.00-0.00) | 0.00 (0.00-0.00) | 0.00 (0.00-0.00) | 0.00 (0.00-0.00) | 0.01 (0.01-0.01) | |
| 20-24 | 0.16 (0.12-0.20) | 0.07 (0.06-0.07) | 0.00 (0.00-0.00) | 0.00 (0.00-0.00) | 0.05 (0.04-0.06) | 0.00 (0.00-0.00) | 0.00 (0.00-0.00) | 0.01 (0.01-0.01) | 0.00 (0.00-0.00) | 0.00 (0.00-0.00) | 0.02 (0.01-0.02) | |
| 25-29 | 0.16 (0.11-0.19) | 0.09 (0.08-0.10) | 0.00 (0.00-0.00) | 0.00 (0.00-0.00) | 0.05 (0.04-0.06) | 0.00 (0.00-0.00) | 0.00 (0.00-0.00) | 0.01 (0.01-0.02) | 0.00 (0.00-0.00) | 0.00 (0.00-0.00) | 0.02 (0.02-0.03) | |
| 30-34 | 0.15 (0.11-0.19) | 0.12 (0.10-0.14) | 0.00 (0.00-0.01) | 0.00 (0.00-0.00) | 0.05 (0.04-0.06) | 0.00 (0.00-0.00) | 0.00 (0.00-0.00) | 0.02 (0.02-0.03) | 0.00 (0.00-0.00) | 0.00 (0.00-0.00) | 0.02 (0.02-0.03) | |
| 35-39 | 0.15 (0.11-0.18) | 0.13 (0.11-0.15) | 0.01 (0.00-0.01) | 0.00 (0.00-0.01) | 0.05 (0.04-0.06) | 0.00 (0.00-0.01) | 0.00 (0.00-0.00) | 0.03 (0.02-0.03) | 0.00 (0.00-0.00) | 0.00 (0.00-0.00) | 0.03 (0.02-0.03) | |
| 40-44 | 0.15 (0.11-0.18) | 0.13 (0.11-0.16) | 0.01 (0.01-0.01) | 0.01 (0.00-0.01) | 0.05 (0.03-0.06) | 0.00 (0.00-0.01) | 0.00 (0.00-0.00) | 0.03 (0.02-0.04) | 0.00 (0.00-0.00) | 0.00 (0.00-0.00) | 0.03 (0.02-0.04) | |
| 45-49 | 0.15 (0.11-0.18) | 0.13 (0.11-0.16) | 0.02 (0.01-0.02) | 0.01 (0.00-0.01) | 0.05 (0.04-0.06) | 0.00 (0.00-0.01) | 0.00 (0.00-0.00) | 0.04 (0.03-0.05) | 0.00 (0.00-0.00) | 0.00 (0.00-0.00) | 0.03 (0.02-0.04) | |
| 50-54 | 0.15 (0.11-0.18) | 0.12 (0.09-0.15) | 0.02 (0.01-0.02) | 0.01 (0.00-0.01) | 0.05 (0.04-0.06) | 0.00 (0.00-0.01) | 0.00 (0.00-0.00) | 0.04 (0.03-0.05) | 0.00 (0.00-0.00) | 0.00 (0.00-0.00) | 0.03 (0.02-0.04) | |
| 55-59 | 0.15 (0.11-0.18) | 0.10 (0.08-0.14) | 0.03 (0.02-0.04) | 0.01 (0.00-0.01) | 0.05 (0.04-0.07) | 0.00 (0.00-0.01) | 0.00 (0.00-0.00) | 0.04 (0.03-0.06) | 0.00 (0.00-0.00) | 0.00 (0.00-0.00) | 0.03 (0.02-0.04) | |
| 60-64 | 0.15 (0.11-0.18) | 0.09 (0.06-0.13) | 0.04 (0.03-0.05) | 0.01 (0.00-0.01) | 0.04 (0.03-0.06) | 0.00 (0.00-0.00) | 0.01 (0.00-0.01) | 0.04 (0.03-0.06) | 0.00 (0.00-0.00) | 0.00 (0.00-0.00) | 0.03 (0.02-0.04) | |
| 65-69 | 0.14 (0.11-0.18) | 0.10 (0.07-0.15) | 0.04 (0.03-0.05) | 0.01 (0.00-0.01) | 0.04 (0.03-0.06) | 0.01 (0.00-0.01) | 0.01 (0.00-0.01) | 0.05 (0.03-0.07) | 0.00 (0.00-0.00) | 0.00 (0.00-0.01) | 0.03 (0.02-0.04) | |
| 70-74 | 0.14 (0.10-0.17) | 0.12 (0.06-0.17) | 0.04 (0.03-0.06) | 0.01 (0.00-0.01) | 0.04 (0.02-0.07) | 0.00 (0.00-0.01) | 0.01 (0.00-0.01) | 0.05 (0.04-0.08) | 0.00 (0.00-0.00) | 0.00 (0.00-0.01) | 0.03 (0.02-0.05) | |
| 75+ | 0.14 (0.10-0.17) | 0.15 (0.08-0.23) | 0.05 (0.03-0.08) | 0.01 (0.00-0.02) | 0.03 (0.01-0.07) | | 0.01 (0.00-0.02) | 0.08 (0.06-0.11) | | 0.01 (0.00-0.02) | 0.02 (0.01-0.03) | 0.01 (0.00-0.01) |

**Supplementary table 21.** Age-specific health loss proportion (HeLP) and 95% confidence interval for persons diagnosed with ADHD during 2000-2015. All estimates are adjusted for observed comorbidity from mental and substance use disorders and general medical conditions.

|  | Index disorder | Mental disorders | Circulatory diseases | Endocrine diseases | Pulmonary diseases | Gastrointestinal diseases | Urogenital diseases | Musculoskeletal diseases | Hematological diseases | Cancers | Neurological diseases | Other |
| --- | --- | --- | --- | --- | --- | --- | --- | --- | --- | --- | --- | --- |
| 0-4 | 0.04 (0.03-0.06) | 0.06 (0.04-0.07) | 0.00 (0.00-0.00) | | 0.03 (0.03-0.04) | |  | 0.00 (0.00-0.00) | 0.00 (0.00-0.00) | | 0.01 (0.01-0.01) | 0.00 (0.00-0.00) |
| 5-9 | 0.04 (0.03-0.06) | 0.05 (0.04-0.06) | 0.00 (0.00-0.00) | 0.00 (0.00-0.00) | 0.04 (0.03-0.05) | 0.00 (0.00-0.00) | 0.00 (0.00-0.00) | 0.00 (0.00-0.00) | 0.00 (0.00-0.00) | 0.00 (0.00-0.00) | 0.01 (0.00-0.01) | |
| 10-14 | 0.04 (0.03-0.06) | 0.05 (0.04-0.06) | 0.00 (0.00-0.00) | 0.00 (0.00-0.00) | 0.05 (0.04-0.06) | 0.00 (0.00-0.00) | 0.00 (0.00-0.00) | 0.00 (0.00-0.00) | 0.00 (0.00-0.00) | 0.00 (0.00-0.00) | 0.01 (0.01-0.01) | |
| 15-19 | 0.04 (0.03-0.06) | 0.06 (0.05-0.07) | 0.00 (0.00-0.00) | 0.00 (0.00-0.00) | 0.05 (0.04-0.07) | 0.00 (0.00-0.00) | 0.00 (0.00-0.00) | 0.00 (0.00-0.01) | 0.00 (0.00-0.00) | 0.00 (0.00-0.00) | 0.01 (0.01-0.01) | |
| 20-24 | 0.04 (0.03-0.06) | 0.10 (0.09-0.11) | 0.00 (0.00-0.00) | 0.00 (0.00-0.00) | 0.05 (0.04-0.06) | 0.00 (0.00-0.00) | 0.00 (0.00-0.00) | 0.01 (0.01-0.02) | 0.00 (0.00-0.00) | 0.00 (0.00-0.00) | 0.01 (0.01-0.01) | |
| 25-29 | 0.04 (0.03-0.06) | 0.15 (0.13-0.16) | 0.00 (0.00-0.00) | 0.00 (0.00-0.00) | 0.05 (0.04-0.07) | 0.00 (0.00-0.01) | 0.00 (0.00-0.00) | 0.03 (0.02-0.04) | 0.00 (0.00-0.00) | 0.00 (0.00-0.00) | 0.01 (0.01-0.01) | |
| 30-34 | 0.04 (0.03-0.05) | 0.17 (0.15-0.18) | 0.01 (0.00-0.01) | 0.00 (0.00-0.00) | 0.05 (0.04-0.07) | 0.01 (0.00-0.01) | 0.00 (0.00-0.00) | 0.05 (0.03-0.06) | 0.00 (0.00-0.00) | 0.00 (0.00-0.00) | 0.01 (0.01-0.01) | |
| 35-39 | 0.04 (0.03-0.05) | 0.17 (0.16-0.19) | 0.01 (0.01-0.01) | 0.00 (0.00-0.00) | 0.06 (0.05-0.07) | 0.01 (0.01-0.01) | 0.00 (0.00-0.00) | 0.06 (0.04-0.07) | 0.00 (0.00-0.00) | 0.00 (0.00-0.00) | 0.01 (0.01-0.01) | |
| 40-44 | 0.04 (0.03-0.05) | 0.16 (0.15-0.18) | 0.02 (0.01-0.02) | 0.00 (0.00-0.01) | 0.07 (0.05-0.08) | 0.01 (0.01-0.01) | 0.00 (0.00-0.00) | 0.06 (0.05-0.08) | 0.00 (0.00-0.00) | 0.00 (0.00-0.00) | 0.01 (0.01-0.02) | |
| 45-49 | 0.04 (0.03-0.05) | 0.15 (0.14-0.17) | 0.02 (0.02-0.03) | 0.01 (0.00-0.01) | 0.07 (0.05-0.08) | 0.01 (0.01-0.02) | 0.00 (0.00-0.00) | 0.07 (0.05-0.09) | 0.00 (0.00-0.00) | 0.00 (0.00-0.00) | 0.02 (0.01-0.02) | |
| 50-54 | 0.04 (0.03-0.05) | 0.15 (0.13-0.17) | 0.03 (0.03-0.04) | 0.01 (0.01-0.01) | 0.08 (0.06-0.09) | 0.01 (0.01-0.02) | 0.00 (0.00-0.00) | 0.07 (0.05-0.09) | 0.00 (0.00-0.00) | 0.00 (0.00-0.00) | 0.02 (0.01-0.02) | |
| 55-59 | 0.04 (0.03-0.05) | 0.15 (0.13-0.18) | 0.04 (0.03-0.05) | 0.01 (0.01-0.01) | 0.08 (0.06-0.10) | 0.01 (0.01-0.02) | 0.00 (0.00-0.01) | 0.08 (0.05-0.09) | 0.00 (0.00-0.00) | 0.00 (0.00-0.00) | 0.02 (0.01-0.03) | |
| 60-64 | 0.04 (0.03-0.05) | 0.12 (0.09-0.15) | 0.05 (0.03-0.06) | 0.01 (0.01-0.02) | 0.11 (0.08-0.13) | 0.01 (0.01-0.02) | 0.00 (0.00-0.01) | 0.09 (0.06-0.11) | 0.00 (0.00-0.00) | 0.01 (0.00-0.01) | 0.02 (0.01-0.03) | |
| 65-69 | 0.04 (0.03-0.05) | 0.10 (0.05-0.14) | 0.05 (0.03-0.07) | 0.02 (0.01-0.03) | 0.11 (0.07-0.15) | 0.01 (0.00-0.03) | | 0.09 (0.06-0.13) | |  | 0.01 (0.00-0.04) | 0.01 (0.00-0.02) |
| 70-74 | 0.04 (0.03-0.05) | 0.05 (0.01-0.11) | 0.05 (0.03-0.08) | | 0.09 (0.02-0.17) | |  | 0.09 (0.05-0.13) | |  | 0.03 (0.01-0.06) | 0.04 (0.01-0.08) |
| 75+ | 0.04 (0.03-0.05) | | 0.06 (0.04-0.17) | |  |  |  | 0.13 (0.05-0.17) | |  |  | 0.09 (0.05-0.27) |

**Supplementary table 22.** Age-specific health loss proportion (HeLP) and 95% confidence interval for persons diagnosed with conduct disorder during 2000-2015. All estimates are adjusted for observed comorbidity from mental and substance use disorders and general medical conditions.

|  | Index disorder | Mental disorders | Circulatory diseases | Endocrine diseases | Pulmonary diseases | Gastrointestinal diseases | Urogenital diseases | Musculoskeletal diseases | Hematological diseases | Cancers | Neurological diseases | Other |
| --- | --- | --- | --- | --- | --- | --- | --- | --- | --- | --- | --- | --- |
| 0-4 | 0.21 (0.16-0.29) | 0.06 (0.01-0.11) | |  |  |  |  |  |  |  |  | 0.13 (0.02-0.21) |
| 5-9 | 0.23 (0.17-0.30) | 0.04 (0.03-0.05) | |  | 0.04 (0.03-0.05) | |  | 0.00 (0.00-0.01) | |  | 0.00 (0.00-0.01) | 0.00 (0.00-0.00) |
| 10-14 | 0.23 (0.17-0.30) | 0.04 (0.03-0.05) | | 0.00 (0.00-0.00) | 0.04 (0.03-0.05) | |  | 0.00 (0.00-0.00) | |  | 0.01 (0.00-0.01) | 0.00 (0.00-0.00) |
| 15-19 | 0.22 (0.17-0.29) | 0.07 (0.05-0.08) | 0.00 (0.00-0.00) | 0.00 (0.00-0.00) | 0.05 (0.03-0.06) | |  | 0.00 (0.00-0.01) | 0.00 (0.00-0.00) | | 0.01 (0.00-0.01) | 0.00 (0.00-0.00) |
| 20-24 | 0.21 (0.16-0.28) | 0.12 (0.09-0.13) | 0.00 (0.00-0.00) | 0.00 (0.00-0.00) | 0.05 (0.03-0.06) | 0.00 (0.00-0.00) | | 0.01 (0.00-0.01) | |  | 0.00 (0.00-0.01) | 0.00 (0.00-0.00) |
| 25-29 | 0.20 (0.15-0.27) | 0.20 (0.12-0.24) | 0.00 (0.00-0.01) | | 0.06 (0.03-0.07) | 0.00 (0.00-0.01) | | 0.03 (0.01-0.04) | |  | 0.01 (0.00-0.01) | 0.00 (0.00-0.01) |
| 30-34 | 0.19 (0.15-0.25) | 0.19 (0.14-0.32) | |  | 0.05 (0.01-0.06) | |  | 0.05 (0.02-0.09) | |  | 0.02 (0.00-0.03) | 0.01 (0.00-0.03) |
| 35-39 | 0.18 (0.14-0.25) | 0.30 (0.12-0.35) | |  | 0.06 (0.01-0.09) | |  | 0.05 (0.02-0.08) | |  |  | 0.02 (0.00-0.05) |
| 40-44 | 0.18 (0.13-0.24) | 0.21 (0.11-0.39) | |  | 0.07 (0.02-0.14) | |  | 0.08 (0.02-0.11) | |  |  | 0.06 (0.01-0.09) |
| 45-49 | 0.17 (0.12-0.25) | 0.20 (0.05-0.31) | 0.03 (0.00-0.08) | |  |  |  | 0.07 (0.03-0.14) | |  |  | 0.11 (0.02-0.23) |
| 50-54 | 0.15 (0.09-0.23) | 0.25 (0.05-0.43) | |  |  |  |  |  |  |  |  | 0.30 (0.10-0.43) |
| 55-59 |  |  |  |  |  |  |  |  |  |  |  |  |
| 60-64 | 0.21 (0.15-0.28) | |  |  |  |  |  |  |  |  |  | 0.19 (0.04-0.39) |
| 65-69 | 0.20 (0.14-0.27) | |  |  |  |  |  |  |  |  |  | 0.32 (0.07-0.59) |
| 70-74 |  |  |  |  |  |  |  |  |  |  |  |  |
| 75+ | 0.17 (0.13-0.23) | 0.12 (0.04-0.19) | 0.07 (0.03-0.16) | |  |  |  |  |  |  |  | 0.23 (0.11-0.37) |

**References:**

1. GBD 2017 Disease and Injury Incidence and Prevalence Collaborators. Global, regional, and national incidence, prevalence, and years lived with disability for 354 diseases and injuries for 195 countries and territories, 1990–2017: a systematic analysis for the Global Burden of Disease Study 2017. *The Lancet* 2018; **392**(10159): 1789-858.
